# Polypyrimidine tract binding proteins PTBP1 and PTBP2 interact with distinct proteins under splicing conditions

**DOI:** 10.1371/journal.pone.0263287

**Published:** 2022-02-03

**Authors:** Jeffrey M. Pina, Luis A. Hernandez, Niroshika M. Keppetipola

**Affiliations:** 1 Department of Chemistry and Biochemistry, California State University Fullerton, Fullerton, CA, United States of America; 2 Department of Biological Sciences, California State University Fullerton, Fullerton, CA, United States of America; Panjab University Chandigarh, INDIA

## Abstract

RNA binding proteins play an important role in regulating alternative pre-mRNA splicing and in turn cellular gene expression. Polypyrimidine tract binding proteins, PTBP1 and PTBP2, are paralogous RNA binding proteins that play a critical role in the process of neuronal differentiation and maturation; changes in the concentration of PTBP proteins during neuronal development direct splicing changes in many transcripts that code for proteins critical for neuronal differentiation. How the two related proteins regulate different sets of neuronal exons is unclear. The distinct splicing activities of PTBP1 and PTBP2 can be recapitulated in an in vitro splicing system with the differentially regulated N1 exon of the c-src pre-mRNA. Here, we conducted experiments under these in vitro splicing conditions to identify PTBP1 and PTBP2 interacting partner proteins. Our results highlight that both PTBPs interact with proteins that participate in chromatin remodeling and transcription regulation. Our data reveal that PTBP1 interacts with many proteins involved in mRNA processing including splicing regulation while PTBP2 does not. Our results also highlight enzymes that can serve as potential “writers” and “erasers” in adding chemical modifications to the PTB proteins. Overall, our study highlights important differences in protein-protein interactions between the PTBP proteins under splicing conditions and supports a role for post-translational modifications in dictating their distinct splicing activities.

## Introduction

Related members in RNA binding protein families have distinct tissue-specific expression patterns and exert over-lapping and discrete splicing outcomes on certain regulated exons to facilitate tissue-specific gene expression patterns [1, 2]. How paralogs can exert distinct splicing outcomes is not well understood. The polypyrimidine tract binding proteins, PTBP1 and PTBP2, are paralogous RNA binding proteins [2, 3]. PTBP1 and 2 most often function to repress splicing of regulated exons but can also enhance splicing of exons [4–12]. The two proteins exhibit overlapping and distinct splicing outcomes on regulated exons. Specifically, PTBP1 represses inclusion of certain regulated exons in neuronal transcripts such as the c-Src N1 exon and exon 9 of GABA_A_ receptor γ2 subunit while PTBP2 does not [13, 14]. The paralogs also exhibit tissue specific expression patterns where PTBP1 is expressed in most cell types and neuronal progenitor cells, whereas PTBP2 is expressed primarily in differentiating neurons and testis [2]. During neuronal development, the level of PTBP1 decreases with a corresponding increase in PTBP2 levels [4, 5, 15]. This change in protein concentrations alters the splicing of exons that are differentially regulated by the paralogs to generate protein isoforms critical for neuronal differentiation [9, 16, 17]. Later, during neuronal maturation, the level of PTBP2 also decreases and this leads to the generation of protein isoforms important for neuronal maturation [5–7, 18, 19]. Mouse PTBP2 ^-^/^-^ brains show extensive changes in alternative splicing [5, 6, 9]. PTBP1 can compensate for the loss of PTBP2 during neuronal development for selected splicing events in some development contexts only but not others [9]. Thus, it is clear the paralogs play distinct roles in the process of neuronal development. How PTBP1 and PTBP2 can selectively regulate different sets of target exons is not understood; once known, it would enhance our understanding of the pathways and proteins involved in regulating neuronal gene expression.

**Fig 1:**
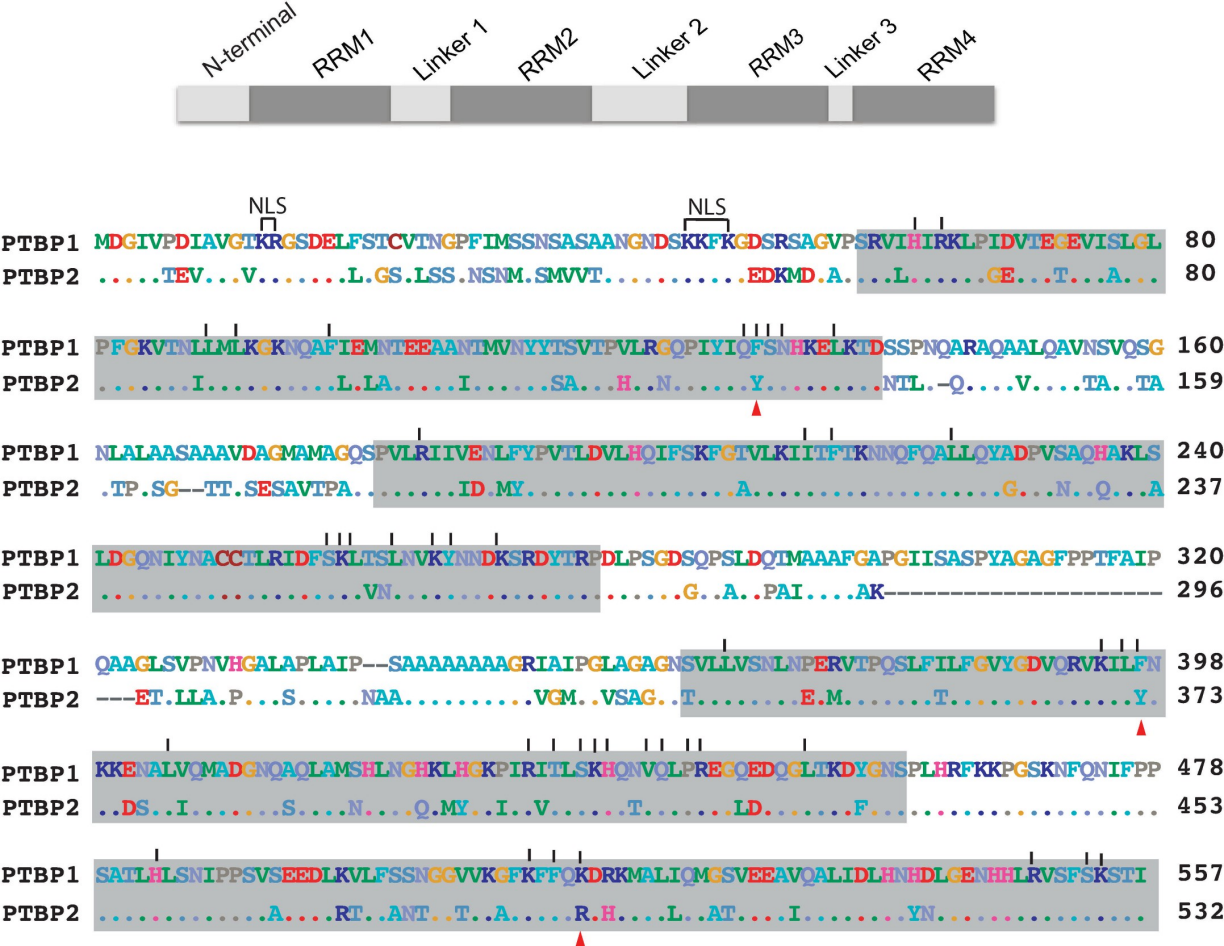
PTBP domain organization and amino acid sequence alignment. (top) A schematic representation of PTB protein indicating each distinct region. (bottom) Amino acid sequence alignment of human PTBP1 (isoform 4) and PTBP2. Gray boxes indicate RNA binding domains. Lines above the sequence indicate RNA interacting residues. Red triangles below the sequence indicate RNA interacting residues that are conservative substitutions in PTBP2. Gaps are indicated as dashes (-) in the alignment.

PTBP1 and PTBP2 are 74% identical in amino acid sequence and have a similar domain organization of four RNA binding domains (RBDs) connected by three linker regions (Fig 1 top). There is also an N-terminal region that contains both a nuclear localization and export sequence (Fig 1 bottom). The paralogs share greater than 80% sequence identity over the RBDs and 100% identity over the Linker 3 region (Fig 1 top). A carboxy-terminal fragment of PTBP2, containing RBD3, Linker 3, and RBD4, is very similar in tertiary structure to the same region of PTBP1 [20]. Solution structures of each PTBP1 RBD bound to a CUCUCU hexamer identified residues important for RNA recognition and binding [21–25]. PTBP2 has the same residues at these positions of RNA contact except for one lysine to arginine and two phenylalanine to tyrosine substitutions (Fig 1 bottom).

Thus, the paralogs are highly similar in structure over the RBD’s, and residues involved in recognition and binding to target RNA sequences. *In vivo* chromatin immunoprecipitation sequencing studies conducted to examine RNA binding across the transcriptome, highlighted that the two proteins can recognize and bind to the same RNA sequences independent of the regulation specificity [9]. These findings highlight the paralogs can recognize and bind to the same CU rich sequence elements *in vivo* and suggest that additional factors such as post-translational modifications and/or protein-protein interactions might play a role in their ability to differentially regulate certain target exons. Indeed, we discovered the two proteins have overlapping and distinct acetate and phosphate modifications under splicing conditions respectively. Distinct phosphate modifications are localized to the regions less conserved between the paralogs including the N-terminal, Linker 1 and Linker 2 regions (Fig 1) [26]. Whether differences in phosphate modifications play a role in PTBP distinct splicing activity is currently under investigation in the laboratory. Here, we aimed to determine PTBP1 and PTBP2 protein-protein interactions that occur under splicing conditions to identify distinct partner proteins that might play a role in their differential splicing activities. To this end, we used a previously established [27] in vitro splicing system that recapitulates the differential splicing regulation of PTBP1 and PTBP2 to identify protein-protein interactions that occur under these conditions.

## Results and discussion

### PTBP1 and PTBP2 interact with proteins involved in RNA processing and chromatin remodeling under in vitro splicing conditions

**Fig 2:**
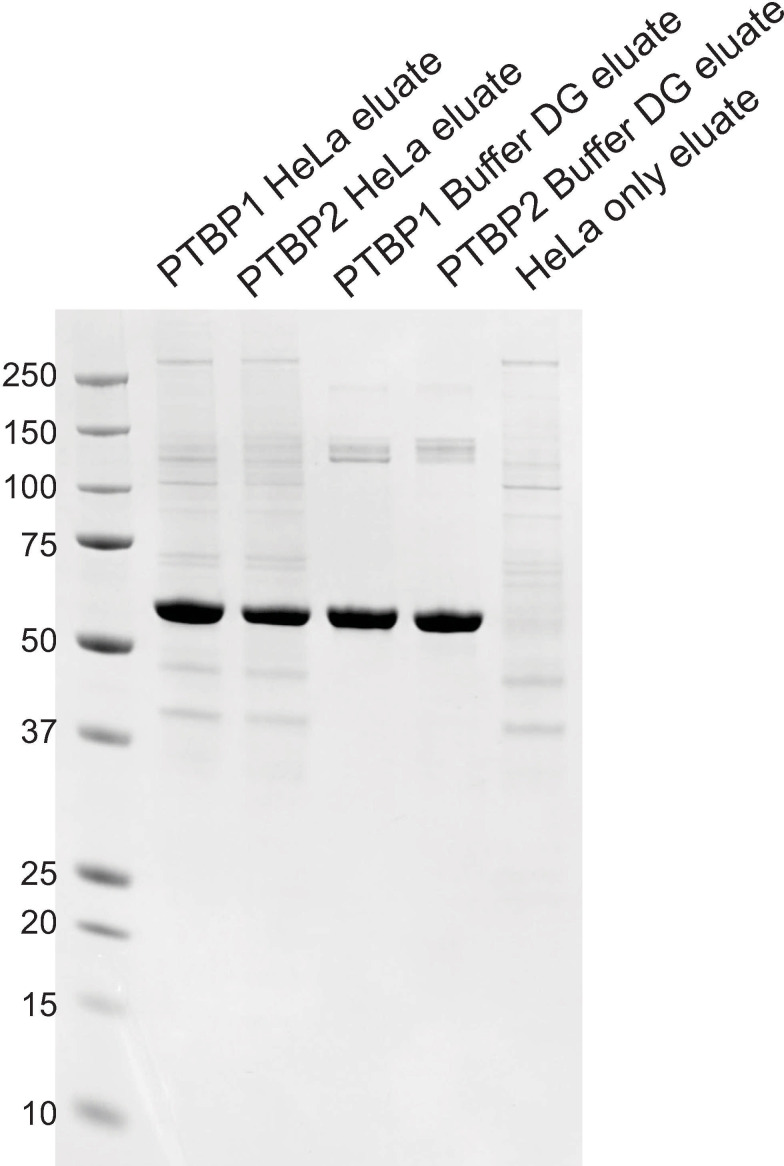
Purification of His-_6_ tagged PTBP from splicing reaction mixtures. Splicing reaction mixtures contained 2.2mM MgCl_2_, 0.4mM ATP and 20mM Creatine Phosphate and either HeLa nuclear extract or Buffer DG. Aliquots (10 μl) of the indicated fractions were analyzed by SDS-PAGE. The gel was stained with Gel Code blue safe stain. The positions and sizes (kilodaltons) of marker polypeptides are shown on the left.

Recombinant expressed His_6_-tagged PTBP1 and PTBP2 were incubated under in vitro buffer conditions known to promote splicing containing HeLa nuclear extract as described previously [26] and His_6_-PTBPs were purified from these reaction mixtures. Proteins incubated under splicing conditions containing the dialysis buffer used in extract preparation (Buffer DG) [28] served as controls. We included an additional control reaction that contained all components of the splicing reaction mixture including HeLa nuclear extract, without the addition of PTB proteins. This control served to determine unspecifically bound proteins present in the eluates. Elution fractions were analyzed for purity and the presence of co-purified proteins via SDS-PAGE Coomassie stain (Fig 2). An intense band at ~57 kDa in all lanes confirm the presence of recombinant PTBP1 and PTBP2 in all elution fractions except the HeLa only control. We observe the presence of many proteins in the HeLa only control indicative of unspecifically bound proteins. We analyzed the protein composition of each of the eluates via mass spectrometry.

To determine distinct proteins that interact with PTBP1 under splicing conditions containing HeLa nuclear extract, we removed proteins present in PTBP1 Buffer DG indicative of proteins that carried over from bacterial recombinant expression and purification (S1 Table). We also removed proteins present in the HeLa extract only control indicative of unspecific binding of proteins to the magnetic beads during the pull-down assay (S2 Table). We conducted a similar analysis for PTBP2 to determine proteins that interact specifically with PTBP2 under splicing conditions containing HeLa nuclear extract. Proteins found present in the PTBP2 Buffer DG sample are listed in S3 Table. We then removed actin, keratin and cytoskeleton related proteins that are considered common contaminants from each list [29, 30] (S4 Table) to obtain lists of relevant proteins that specifically bound to PTBP1 and PTBP2 under the in vitro splicing conditions. Comparison of the two resultant lists highlighted 17 proteins that co-purified with both PTBP1 and PTBP2 under these conditions. These proteins are involved in several biological processes and functions related to PTBP (per Gene Ontology analysis) including mRNA processing, chromatin binding and remodeling, transcriptional regulation and ATP binding (S5 Table).

**Fig 3:**
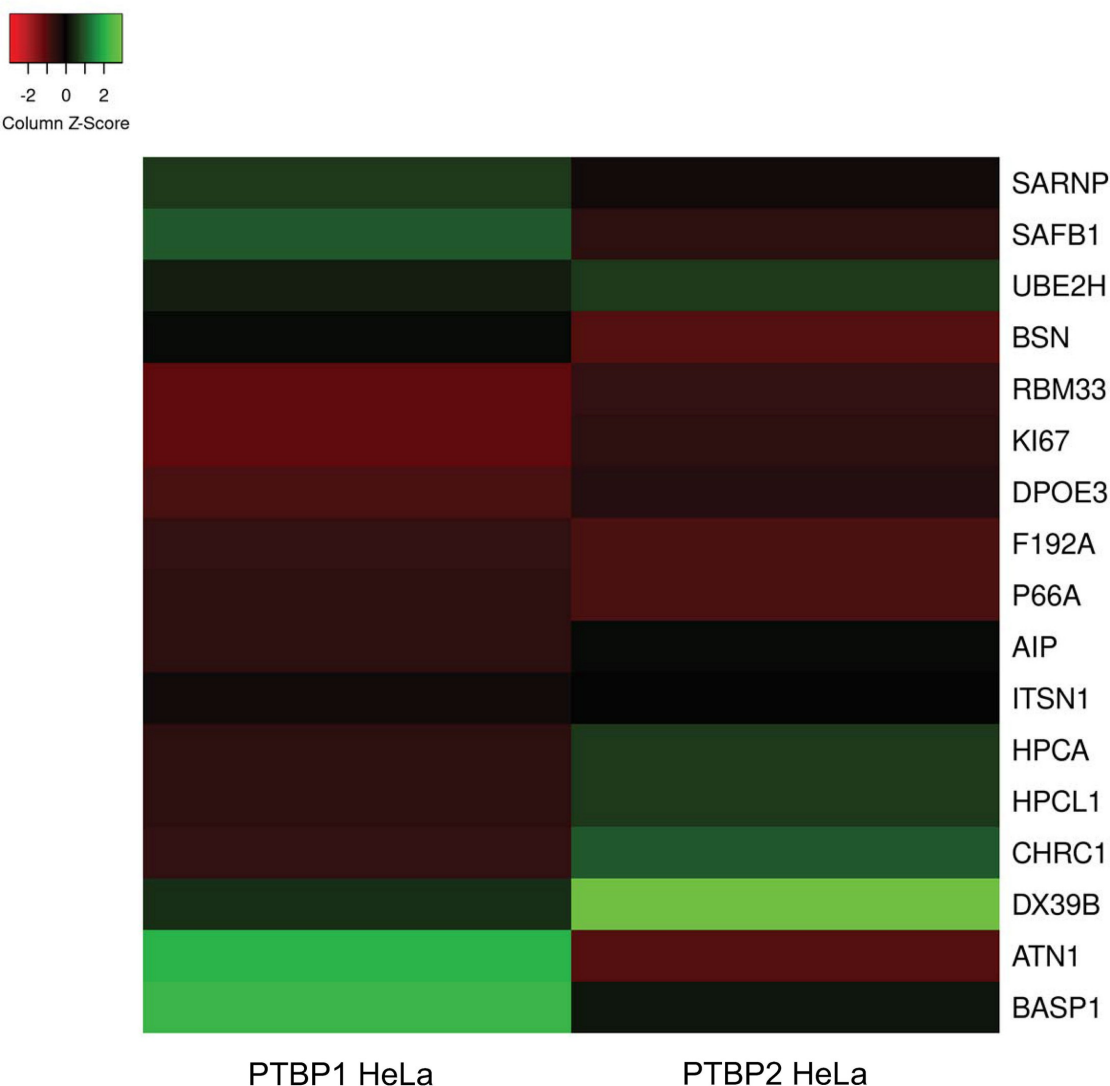
Heat map depicting the relative prevalence of the 17 proteins pulled down with both PTBP1 and PTBP2. The 0 baseline, indicated by black color shading, was determined by taking the average of the area scores for all 17 proteins. Proteins with a higher or lower area score than this average are shaded green or red respectively, with each point on the scale indicating the number of standard deviations from the baseline. This heat map was generated via heatmapper.ca [31].

We generated a Heatmap [31] to visually compare and determine differences in abundance of each protein co-purified with both PTBP1 and PTBP2 (Fig 3).

The average abundance of a protein (indicated by a score of zero and black color in the scale) is calculated by obtaining the sum of the area of all proteins and dividing by the number of proteins. Green and red bars (and corresponding intensities of those colors) indicate areas that are a standard deviation higher or lower than the mean respectively. In comparing the abundances, we note that SAFB1 and ATN1 are more prevalent in the presence of PTBP1 compared to PTBP2. SAFB1 is involved in chromatin binding and RNA/DNA binding. ATN1 is also involved in protein domain specific binding and plays a role in transcription co-repressor activity. We note that SARNP and BASP1 are also more prevalent in the PTBP1 sample albeit to a lesser extent. These proteins play a role in transcription corepressor activity, RNA binding, and chromatin binding. We surmise the presence of these proteins is due to pre-mRNA splicing being co-transcriptional and likely involves the interaction of splicing factors such as PTBPs with proteins involved in chromatin rearrangement and transcription [32]. Higher abundance of these proteins in the PTBP1 sample indicate an increased affinity of these proteins to PTBP1.

Proteins DX39B, CHRC1, HPCL1, HPCA and UBE2H are more prevalent in the PTBP2 HeLa sample indicating higher affinity to PTBP2. DX39B functions as an ATP-ase and has ATP-dependent protein binding, RNA binding and RNA-dependent ATPase activity. CHRC1 is a DNA binding protein involved in chromatin remodeling. HPCL1 and HPCA play a role in calcium ion binding. UBE2H has ubiquitin conjugating enzyme activity. Thus, these proteins also seem to collectively play a role in splicing and transcription regulation.

Proteins BSN (metal ion binding), RBM33 (RNA binding), KI67 (ATP, DNA, RNA binding), DPOE3 (chromatin DNA binding), P66A (protein-macromolecule adaptor activity), AIP (peptidyl prolyl cis-trans isomerase activity) and ITSN1 (kinase activator activity) co-purified with both PTBP1 and PTBP2 with lower abundance relative to the mean (corresponding to black and red bars). Overall, our results indicate that both PTBP1 and PTBP2 interact in common with a few proteins involved in mRNA processing, transcription and chromatin remodeling under in vitro splicing conditions. PTBP1 is known to interact with chromatin-binding protein MRG15 in regulating the splicing of a sub-set of its target genes [33]. Our results suggest that other combinations of adaptor systems that read histone modifications and interact with the PTB proteins to transmit the epigenetic information to the splicing machinery may exist.

### Distinct PTBP1 interacting partner proteins under splicing conditions

PTBP1 and PTBP2 have distinct splicing outcomes on the neuronal c-Src N1 exon where PTBP1 represses N1 exon inclusion in the spliced mRNA while PTBP2 does not [13]. We note this may be due to differences in protein-protein interactions that influence the assembly of a functional spliceosome at the adjacent splice sites. We analyzed our results to address if the paralogs have distinct protein-protein interactions under splicing conditions.

**Fig 4:**
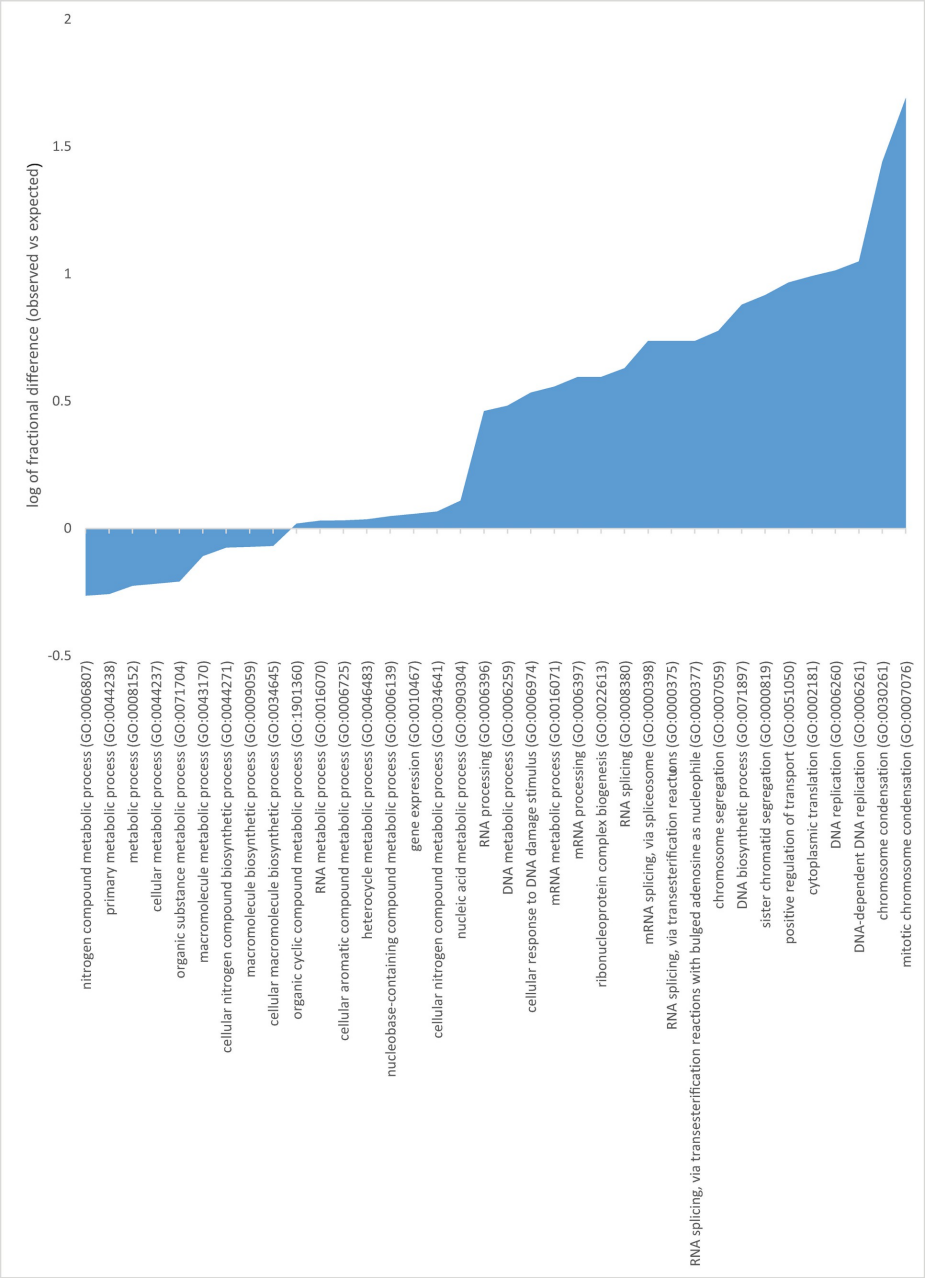
Log fractional difference of observed vs expected GO SLIM biological process categories assigned to proteins found unique to the PTBP1 pulldown. The log fractional difference is calculated for each category as (# genes for the category—# genes expected) / # genes expected) as provided on Gene Ontology.

Our data highlight 145 distinct proteins that interact with PTBP1 (S6 Table). Gene Ontology analysis of these co-purified proteins reveal they participate in several processes including chromosome condensation, DNA replication, cytoplasmic translation, mRNA processing and cellular metabolic processes. We note that a given protein could be in one or more of these categories. We also analyzed the data utilizing Gene Ontology’s manually curated GO SLIM Biological Processes data set. In contrast to complete GO analysis, which is both manually and electronically curated, GO SLIM utilizes only experimentally validated classifications of submitted proteins allowing for more streamlined interpretation. This analysis returned 36 significant GO SLIM Biological Process terms (Fig 4). Terms of note include RNA splicing (GO:0008380), mRNA splicing via spliceosome (GO:0000398), and RNA splicing via transesterification reactions (GO:0000375), which have greater representation than the expected reference dataset. As these terms are directly related to known PTBP1 functions, the proteins within these categories were further examined. Of note is the large prevalence or chromosomal organizational proteins, with chromosome condensation (GO:0030261) and mitotic chromosome condensation (GO:0007076) having 27 and 54 fold enrichment respectively. We note some splicing factors regulate centromeric RNAs by directly controlling interactions among kinetochores, spindle microtubules and the Ndc80 complex [34]. Our GO SLIM analysis results suggest a yet-to-be discovered role for PTBP1 in chromosomal organization.

Given PTBP1’s role in alternative splicing, polyadenylation, mRNA stability and IRES-mediated translation initiation, we focused our analysis on co-purified proteins related to mRNA processing and translation [3, 35]. Ribonucleoprotein complex assembly proteins include U1 snRNPC; a component of U1 snRNP which is essential for recognition of the 5’ splice site and subsequent spliceosome assembly in both constitutive and regulated alternative splicing, splicing factors SART3 (a component of U6 snRNP binding protein), ISY1 (component of the spliceosome C complex), translation initiation factors EIF3A (RNA binding component of the eukaryotic translation initiation factor eIF3), EIF3I (also a component of the eukaryotic translation initiation factor 3 complex eIF3 which is required for several steps in the initiation of protein synthesis) and ribosomal proteins RPLP0P6 and RPLP0. ATP-dependent RNA helicase DDX3X, exoribonucleases RRP44 and RRP41 and ubiquitin carboxyl terminal hydrolase USP4 were categorized as those involved in ribonuleoprotein complex biogenesis. We observed significant overlap between proteins in categories related to ribonucleoprotein complex processes.

Looking into proteins related to mRNA processing, in addition to some already mentioned above, we note the presence of LSM7 which plays a role in pre-mRNA splicing as a component of the U4/U6 -U5 tri-snRNP complex and as a component of the precatalytic spliceosome B complex, SNRNP27 which may play a role in splicing and splicing factor RBMXL3. Additional proteins that fell into RNA processing and splicing categories included Serine–Arginine related protein 53 (RSRC1) which plays a role in constitutive and alternative splicing and protein phosphatase PPP2CA.

Fifty-nine of the distinct proteins that co-purified with PTBP1 play a role in regulation of gene expression. In addition to those discussed above, we note the presence of general transcription factors (CCR4-NOT, RYBP, ARX, WIZ, OLIG2 and PBX1 among others), exoribonucleases (HMGA1), enzymes involved in reversible covalent modifications (KMT2A, MAP3K4, PPP2CB, PPP2CA and EIF2AK4), proteins involved in RNA metabolism (CAPRIN1, SPEN, PSPC1) and membrane trafficking and transporter proteins (NUP214 and ERC1). Collectively, our results highlight that i) PTBP1 interacts with proteins important for pre-mRNA splicing and translation initiation in line with its well-characterized functions ii) PTBP1 may play a role in chromosome condensation and segregation during mitosis, iii) PTBP1 interacts with transcription factors in agreement with co-transcriptional alternative splicing [36] and iv) candidate enzymes responsible for PTBP1 reversible modification. These findings support previously well-characterized PTBP1 functions and highlight novel interactions that raise important questions to be addressed in future studies.

### Distinct PTBP2 interacting partner proteins under splicing conditions

We analyzed the PTBP2 data in a similar manner to determine distinct protein-protein interactions under in vitro splicing conditions. Our analysis reveals 95 distinct proteins co-purified with PTBP2 under splicing conditions (S7 Table). Gene Ontology analysis based on biological function categorized these proteins into only two main categories; histone modification and positive regulation of cellular and macromolecular biosynthetic processes. GO SLIM analysis for biological processes was also carried out. In contrast to PTBP1 GO SLIMs, PTBP2 returned only 1 significant category: macromolecule metabolic process (GO:0043170). As this category is very broad and provides no insight to specific biological functions or pathways, we were unable to make further interpretations based on the PTBP2 GO SLIM data. The smaller number of unique proteins that were bound to PTBP2, in comparison to PTBP1, likely contributed in part to the lack of additional significant GO SLIM categories.

Histone modification proteins include nuclear receptor coactivator 3 (NCOA3), histone methyltransferases ASH2, KMT2C, histone deubiquitinase MYSM1, non-receptor serine/threonine protein kinase MAP3K12, lysine demethylase RSBN1 as well as proteins associated with chromatin remodeling such as BAZ2A and BRPF3. PTBP2 is post-translationally modified by chemical groups including phosphate, acetate and ubiquitin among others [26]. The role of phosphate modifications in PTBP2 splicing activity and related enzymes are currently under investigation in our laboratory. MAP3K12 is expressed in neuronal cells and is part of the kinase signaling pathway that is known to activate downstream targets including transcription factors that are involved in cellular stress response. It will be interesting to examine whether activated MAP3K12 phosphorylates PTBP2 to regulate its function in differentiating neurons. Our data suggests enzymes involved in histone modifications may also play a role in PTBP2 post-translational modification.

PTBP2 interacts with many proteins involved in positive regulation of cellular and macromolecular biosynthetic processes. Many of the histone modification proteins occur in this category as well. Some unique proteins include DNA binding transcription factor (GATA4), telomerase reverse transcriptase TERT, chromodomain helicase CHD8 among other transcription factors. Surprisingly, none of the distinct co-purified proteins fell into a category directly related to splicing, in contrast to PTBP1. Given its expression in neuronal cells, it is plausible that PTBP2 interacts more with splicing related proteins from neuronal cell nuclear extracts than HeLa nuclear extracts.

## Conclusion

Collectively, our study reveals that under in vitro splicing conditions where PTBP1 but not PTBP2 represses the inclusion of the neuronal N1 exon, the paralogs have distinct interacting partner proteins. Our data reveal that both PTB proteins interact with proteins that participate in chromatin remodeling and transcription regulation. Notably PTBP1, but not PTBP2, interacts with known splicing factors to carry out the observed N1 exon splicing repression. Our results highlight that PTBP2 does not interact with splicing factors and we surmise that this may lead to the observed inclusion of the N1 exon in the spliced mRNA [27]. Given the 74% sequence identity between the paralogs, it is intriguing they have distinct protein-protein interactions. This finding is in alignment with our previous observations where the paralogs interacted with partner protein Raver 1 with distinct affinities (PTBP1 RRM2 interacted with Raver 1 eight fold more than PTBP2 RRM2) albeit the greater than 90% sequence identity over the interaction motif [27]. We note the presence of co-purified enzymes responsible for reversible modifications with both PTBP1 and PTBP2; our previous work demonstrated the two proteins are acetylated and phosphorylated under these splicing conditions [26]. Our data reveal candidate enzymes that may serve as potential “writers” and “erasers” in adding and removing chemical groups from the PTB proteins. Collectively, our study supports the notion that protein-protein interactions likely dictate the distinct splicing activities of paralogous RNA binding proteins. Our findings also suggest a role for post-translational modifications in mediating these distinct interactions.

## Materials and methods

Expression and purification of His-tagged PTBP proteins in *E*.*coli* was carried out as previously described [26] with the addition of RNAse A (5 ug / ml) (Fisher Scientific) after resuspension in Binding Buffer and the Wash Buffer contained 750 mM NaCl to reduce unspecific binding of nucleic acids.

### Purification of His-tagged PTBP proteins from splicing reaction mixtures for mass spectrometry analysis

HeLa nuclear extracts were prepared as previously described [26, 28]. Splicing reactions contained 2.2 mM MgCl_2_, 0.4 mM ATP, 20 mM creatine phosphate, 48 ug of histidine tagged PTBP, and were brought to a total volume of 200 μl with HeLa nuclear extracts (PTBP1 HeLa, PTBP2 HeLa) or with Buffer Dialysis Glutamate (Buffer DG) that contained 80 mM KCl instead of 80 mM monopotassium glutamate (20 mM HEPES KOH pH 7.9, 20% glycerol, 80 mM KCl, 0.2 mM PMSF, 1 mM DTT) for controls (PTBP1 DG, PTBP2 DG). A splicing reaction that contained all components including HeLa extract but not PTBP protein was also used as a control to identify non-specific proteins inherent to HeLa nuclear extract (HeLa only). Reaction tubes were incubated at 30°C for 90 minutes. All steps described here in were carried out at 4°C. After incubation,100 μl HisPur Ni-NTA magnetic resin (Thermo Fisher), equilibrated in Buffer DG with 1X Phosstop (Sigma Aldrich) and 1X Sigmafast EDTA free (Sigma Aldrich), was added to each reaction mixture. An additional 200 μl of Buffer DG with 1X Phosstop and 1X Sigmafast was added to each sample and set to incubate overnight. A magnetic separation rack (New England Biolabs) was utilized to remove the buffer. Resin was washed once with 1 ml of Wash Buffer (50 mM sodium phosphate pH 8.0, 150 mM NaCl, 30 mM imidazole, 1X Phosstop, 1X Sigmafast EDTA free) followed by three 1 ml washes with Wash Buffer without 1X Phosstop and 1X Sigmafast EDTA free. 60 μl of Elution Buffer (50 mM sodium phosphate pH 8.0, 150 mM NaCl, 300 mM imidazole) was utilized to resuspend the resin and the samples were set to elute overnight. Supernatant was retrieved via magnetic separation and was dialyzed utilizing Slide-a-lyzer mini units (2000 MWCO) (Fisher Scientific) for 2 hours in 1 L of dialysis buffer (20 mM HEPES KOH pH 7.9, 80 mM KCl, 1mM DTT, 0.1 mM PMSF), changing to fresh buffer at the 1 hour mark. To determine PTBP specific protein-protein interactions that occur under splicing conditions, eluates equivalent to 5 μg of protein were aliquoted and sent for mass spectrometry analysis to the University of California San Diego, Biomolecular and Proteomics Mass Spectrometry facility.

### Mass spectrometry methods

#### Sample preparation

Protein samples were diluted in TNE (50 mM Tris pH 8.0, 100 mM NaCl, 1 mM EDTA) buffer. RapiGest SF reagent (Waters Corp.) was added to the mix to a final concentration of 0.1% and samples were boiled for 5 min. TCEP (Tris (2-carboxyethyl) phosphine) was added to 1 mM (final concentration) and the samples were incubated at 37°C for 30 min. Subsequently, the samples were carboxymethylated with 0.5 mg/ml of iodoacetamide for 30 min at 37°C followed by neutralization with 2 mM TCEP (final concentration). Proteins samples prepared as above were digested with trypsin (trypsin:protein ratio—1:50) overnight at 37°C. RapiGest was degraded and removed by treating the samples with 250 mM HCl at 37°C for 1 h followed by centrifugation at 14000 rpm for 30 min at 4°C. The soluble fraction was then added to a new tube and the peptides were extracted and desalted using C18 desalting columns (Thermo Scientific, PI-87782). Peptides were quantified using BCA assay and a total of 1 ug of peptides were injected for LC-MS analysis.

Trypsin-digested peptides were analyzed by ultra high pressure liquid chromatography (UPLC) coupled with tandem mass spectroscopy (LC-MS/MS) using nano-spray ionization. The nanospray ionization experiments were performed using a Orbitrap fusion Lumos hybrid mass spectrometer (Thermo) interfaced with nano-scale reversed-phase UPLC (Thermo Dionex UltiMate™ 3000 RSLC nano System) using a 25 cm, 75-micron ID glass capillary packed with 1.7-μm C18 (130) BEH^TM^ beads (Waters corporation). Peptides were eluted from the C18 column into the mass spectrometer using a linear gradient (5–80%) of ACN (Acetonitrile) at a flow rate of 375 μl/min for 1.5 h. The buffers used to create the ACN gradient were: Buffer A (98% H_2_O, 2% ACN, 0.1% formic acid) and Buffer B (100% ACN, 0.1% formic acid). Mass spectrometer parameters are as follows; an MS1 survey scan using the orbitrap detector (mass range (m/z): 400–1500 (using quadrupole isolation), 120000 resolution setting, spray voltage of 2200 V, Ion transfer tube temperature of 275 C, AGC target of 400000, and maximum injection time of 50 ms) was followed by data dependent scans (top speed for most intense ions, with charge state set to only include +2–5 ions, and 5 second exclusion time, while selecting ions with minimal intensities of 50000 at in which the collision event was carried out in the high energy collision cell (HCD Collision Energy of 30%), and the fragment masses where analyzed in the ion trap mass analyzer (With ion trap scan rate of turbo, first mass m/z was 100, AGC Target 5000 and maximum injection time of 35ms). Protein identification was carried out using Peaks Studio 8.5 (Bioinformatics solutions Inc.) [37, 38].

#### Data analysis methods

PEAKS 8.5 software was utilized by the mass spectroscopy facility to export the data into an Excel format. For each sample only proteins with an “Area” greater than 0 were utilized. To determine unique proteins found in each PTBP HeLa sample, the proteins found in the HeLa only control and the DG samples were removed from their respective PTBP1 HeLa or PTBP2 HeLa list using Excel matching functions. Cytoskeletal proteins (actin, myosin, and tubulin) were removed from this list via Gene Ontology Biological Process annotations to produce a usable list of unique proteins found in PTBP1 HeLa and PTBP2 HeLa samples respectively. The protein lists were then uploaded into the Gene Consortium Gene Ontology website to compare similarities and differences between the types of proteins found in each sample. This process was repeated in order to determine the common proteins found in both PTBP1 HeLa and PTBP2 HeLa samples. Heatmapper.ca was utilized to produce a heat map to compare the “Area” values of the common PTBP HeLa proteins. The mass spectrometry proteomics data have been deposited to the ProteomeXchange Consortium via the PRIDE [39] partner repository with the dataset identifier PXD029899.

### SDS-PAGE

5 ul of dialyzed elution fractions were prepared for electrophoresis by adding Laemmli SDS-gel loading dye and heating on a heat block at 95°C for 2 minutes. Samples were loaded into a NuPAGE 4–12% Bis-Tris gel (Thermo Scientific) for gel electrophoresis at 60V for 45 minutes followed by 120V for 1 hour 30 minutes. The gel was stained using Coomassie Blue stain.

## Supporting information

S1 Table Proteins identified in PTBP1 Buffer DG sample. A list of proteins identified in the pull-down sample in the presence of His6 tagged PTBP1 under Buffer DG conditions.
(PDF)

S2 Table Proteins identified in the HeLa extract only sample. A list of proteins in HeLa nuclear extract that unspecifically bind to the Ni-NTA beads.
(PDF)

S3 Table Proteins identified in PTBP2 Buffer DG sample. A list of proteins identified in the pull-down sample in the presence of His6 tagged PTBP2 under Buffer DG conditions.
(PDF)

S4 Table Common contaminants in mass spectrometry assays. A list of proteins considered common contaminants in mass spectrometry assays.
(PDF)

S5 Table Common co-purified proteins with PTBP1 and PTBP2. A list of proteins that co-purified with both PTBP1 and PTBP2 under in vitro splicing conditions containing HeLa nuclear extract.
(PDF)

S6 Table Distinct PTBP1 interacting proteins. A list of distinct proteins that co-purified with PTBP1 under in vitro splicing conditions containing HeLa nuclear extract.
(PDF)

S7 Table Distinct PTBP2 interacting proteins. A list of distinct proteins that co-purified with PTBP2 under in vitro splicing conditions containing HeLa nuclear extract.
(PDF)

S1 Raw images (PDF)
