# Supplementary material for: Polypyrimidine tract binding proteins PTBP1 and PTBP2 interact with distinct proteins under splicing conditions
Source: PLoS One. 2022 Feb 3;17(2):e0263287. doi: 10.1371/journal.pone.0263287 (PMC8812845; doi:10.1371/journal.pone.0263287)
Supplement: S1 Table — A list of proteins identified in the pull-down sample in the presence of His6 tagged PTBP1 under Buffer DG conditions. (PDF) [file pone.0263287.s001.pdf]

UniProtID	Gene	Description
P26599	PTBP1	Polypyrimidine tract-binding protein 1 OS=Homo sapiens GN=PTBP1 PE=1 SV=1
P13645	K1C10	Keratin type I cytoskeletal 10 OS=Homo sapiens GN=KRT10 PE=1 SV=6
P04264	K2C1	Keratin type II cytoskeletal 1 OS=Homo sapiens GN=KRT1 PE=1 SV=6
P35908	K22E	Keratin type II cytoskeletal 2 epidermal OS=Homo sapiens GN=KRT2 PE=1 SV=2
P35527	K1C9	Keratin type I cytoskeletal 9 OS=Homo sapiens GN=KRT9 PE=1 SV=3
P02768	ALBU	Serum albumin OS=Homo sapiens GN=ALB PE=1 SV=2
Q9UKA9	PTBP2	Polypyrimidine tract-binding protein 2 OS=Homo sapiens GN=PTBP2 PE=1 SV=1
O95758	PTBP3	Polypyrimidine tract-binding protein 3 OS=Homo sapiens GN=PTBP3 PE=1 SV=2
P13647	K2C5	Keratin type II cytoskeletal 5 OS=Homo sapiens GN=KRT5 PE=1 SV=3
P04259	K2C6B	Keratin type II cytoskeletal 6B OS=Homo sapiens GN=KRT6B PE=1 SV=5
P48668	K2C6C	Keratin type II cytoskeletal 6C OS=Homo sapiens GN=KRT6C PE=1 SV=3
P02538	K2C6A	Keratin type II cytoskeletal 6A OS=Homo sapiens GN=KRT6A PE=1 SV=3
Q8WZ42	TITIN	Titin OS=Homo sapiens GN=TTN PE=1 SV=4
P81605	DCD	Dermcidin OS=Homo sapiens GN=DCD PE=1 SV=2
Q5XKE5	K2C79	Keratin type II cytoskeletal 79 OS=Homo sapiens GN=KRT79 PE=1 SV=2
Q01546	K22O	Keratin type II cytoskeletal 2 oral OS=Homo sapiens GN=KRT76 PE=1 SV=2
O95678	K2C75	Keratin type II cytoskeletal 75 OS=Homo sapiens GN=KRT75 PE=1 SV=2
P12035	K2C3	Keratin type II cytoskeletal 3 OS=Homo sapiens GN=KRT3 PE=1 SV=3
P13646	K1C13	Keratin type I cytoskeletal 13 OS=Homo sapiens GN=KRT13 PE=1 SV=4
Q7Z3Y8	K1C27	Keratin type I cytoskeletal 27 OS=Homo sapiens GN=KRT27 PE=1 SV=2
Q2M2I5	K1C24	Keratin type I cytoskeletal 24 OS=Homo sapiens GN=KRT24 PE=1 SV=1
Q04695	K1C17	Keratin type I cytoskeletal 17 OS=Homo sapiens GN=KRT17 PE=1 SV=2
P02533	K1C14	Keratin type I cytoskeletal 14 OS=Homo sapiens GN=KRT14 PE=1 SV=4
P08727	K1C19	Keratin type I cytoskeletal 19 OS=Homo sapiens GN=KRT19 PE=1 SV=4
Q12802	AKP13	A-kinase anchor protein 13 OS=Homo sapiens GN=AKAP13 PE=1 SV=2
P19013	K2C4	Keratin type II cytoskeletal 4 OS=Homo sapiens GN=KRT4 PE=1 SV=4
P62987	RL40	Ubiquitin-60S ribosomal protein L40 OS=Homo sapiens GN=UBA52 PE=1 SV=2
P62979	RS27A	Ubiquitin-40S ribosomal protein S27a OS=Homo sapiens GN=RPS27A PE=1 SV=2
P0CG47	UBB	Polyubiquitin-B OS=Homo sapiens GN=UBB PE=1 SV=1
P0CG48	UBC	Polyubiquitin-C OS=Homo sapiens GN=UBC PE=1 SV=3
P19012	K1C15	Keratin type I cytoskeletal 15 OS=Homo sapiens GN=KRT15 PE=1 SV=3
P08779	K1C16	Keratin type I cytoskeletal 16 OS=Homo sapiens GN=KRT16 PE=1 SV=4
O76014	KRT37	Keratin type I cuticular Ha7 OS=Homo sapiens GN=KRT37 PE=3 SV=3
P05783	K1C18	Keratin type I cytoskeletal 18 OS=Homo sapiens GN=KRT18 PE=1 SV=2
Q14532	K1H2	Keratin type I cuticular Ha2 OS=Homo sapiens GN=KRT32 PE=2 SV=3
Q92764	KRT35	Keratin type I cuticular Ha5 OS=Homo sapiens GN=KRT35 PE=2 SV=5
O76013	KRT36	Keratin type I cuticular Ha6 OS=Homo sapiens GN=KRT36 PE=2 SV=1
Q9P225	DYH2	Dynein heavy chain 2 axonemal OS=Homo sapiens GN=DNAH2 PE=2 SV=3
Q8NF91	SYNE1	Nesprin-1 OS=Homo sapiens GN=SYNE1 PE=1 SV=4

P05787	K2C8	Keratin type II cytoskeletal 8 OS=Homo sapiens GN=KRT8 PE=1 SV=7
Q05639	EF1A2	Elongation factor 1-alpha 2 OS=Homo sapiens GN=EEF1A2 PE=1 SV=1
Q8WXD9	CSKI1	Caskin-1 OS=Homo sapiens GN=CASKIN1 PE=1 SV=1
Q7Z794	K2C1B	Keratin type II cytoskeletal 1b OS=Homo sapiens GN=KRT77 PE=2 SV=3
Q96HP4	OXND1	Oxidoreductase NAD-binding domain-containing protein 1 OS=Homo sapiens GN=OXNAD1 PE=1
Q96JB1	DYH8	Dynein heavy chain 8 axonemal OS=Homo sapiens GN=DNAH8 PE=1 SV=2
Q96JG9	ZN469	Zinc finger protein 469 OS=Homo sapiens GN=ZNF469 PE=2 SV=3
P55317	FOXA1	Hepatocyte nuclear factor 3-alpha OS=Homo sapiens GN=FOXA1 PE=1 SV=2
Q8NFC6	BD1L1	Biorientation of chromosomes in cell division protein 1-like 1 OS=Homo sapiens GN=BOD1L1 PE=
Q8IWN7	RP1L1	Retinitis pigmentosa 1-like 1 protein OS=Homo sapiens GN=RP1L1 PE=1 SV=4
P21817	RYR1	Ryanodine receptor 1 OS=Homo sapiens GN=RYR1 PE=1 SV=3
Q2YD98	UVSSA	UV-stimulated scaffold protein A OS=Homo sapiens GN=UVSSA PE=1 SV=2
Q7Z5J4	RAI1	Retinoic acid-induced protein 1 OS=Homo sapiens GN=RAI1 PE=1 SV=2
Q9ULH0	KDIS	Kinase D-interacting substrate of 220 kDa OS=Homo sapiens GN=KIDINS220 PE=1 SV=3
P18858	DNLI1	DNA ligase 1 OS=Homo sapiens GN=LIG1 PE=1 SV=1
Q15772	SPEG	Striated muscle preferentially expressed protein kinase OS=Homo sapiens GN=SPEG PE=1 SV=4
Q9UN73	PCDA6	Protocadherin alpha-6 OS=Homo sapiens GN=PCDHA6 PE=2 SV=1
Q6P1X5	TAF2	Transcription initiation factor TFIID subunit 2 OS=Homo sapiens GN=TAF2 PE=1 SV=3
Q86X10	RLGPB	Ral GTPase-activating protein subunit beta OS=Homo sapiens GN=RALGAPB PE=1 SV=1
Q9Y5F7	PCDGL	Protocadherin gamma-C4 OS=Homo sapiens GN=PCDHGC4 PE=2 SV=1
Q9Y5F8	PCDGJ	Protocadherin gamma-B7 OS=Homo sapiens GN=PCDHGB7 PE=2 SV=1
Q9Y5G9	PCDG4	Protocadherin gamma-A4 OS=Homo sapiens GN=PCDHGA4 PE=2 SV=1
Q9Y5G8	PCDG5	Protocadherin gamma-A5 OS=Homo sapiens GN=PCDHGA5 PE=2 SV=1
Q9Y5G0	PCDGH	Protocadherin gamma-B5 OS=Homo sapiens GN=PCDHGB5 PE=2 SV=1
Q96A65	EXOC4	Exocyst complex component 4 OS=Homo sapiens GN=EXOC4 PE=1 SV=1
Q7Z6R9	AP2D	Transcription factor AP-2-delta OS=Homo sapiens GN=TFAP2D PE=2 SV=1
O15015	ZN646	Zinc finger protein 646 OS=Homo sapiens GN=ZNF646 PE=1 SV=1
Q86YZ3	HORN	Hornerin OS=Homo sapiens GN=HRNR PE=1 SV=2
Q9HCS5	E41LA	Band 4.1-like protein 4A OS=Homo sapiens GN=EPB41L4A PE=1 SV=2
Q9BWT1	CDCA7	Cell division cycle-associated protein 7 OS=Homo sapiens GN=CDCA7 PE=1 SV=1
Q99456	K1C12	Keratin type I cytoskeletal 12 OS=Homo sapiens GN=KRT12 PE=1 SV=1
P06744	G6PI	Glucose-6-phosphate isomerase OS=Homo sapiens GN=GPI PE=1 SV=4
Q9UJV3	TRIM1	Probable E3 ubiquitin-protein ligase MID2 OS=Homo sapiens GN=MID2 PE=1 SV=3
Q02388	CO7A1	Collagen alpha-1(VII) chain OS=Homo sapiens GN=COL7A1 PE=1 SV=2
P47872	SCTR	Secretin receptor OS=Homo sapiens GN=SCTR PE=2 SV=2
Q13114	TRAF3	TNF receptor-associated factor 3 OS=Homo sapiens GN=TRAF3 PE=1 SV=2

P43652	AFAM	Afamin OS=Homo sapiens GN=AFM PE=1 SV=1
Q8NCM8	DYHC2	Cytoplasmic dynein 2 heavy chain 1 OS=Homo sapiens GN=DYNC2H1 PE=1 SV=4
P51398	RT29	28S ribosomal protein S29 mitochondrial OS=Homo sapiens GN=DAP3 PE=1 SV=1
Q07954	LRP1	Prolow-density lipoprotein receptor-related protein 1 OS=Homo sapiens GN=LRP1 PE=1 SV=2
P51530	DNA2	DNA replication ATP-dependent helicase/nuclease DNA2 OS=Homo sapiens GN=DNA2 PE=1 SV=3
Q9Y6J0	CABIN	Calcineurin-binding protein cabin-1 OS=Homo sapiens GN=CABIN1 PE=1 SV=1
Q9H1A4	APC1	Anaphase-promoting complex subunit 1 OS=Homo sapiens GN=ANAPC1 PE=1 SV=1
Q96JI7	SPTCS	Spatacsin OS=Homo sapiens GN=SPG11 PE=1 SV=3
Q96JH7	VCIP1	Deubiquitinating protein VCIP135 OS=Homo sapiens GN=VCPIP1 PE=1 SV=2
Q9BXA9	SALL3	Sal-like protein 3 OS=Homo sapiens GN=SALL3 PE=1 SV=2
Q04609	FOLH1	Glutamate carboxypeptidase 2 OS=Homo sapiens GN=FOLH1 PE=1 SV=1
Q9BYB0	SHAN3	SH3 and multiple ankyrin repeat domains protein 3 OS=Homo sapiens GN=SHANK3 PE=1 SV=3
Q9H1B7	I2BPL	Interferon regulatory factor 2-binding protein-like OS=Homo sapiens GN=IRF2BPL PE=1 SV=1
P42694	HELZ	Probable helicase with zinc finger domain OS=Homo sapiens GN=HELZ PE=1 SV=2
Q8IVF2	AHNK2	Protein AHNAK2 OS=Homo sapiens GN=AHNAK2 PE=1 SV=2
Q9NPC4	A4GAT	Lactosylceramide 4-alpha-galactosyltransferase OS=Homo sapiens GN=A4GALT PE=2 SV=1
Q06210	GFPT1	Glutamine--fructose-6-phosphate aminotransferase [isomerizing] 1 OS=Homo sapiens GN=GFPT[illegible] SV=3
P16402	H13	Histone H1.3 OS=Homo sapiens GN=HIST1H1D PE=1 SV=2
Q9H2T7	RBP17	Ran-binding protein 17 OS=Homo sapiens GN=RANBP17 PE=2 SV=1
P49757	NUMB	Protein numb homolog OS=Homo sapiens GN=NUMB PE=1 SV=2
Q9Y3P9	RBGP1	Rab GTPase-activating protein 1 OS=Homo sapiens GN=RABGAP1 PE=1 SV=3
A5PL33	KRBA1	Protein KRBA1 OS=Homo sapiens GN=KRBA1 PE=1 SV=3
Q6SPF0	SAMD1	Atherin OS=Homo sapiens GN=SAMD1 PE=1 SV=1
Q6Y7W6	PERQ2	PERQ amino acid-rich with GYF domain-containing protein 2 OS=Homo sapiens GN=GIGYF2 PE=1[illegible]
Q9NPF5	DMAP1	DNA methyltransferase 1-associated protein 1 OS=Homo sapiens GN=DMAP1 PE=1 SV=1
Q9NSE4	SYIM	Isoleucine--tRNA ligase mitochondrial OS=Homo sapiens GN=IARS2 PE=1 SV=2
Q8N612	F16A2	FTS and Hook-interacting protein OS=Homo sapiens GN=FAM160A2 PE=1 SV=3
Q8TEJ3	SH3R3	SH3 domain-containing RING finger protein 3 OS=Homo sapiens GN=SH3RF3 PE=1 SV=2
Q9Y5Z7	HCFC2	Host cell factor 2 OS=Homo sapiens GN=HCFC2 PE=1 SV=1
P13591	NCAM1	Neural cell adhesion molecule 1 OS=Homo sapiens GN=NCAM1 PE=1 SV=3
Q5VWQ0	RSBN1	Round spermatid basic protein 1 OS=Homo sapiens GN=RSBN1 PE=1 SV=2
P55196	AFAD	Afadin OS=Homo sapiens GN=MLLT4 PE=1 SV=3
Q9Y5H6	PCDA8	Protocadherin alpha-8 OS=Homo sapiens GN=PCDHA8 PE=2 SV=1
Q9Y5I1	PCDAB	Protocadherin alpha-11 OS=Homo sapiens GN=PCDHA11 PE=2 SV=1
Q00987	MDM2	E3 ubiquitin-protein ligase Mdm2 OS=Homo sapiens GN=MDM2 PE=1 SV=1
