# Supplementary material for: Polypyrimidine tract binding proteins PTBP1 and PTBP2 interact with distinct proteins under splicing conditions
Source: PLoS One. 2022 Feb 3;17(2):e0263287. doi: 10.1371/journal.pone.0263287 (PMC8812845; doi:10.1371/journal.pone.0263287)
Supplement: S2 Table — A list of proteins in HeLa nuclear extract that unspecifically bind to the Ni-NTA beads. (PDF) [file pone.0263287.s002.pdf]

UniProtID	Gene	Description
Q09666	AHNK	Neuroblast differentiation-associated protein AHNAK OS=Homo sapiens GN=AHNAK PE=1
Q13813	SPTN1	Spectrin alpha chain non-erythrocytic 1 OS=Homo sapiens GN=SPTAN1 PE=1 SV=3
Q01082	SPTB2	Spectrin beta chain non-erythrocytic 1 OS=Homo sapiens GN=SPTBN1 PE=1 SV=2
P21333	FLNA	Filamin-A OS=Homo sapiens GN=FLNA PE=1 SV=4
P02545	LMNA	Prelamin-A/C OS=Homo sapiens GN=LMNA PE=1 SV=1
P11142	HSP7C	Heat shock cognate 71 kDa protein OS=Homo sapiens GN=HSPA8 PE=1 SV=1
P07355	ANXA2	Annexin A2 OS=Homo sapiens GN=ANXA2 PE=1 SV=2
P0DMV8	HS71A	Heat shock 70 kDa protein 1A OS=Homo sapiens GN=HSPA1A PE=1 SV=1
P0DMV9	HS71B	Heat shock 70 kDa protein 1B OS=Homo sapiens GN=HSPA1B PE=1 SV=1
P08238	HS90B	Heat shock protein HSP 90-beta OS=Homo sapiens GN=HSP90AB1 PE=1 SV=4
P63261	ACTG	Actin cytoplasmic 2 OS=Homo sapiens GN=ACTG1 PE=1 SV=1
P60709	ACTB	Actin cytoplasmic 1 OS=Homo sapiens GN=ACTB PE=1 SV=1
P68104	EF1A1	Elongation factor 1-alpha 1 OS=Homo sapiens GN=EEF1A1 PE=1 SV=1
P07900	HS90A	Heat shock protein HSP 90-alpha OS=Homo sapiens GN=HSP90AA1 PE=1 SV=5
Q5VTE0	EF1A3	Putative elongation factor 1-alpha-like 3 OS=Homo sapiens GN=EEF1A1P5 PE=5 SV=1
O75369	FLNB	Filamin-B OS=Homo sapiens GN=FLNB PE=1 SV=2
Q15393	SF3B3	Splicing factor 3B subunit 3 OS=Homo sapiens GN=SF3B3 PE=1 SV=4
Q16531	DDB1	DNA damage-binding protein 1 OS=Homo sapiens GN=DDB1 PE=1 SV=1
O43143	DHX15	Pre-mRNA-splicing factor ATP-dependent RNA helicase DHX15 OS=Homo sapiens GN=DH
P54652	HSP72	Heat shock-related 70 kDa protein 2 OS=Homo sapiens GN=HSPA2 PE=1 SV=1
Q05639	EF1A2	Elongation factor 1-alpha 2 OS=Homo sapiens GN=EEF1A2 PE=1 SV=1
P34931	HS71L	Heat shock 70 kDa protein 1-like OS=Homo sapiens GN=HSPA1L PE=1 SV=2
Q96AE4	FUBP1	Far upstream element-binding protein 1 OS=Homo sapiens GN=FUBP1 PE=1 SV=3
Q9UHX1	PUF60	Poly(U)-binding-splicing factor PUF60 OS=Homo sapiens GN=PUF60 PE=1 SV=1
E9PAV3	NACAM	Nascent polypeptide-associated complex subunit alpha muscle-specific form OS=Homo s
Q15459	SF3A1	Splicing factor 3A subunit 1 OS=Homo sapiens GN=SF3A1 PE=1 SV=1
Q9P258	RCC2	Protein RCC2 OS=Homo sapiens GN=RCC2 PE=1 SV=2
O60506	HNRPQ	Heterogeneous nuclear ribonucleoprotein Q OS=Homo sapiens GN=SYNCRIP PE=1 SV=2
P26358	DNMT1	DNA (cytosine-5)-methyltransferase 1 OS=Homo sapiens GN=DNMT1 PE=1 SV=2
P51610	HCFC1	Host cell factor 1 OS=Homo sapiens GN=HCFC1 PE=1 SV=2
P63241	IF5A1	Eukaryotic translation initiation factor 5A-1 OS=Homo sapiens GN=EIF5A PE=1 SV=2
P09651	ROA1	Heterogeneous nuclear ribonucleoprotein A1 OS=Homo sapiens GN=HNRNPA1 PE=1 SV=
Q00839	HNRPU	Heterogeneous nuclear ribonucleoprotein U OS=Homo sapiens GN=HNRNPU PE=1 SV=6
O43707	ACTN4	Alpha-actinin-4 OS=Homo sapiens GN=ACTN4 PE=1 SV=2
Q58FF7	H90B3	Putative heat shock protein HSP 90-beta-3 OS=Homo sapiens GN=HSP90AB3P PE=5 SV=1
Q86XP3	DDX42	ATP-dependent RNA helicase DDX42 OS=Homo sapiens GN=DDX42 PE=1 SV=1
P68032	ACTC	Actin alpha cardiac muscle 1 OS=Homo sapiens GN=ACTC1 PE=1 SV=1
P68133	ACTS	Actin alpha skeletal muscle OS=Homo sapiens GN=ACTA1 PE=1 SV=1
O43390	HNRPR	Heterogeneous nuclear ribonucleoprotein R OS=Homo sapiens GN=HNRNPR PE=1 SV=1
Q16630	CPSF6	Cleavage and polyadenylation specificity factor subunit 6 OS=Homo sapiens GN=CPSF6 PE

Q6P2Q9	PRP8	Pre-mRNA-processing-splicing factor 8 OS=Homo sapiens GN=PRPF8 PE=1 SV=2
P61978	HNRPK	Heterogeneous nuclear ribonucleoprotein K OS=Homo sapiens GN=HNRNPK PE=1 SV=1
P22626	ROA2	Heterogeneous nuclear ribonucleoproteins A2/B1 OS=Homo sapiens GN=HNRNPA2B1 PE
Q6S8J3	POTEE	POTE ankyrin domain family member E OS=Homo sapiens GN=POTEE PE=1 SV=3
P17066	HSP76	Heat shock 70 kDa protein 6 OS=Homo sapiens GN=HSPA6 PE=1 SV=2
Q92922	SMRC1	SWI/SNF complex subunit SMARCC1 OS=Homo sapiens GN=SMARCC1 PE=1 SV=3
Q13435	SF3B2	Splicing factor 3B subunit 2 OS=Homo sapiens GN=SF3B2 PE=1 SV=2
P62736	ACTA	Actin aortic smooth muscle OS=Homo sapiens GN=ACTA2 PE=1 SV=1
P63267	ACTH	Actin gamma-enteric smooth muscle OS=Homo sapiens GN=ACTG2 PE=1 SV=1
P0CG47	UBB	Polyubiquitin-B OS=Homo sapiens GN=UBB PE=1 SV=1
P0CG48	UBC	Polyubiquitin-C OS=Homo sapiens GN=UBC PE=1 SV=3
A5A3E0	POTEF	POTE ankyrin domain family member F OS=Homo sapiens GN=POTEF PE=1 SV=2
P55072	TERA	Transitional endoplasmic reticulum ATPase OS=Homo sapiens GN=VCP PE=1 SV=4
Q32P51	RA1L2	Heterogeneous nuclear ribonucleoprotein A1-like 2 OS=Homo sapiens GN=HNRNPA1L2 P
P26583	HMGB2	High mobility group protein B2 OS=Homo sapiens GN=HMGB2 PE=1 SV=2
Q15029	U5S1	116 kDa U5 small nuclear ribonucleoprotein component OS=Homo sapiens GN=EFTUD2 P
Q6IS14	IF5AL	Eukaryotic translation initiation factor 5A-1-like OS=Homo sapiens GN=EIF5AL1 PE=2 SV=
O75643	U520	U5 small nuclear ribonucleoprotein 200 kDa helicase OS=Homo sapiens GN=SNRNP200 PE
P18754	RCC1	Regulator of chromosome condensation OS=Homo sapiens GN=RCC1 PE=1 SV=1
Q92979	NEP1	Ribosomal RNA small subunit methyltransferase NEP1 OS=Homo sapiens GN=EMG1 PE=1
P31943	HNRH1	Heterogeneous nuclear ribonucleoprotein H OS=Homo sapiens GN=HNRNPH1 PE=1 SV=4
P09874	PARP1	Poly [ADP-ribose] polymerase 1 OS=Homo sapiens GN=PARP1 PE=1 SV=4
Q8N684	CPSF7	Cleavage and polyadenylation specificity factor subunit 7 OS=Homo sapiens GN=CPSF7 PE
P23246	SFPQ	Splicing factor proline- and glutamine-rich OS=Homo sapiens GN=SFPQ PE=1 SV=2
P78347	GTF2I	General transcription factor II-I OS=Homo sapiens GN=GTF2I PE=1 SV=2
P12270	TPR	Nucleoprotein TPR OS=Homo sapiens GN=TPR PE=1 SV=3
P09429	HMGB1	High mobility group protein B1 OS=Homo sapiens GN=HMGB1 PE=1 SV=3
P27816	MAP4	Microtubule-associated protein 4 OS=Homo sapiens GN=MAP4 PE=1 SV=3
Q05519	SRS11	Serine/arginine-rich splicing factor 11 OS=Homo sapiens GN=SRSF11 PE=1 SV=1
P11021	GRP78	78 kDa glucose-regulated protein OS=Homo sapiens GN=HSPA5 PE=1 SV=2
P07437	TBB5	Tubulin beta chain OS=Homo sapiens GN=TUBB PE=1 SV=2
P08621	RU17	U1 small nuclear ribonucleoprotein 70 kDa OS=Homo sapiens GN=SNRNP70 PE=1 SV=2
Q99460	PSMD1	26S proteasome non-ATPase regulatory subunit 1 OS=Homo sapiens GN=PSMD1 PE=1 SV
Q8TAQ2	SMRC2	SWI/SNF complex subunit SMARCC2 OS=Homo sapiens GN=SMARCC2 PE=1 SV=1
P26599	PTBP1	Polypyrimidine tract-binding protein 1 OS=Homo sapiens GN=PTBP1 PE=1 SV=1
tr	A0A087WWQ2	Uncharacterized protein (Fragment) OS=Homo sapiens PE=4 SV=1
Q9BYX7	ACTBM	Putative beta-actin-like protein 3 OS=Homo sapiens GN=POTEKP PE=5 SV=1
O43242	PSMD3	26S proteasome non-ATPase regulatory subunit 3 OS=Homo sapiens GN=PSMD3 PE=1 SV
P61956	SUMO2	Small ubiquitin-related modifier 2 OS=Homo sapiens GN=SUMO2 PE=1 SV=3
Q9Y383	LC7L2	Putative RNA-binding protein Luc7-like 2 OS=Homo sapiens GN=LUC7L2 PE=1 SV=2
P09012	SNRPA	U1 small nuclear ribonucleoprotein A OS=Homo sapiens GN=SNRPA PE=1 SV=3

P48741	HSP77	Putative heat shock 70 kDa protein 7 OS=Homo sapiens GN=HSPA7 PE=5 SV=2
Q9BQE3	TBA1C	Tubulin alpha-1C chain OS=Homo sapiens GN=TUBA1C PE=1 SV=1
P68363	TBA1B	Tubulin alpha-1B chain OS=Homo sapiens GN=TUBA1B PE=1 SV=1
P55854	SUMO3	Small ubiquitin-related modifier 3 OS=Homo sapiens GN=SUMO3 PE=1 SV=2
Q71U36	TBA1A	Tubulin alpha-1A chain OS=Homo sapiens GN=TUBA1A PE=1 SV=1
Q58FF8	H90B2	Putative heat shock protein HSP 90-beta 2 OS=Homo sapiens GN=HSP90AB2P PE=1 SV=2
O43809	CPSF5	Cleavage and polyadenylation specificity factor subunit 5 OS=Homo sapiens GN=NUDT21
Q969G3	SMCE1	SWI/SNF-related matrix-associated actin-dependent regulator of chromatin subfamily E m SV=2
P26641	EF1G	Elongation factor 1-gamma OS=Homo sapiens GN=EEF1G PE=1 SV=3
P62258	1433E	14-3-3 protein epsilon OS=Homo sapiens GN=YWHAE PE=1 SV=1
P0CG38	POTEI	POTE ankyrin domain family member I OS=Homo sapiens GN=POTEI PE=3 SV=1
P68371	TBB4B	Tubulin beta-4B chain OS=Homo sapiens GN=TUBB4B PE=1 SV=1
Q14683	SMC1A	Structural maintenance of chromosomes protein 1A OS=Homo sapiens GN=SMC1A PE=1 S
P04264	K2C1	Keratin type II cytoskeletal 1 OS=Homo sapiens GN=KRT1 PE=1 SV=6
Q9Y265	RUVB1	RuvB-like 1 OS=Homo sapiens GN=RUVBL1 PE=1 SV=1
Q13561	DCTN2	Dynactin subunit 2 OS=Homo sapiens GN=DCTN2 PE=1 SV=4
Q7L014	DDX46	Probable ATP-dependent RNA helicase DDX46 OS=Homo sapiens GN=DDX46 PE=1 SV=2
Q07666	KHDR1	KH domain-containing RNA-binding signal transduction-associated protein 1 OS=Homo s
Q13442	HAP28	28 kDa heat- and acid-stable phosphoprotein OS=Homo sapiens GN=PDAP1 PE=1 SV=1
Q12906	ILF3	Interleukin enhancer-binding factor 3 OS=Homo sapiens GN=ILF3 PE=1 SV=3
Q8TF74	WIPF2	WAS/WASL-interacting protein family member 2 OS=Homo sapiens GN=WIPF2 PE=1 SV=1
P11940	PABP1	Polyadenylate-binding protein 1 OS=Homo sapiens GN=PABPC1 PE=1 SV=2
P06753	TPM3	Tropomyosin alpha-3 chain OS=Homo sapiens GN=TPM3 PE=1 SV=2
Q8WUA2	PPIL4	Peptidyl-prolyl cis-trans isomerase-like 4 OS=Homo sapiens GN=PPIL4 PE=1 SV=1
Q15084	PDIA6	Protein disulfide-isomerase A6 OS=Homo sapiens GN=PDIA6 PE=1 SV=1
Q09028	RBBP4	Histone-binding protein RBBP4 OS=Homo sapiens GN=RBBP4 PE=1 SV=3
P14866	HNRPL	Heterogeneous nuclear ribonucleoprotein L OS=Homo sapiens GN=HNRNPL PE=1 SV=2
P20810	ICAL	Calpastatin OS=Homo sapiens GN=CAST PE=1 SV=4
Q9UQE7	SMC3	Structural maintenance of chromosomes protein 3 OS=Homo sapiens GN=SMC3 PE=1 SV=
Q562R1	ACTBL	Beta-actin-like protein 2 OS=Homo sapiens GN=ACTBL2 PE=1 SV=2
P46940	IQGA1	Ras GTPase-activating-like protein IQGAP1 OS=Homo sapiens GN=IQGAP1 PE=1 SV=1
P60900	PSA6	Proteasome subunit alpha type-6 OS=Homo sapiens GN=PSMA6 PE=1 SV=1
P12956	XRCC6	X-ray repair cross-complementing protein 6 OS=Homo sapiens GN=XRCC6 PE=1 SV=2
O95817	BAG3	BAG family molecular chaperone regulator 3 OS=Homo sapiens GN=BAG3 PE=1 SV=3
P28074	PSB5	Proteasome subunit beta type-5 OS=Homo sapiens GN=PSMB5 PE=1 SV=3
O94776	MTA2	Metastasis-associated protein MTA2 OS=Homo sapiens GN=MTA2 PE=1 SV=1
Q96GM5	SMRD1	SWI/SNF-related matrix-associated actin-dependent regulator of chromatin subfamily D m SV=2
Q9BUI4	RPC3	DNA-directed RNA polymerase III subunit RPC3 OS=Homo sapiens GN=POLR3C PE=1 SV=1
P04350	TBB4A	Tubulin beta-4A chain OS=Homo sapiens GN=TUBB4A PE=1 SV=2
Q9ULV4	COR1C	Coronin-1C OS=Homo sapiens GN=CORO1C PE=1 SV=1

O60664	PLIN3	Perilipin-3 OS=Homo sapiens GN=PLIN3 PE=1 SV=3
O75534	CSDE1	Cold shock domain-containing protein E1 OS=Homo sapiens GN=CSDE1 PE=1 SV=2
P62191	PRS4	26S protease regulatory subunit 4 OS=Homo sapiens GN=PSMC1 PE=1 SV=1
Q9UMS4	PRP19	Pre-mRNA-processing factor 19 OS=Homo sapiens GN=PRPF19 PE=1 SV=1
Q8WXA9	SREK1	Splicing regulatory glutamine/lysine-rich protein 1 OS=Homo sapiens GN=SREK1 PE=1 SV=
O60341	KDM1A	Lysine-specific histone demethylase 1A OS=Homo sapiens GN=KDM1A PE=1 SV=2
O00267	SPT5H	Transcription elongation factor SPT5 OS=Homo sapiens GN=SUPT5H PE=1 SV=1
Q9UKL0	RCOR1	REST corepressor 1 OS=Homo sapiens GN=RCOR1 PE=1 SV=1
P25788	PSA3	Proteasome subunit alpha type-3 OS=Homo sapiens GN=PSMA3 PE=1 SV=2
Q13573	SNW1	SNW domain-containing protein 1 OS=Homo sapiens GN=SNW1 PE=1 SV=1
Q8NFC6	BD1L1	Biorientation of chromosomes in cell division protein 1-like 1 OS=Homo sapiens GN=BOD
Q16576	RBBP7	Histone-binding protein RBBP7 OS=Homo sapiens GN=RBBP7 PE=1 SV=1
Q9Y3F4	STRAP	Serine-threonine kinase receptor-associated protein OS=Homo sapiens GN=STRAP PE=1 S
P42285	SK2L2	Superkiller viralicidic activity 2-like 2 OS=Homo sapiens GN=SKIV2L2 PE=1 SV=3
P52597	HNRPF	Heterogeneous nuclear ribonucleoprotein F OS=Homo sapiens GN=HNRNPF PE=1 SV=3
P62314	SMD1	Small nuclear ribonucleoprotein Sm D1 OS=Homo sapiens GN=SNRPD1 PE=1 SV=1
P55795	HNRH2	Heterogeneous nuclear ribonucleoprotein H2 OS=Homo sapiens GN=HNRNPH2 PE=1 SV=
P02768	ALBU	Serum albumin OS=Homo sapiens GN=ALB PE=1 SV=2
P51665	PSMD7	26S proteasome non-ATPase regulatory subunit 7 OS=Homo sapiens GN=PSMD7 PE=1 SV
P26368	U2AF2	Splicing factor U2AF 65 kDa subunit OS=Homo sapiens GN=U2AF2 PE=1 SV=4
P30533	AMRP	Alpha-2-macroglobulin receptor-associated protein OS=Homo sapiens GN=LRPAP1 PE=1 S
Q13283	G3BP1	Ras GTPase-activating protein-binding protein 1 OS=Homo sapiens GN=G3BP1 PE=1 SV=1
Q12905	ILF2	Interleukin enhancer-binding factor 2 OS=Homo sapiens GN=ILF2 PE=1 SV=2
P29692	EF1D	Elongation factor 1-delta OS=Homo sapiens GN=EEF1D PE=1 SV=5
Q92466	DDB2	DNA damage-binding protein 2 OS=Homo sapiens GN=DDB2 PE=1 SV=1
P67936	TPM4	Tropomyosin alpha-4 chain OS=Homo sapiens GN=TPM4 PE=1 SV=3
Q93009	UBP7	Ubiquitin carboxyl-terminal hydrolase 7 OS=Homo sapiens GN=USP7 PE=1 SV=2
O14818	PSA7	Proteasome subunit alpha type-7 OS=Homo sapiens GN=PSMA7 PE=1 SV=1
P62316	SMD2	Small nuclear ribonucleoprotein Sm D2 OS=Homo sapiens GN=SNRPD2 PE=1 SV=1
Q8TAT6	NPL4	Nuclear protein localization protein 4 homolog OS=Homo sapiens GN=NPLOC4 PE=1 SV=3
P63104	1433Z	14-3-3 protein zeta/delta OS=Homo sapiens GN=YWHAZ PE=1 SV=1
Q92945	FUBP2	Far upstream element-binding protein 2 OS=Homo sapiens GN=KHSRP PE=1 SV=4
Q16204	CCDC6	Coiled-coil domain-containing protein 6 OS=Homo sapiens GN=CCDC6 PE=1 SV=2
Q13310	PABP4	Polyadenylate-binding protein 4 OS=Homo sapiens GN=PABPC4 PE=1 SV=1
P35908	K22E	Keratin type II cytoskeletal 2 epidermal OS=Homo sapiens GN=KRT2 PE=1 SV=2
P52907	CAZA1	F-actin-capping protein subunit alpha-1 OS=Homo sapiens GN=CAPZA1 PE=1 SV=3
P68366	TBA4A	Tubulin alpha-4A chain OS=Homo sapiens GN=TUBA4A PE=1 SV=1
P06748	NPM	Nucleophosmin OS=Homo sapiens GN=NPM1 PE=1 SV=2
Q15365	PCBP1	Poly(rC)-binding protein 1 OS=Homo sapiens GN=PCBP1 PE=1 SV=2
P61964	WDR5	WD repeat-containing protein 5 OS=Homo sapiens GN=WDR5 PE=1 SV=1
O43684	BUB3	Mitotic checkpoint protein BUB3 OS=Homo sapiens GN=BUB3 PE=1 SV=1

Q8WX93	PALLD	Palladin OS=Homo sapiens GN=PALLD PE=1 SV=3
Q14247	SRC8	Src substrate cortactin OS=Homo sapiens GN=CTTN PE=1 SV=2
Q9NTZ6	RBM12	RNA-binding protein 12 OS=Homo sapiens GN=RBM12 PE=1 SV=1
O60828	PQBP1	Polyglutamine-binding protein 1 OS=Homo sapiens GN=PQBP1 PE=1 SV=1
P35998	PRS7	26S protease regulatory subunit 7 OS=Homo sapiens GN=PSMC2 PE=1 SV=3
P37108	SRP14	Signal recognition particle 14 kDa protein OS=Homo sapiens GN=SRP14 PE=1 SV=2
O00487	PSDE	26S proteasome non-ATPase regulatory subunit 14 OS=Homo sapiens GN=PSMD14 PE=1
O95347	SMC2	Structural maintenance of chromosomes protein 2 OS=Homo sapiens GN=SMC2 PE=1 SV
P35527	K1C9	Keratin type I cytoskeletal 9 OS=Homo sapiens GN=KRT9 PE=1 SV=3
P13645	K1C10	Keratin type I cytoskeletal 10 OS=Homo sapiens GN=KRT10 PE=1 SV=6
P60660	MYL6	Myosin light polypeptide 6 OS=Homo sapiens GN=MYL6 PE=1 SV=2
P25787	PSA2	Proteasome subunit alpha type-2 OS=Homo sapiens GN=PSMA2 PE=1 SV=2
Q15233	NONO	Non-POU domain-containing octamer-binding protein OS=Homo sapiens GN=NONO PE=1
P31942	HNRH3	Heterogeneous nuclear ribonucleoprotein H3 OS=Homo sapiens GN=HNRNPH3 PE=1 SV=
Q12874	SF3A3	Splicing factor 3A subunit 3 OS=Homo sapiens GN=SF3A3 PE=1 SV=1
P55036	PSMD4	26S proteasome non-ATPase regulatory subunit 4 OS=Homo sapiens GN=PSMD4 PE=1 SV
Q01130	SRSF2	Serine/arginine-rich splicing factor 2 OS=Homo sapiens GN=SRSF2 PE=1 SV=4
P35637	FUS	RNA-binding protein FUS OS=Homo sapiens GN=FUS PE=1 SV=1
P07951	TPM2	Tropomyosin beta chain OS=Homo sapiens GN=TPM2 PE=1 SV=1
P0CG39	POTEJ	POTE ankyrin domain family member J OS=Homo sapiens GN=POTEJ PE=3 SV=1
P43686	PRS6B	26S protease regulatory subunit 6B OS=Homo sapiens GN=PSMC4 PE=1 SV=2
Q9Y3I0	RTCB	tRNA-splicing ligase RtcB homolog OS=Homo sapiens GN=RTCB PE=1 SV=1
Q15046	SYK	Lysine--tRNA ligase OS=Homo sapiens GN=KARS PE=1 SV=3
O75533	SF3B1	Splicing factor 3B subunit 1 OS=Homo sapiens GN=SF3B1 PE=1 SV=3
Q9H361	PABP3	Polyadenylate-binding protein 3 OS=Homo sapiens GN=PABPC3 PE=1 SV=2
P30041	PRDX6	Peroxiredoxin-6 OS=Homo sapiens GN=PRDX6 PE=1 SV=3
Q86V81	THOC4	THO complex subunit 4 OS=Homo sapiens GN=ALYREF PE=1 SV=3
O00231	PSD11	26S proteasome non-ATPase regulatory subunit 11 OS=Homo sapiens GN=PSMD11 PE=1
Q8TAA3	PSA7L	Proteasome subunit alpha type-7-like OS=Homo sapiens GN=PSMA8 PE=2 SV=3
P31948	STIP1	Stress-induced-phosphoprotein 1 OS=Homo sapiens GN=STIP1 PE=1 SV=1
Q9NUQ3	TXLNG	Gamma-taxilin OS=Homo sapiens GN=TXLNG PE=1 SV=2
P31689	DNJA1	DnaJ homolog subfamily A member 1 OS=Homo sapiens GN=DNAJA1 PE=1 SV=2
Q9Y230	RUVB2	RuvB-like 2 OS=Homo sapiens GN=RUVBL2 PE=1 SV=3
P53999	TCP4	Activated RNA polymerase II transcriptional coactivator p15 OS=Homo sapiens GN=SUB1
Q13263	TIF1B	Transcription intermediary factor 1-beta OS=Homo sapiens GN=TRIM28 PE=1 SV=5
Q9BZK3	NACP1	Putative nascent polypeptide-associated complex subunit alpha-like protein OS=Homo sa
Q15427	SF3B4	Splicing factor 3B subunit 4 OS=Homo sapiens GN=SF3B4 PE=1 SV=1
Q15008	PSMD6	26S proteasome non-ATPase regulatory subunit 6 OS=Homo sapiens GN=PSMD6 PE=1 SV
P20618	PSB1	Proteasome subunit beta type-1 OS=Homo sapiens GN=PSMB1 PE=1 SV=2
P62826	RAN	GTP-binding nuclear protein Ran OS=Homo sapiens GN=RAN PE=1 SV=3
Q8IVF2	AHNK2	Protein AHNAK2 OS=Homo sapiens GN=AHNAK2 PE=1 SV=2

Q00610	CLH1	Clathrin heavy chain 1 OS=Homo sapiens GN=CLTC PE=1 SV=5
P62333	PRS10	26S protease regulatory subunit 10B OS=Homo sapiens GN=PSMC6 PE=1 SV=1
Q4G0J3	LARP7	La-related protein 7 OS=Homo sapiens GN=LARP7 PE=1 SV=1
P55209	NP1L1	Nucleosome assembly protein 1-like 1 OS=Homo sapiens GN=NAP1L1 PE=1 SV=1
P14678	RSMB	Small nuclear ribonucleoprotein-associated proteins B and B' OS=Homo sapiens GN=SNRI
Q9NZ63	CI078	Uncharacterized protein C9orf78 OS=Homo sapiens GN=C9orf78 PE=1 SV=1
O60306	AQR	Intron-binding protein aquarius OS=Homo sapiens GN=AQR PE=1 SV=4
Q96B97	SH3K1	SH3 domain-containing kinase-binding protein 1 OS=Homo sapiens GN=SH3KBP1 PE=1 SV
P60903	S10AA	Protein S100-A10 OS=Homo sapiens GN=S100A10 PE=1 SV=2
Q15404	RSU1	Ras suppressor protein 1 OS=Homo sapiens GN=RSU1 PE=1 SV=3
P08579	RU2B	U2 small nuclear ribonucleoprotein B'' OS=Homo sapiens GN=SNRPB2 PE=1 SV=1
Q14315	FLNC	Filamin-C OS=Homo sapiens GN=FLNC PE=1 SV=3
O15318	RPC7	DNA-directed RNA polymerase III subunit RPC7 OS=Homo sapiens GN=POLR3G PE=1 SV=
Q08945	SSRP1	FACT complex subunit SSRP1 OS=Homo sapiens GN=SSRP1 PE=1 SV=1
Q99961	SH3G1	Endophilin-A2 OS=Homo sapiens GN=SH3GL1 PE=1 SV=1
Q92499	DDX1	ATP-dependent RNA helicase DDX1 OS=Homo sapiens GN=DDX1 PE=1 SV=2
Q96IZ0	PAWR	PRKC apoptosis WT1 regulator protein OS=Homo sapiens GN=PAWR PE=1 SV=1
O14776	TCRG1	Transcription elongation regulator 1 OS=Homo sapiens GN=TCERG1 PE=1 SV=2
Q9NQ29	LUC7L	Putative RNA-binding protein Luc7-like 1 OS=Homo sapiens GN=LUC7L PE=1 SV=1
Q7Z5L9	I2BP2	Interferon regulatory factor 2-binding protein 2 OS=Homo sapiens GN=IRF2BP2 PE=1 SV=
Q9UNP9	PPIE	Peptidyl-prolyl cis-trans isomerase E OS=Homo sapiens GN=PPIE PE=1 SV=1
Q01970	PLCB3	1-phosphatidylinositol 4 5-bisphosphate phosphodiesterase beta-3 OS=Homo sapiens GN
Q13200	PSMD2	26S proteasome non-ATPase regulatory subunit 2 OS=Homo sapiens GN=PSMD2 PE=1 SV
Q99733	NP1L4	Nucleosome assembly protein 1-like 4 OS=Homo sapiens GN=NAP1L4 PE=1 SV=1
P04259	K2C6B	Keratin type II cytoskeletal 6B OS=Homo sapiens GN=KRT6B PE=1 SV=5
P19784	CSK22	Casein kinase II subunit alpha' OS=Homo sapiens GN=CSNK2A2 PE=1 SV=1
A2A3N6	PIPSL	Putative PIP5K1A and PSMD4-like protein OS=Homo sapiens GN=PIPSL PE=5 SV=1
P17980	PRS6A	26S protease regulatory subunit 6A OS=Homo sapiens GN=PSMC3 PE=1 SV=3
Q00341	VIGLN	Vigilin OS=Homo sapiens GN=HDLBP PE=1 SV=2
Q15717	ELAV1	ELAV-like protein 1 OS=Homo sapiens GN=ELAVL1 PE=1 SV=2
Q9H910	HN1L	Hematological and neurological expressed 1-like protein OS=Homo sapiens GN=HN1L PE=
P61088	UBE2N	Ubiquitin-conjugating enzyme E2 N OS=Homo sapiens GN=UBE2N PE=1 SV=1
O60231	DHX16	Putative pre-mRNA-splicing factor ATP-dependent RNA helicase DHX16 OS=Homo sapien
O95104	SFR15	Splicing factor arginine/serine-rich 15 OS=Homo sapiens GN=SCAF4 PE=1 SV=3
P61163	ACTZ	Alpha-centractin OS=Homo sapiens GN=ACTR1A PE=1 SV=1
Q99729	ROAA	Heterogeneous nuclear ribonucleoprotein A/B OS=Homo sapiens GN=HNRNPAB PE=1 SV=
Q8N1G4	LRC47	Leucine-rich repeat-containing protein 47 OS=Homo sapiens GN=LRRC47 PE=1 SV=1
O15020	SPTN2	Spectrin beta chain non-erythrocytic 2 OS=Homo sapiens GN=SPTBN2 PE=1 SV=3
O75150	BRE1B	E3 ubiquitin-protein ligase BRE1B OS=Homo sapiens GN=RNF40 PE=1 SV=4
Q68EM7	RHG17	Rho GTPase-activating protein 17 OS=Homo sapiens GN=ARHGAP17 PE=1 SV=1
Q14103	HNRPD	Heterogeneous nuclear ribonucleoprotein D0 OS=Homo sapiens GN=HNRNPD PE=1 SV=1

P25205	MCM3	DNA replication licensing factor MCM3 OS=Homo sapiens GN=MCM3 PE=1 SV=3
Q58FG1	HS904	Putative heat shock protein HSP 90-alpha A4 OS=Homo sapiens GN=HSP90AA4P PE=5 SV=
Q15785	TOM34	Mitochondrial import receptor subunit TOM34 OS=Homo sapiens GN=TOMM34 PE=1 SV=
P27694	RFA1	Replication protein A 70 kDa DNA-binding subunit OS=Homo sapiens GN=RPA1 PE=1 SV=2
P62318	SMD3	Small nuclear ribonucleoprotein Sm D3 OS=Homo sapiens GN=SNRPD3 PE=1 SV=1
P98179	RBM3	RNA-binding protein 3 OS=Homo sapiens GN=RBM3 PE=1 SV=1
P78346	RPP30	Ribonuclease P protein subunit p30 OS=Homo sapiens GN=RPP30 PE=1 SV=1
O60493	SNX3	Sorting nexin-3 OS=Homo sapiens GN=SNX3 PE=1 SV=3
Q99614	TTC1	Tetratricopeptide repeat protein 1 OS=Homo sapiens GN=TTC1 PE=1 SV=1
P62158	CALM	Calmodulin OS=Homo sapiens GN=CALM1 PE=1 SV=2
P28066	PSA5	Proteasome subunit alpha type-5 OS=Homo sapiens GN=PSMA5 PE=1 SV=3
O95983	MBD3	Methyl-CpG-binding domain protein 3 OS=Homo sapiens GN=MBD3 PE=1 SV=1
P51532	SMCA4	Transcription activator BRG1 OS=Homo sapiens GN=SMARCA4 PE=1 SV=2
O00232	PSD12	26S proteasome non-ATPase regulatory subunit 12 OS=Homo sapiens GN=PSMD12 PE=1
Q13868	EXOS2	Exosome complex component RRP4 OS=Homo sapiens GN=EXOSC2 PE=1 SV=2
O96019	ACL6A	Actin-like protein 6A OS=Homo sapiens GN=ACTL6A PE=1 SV=1
P40222	TXLNA	Alpha-taxilin OS=Homo sapiens GN=TXLNA PE=1 SV=3
O43399	TPD54	Tumor protein D54 OS=Homo sapiens GN=TPD52L2 PE=1 SV=2
Q9UK59	DBR1	Lariat debranching enzyme OS=Homo sapiens GN=DBR1 PE=1 SV=2
P05198	IF2A	Eukaryotic translation initiation factor 2 subunit 1 OS=Homo sapiens GN=EIF2S1 PE=1 SV=
Q07955	SRSF1	Serine/arginine-rich splicing factor 1 OS=Homo sapiens GN=SRSF1 PE=1 SV=2
Q96CT7	CC124	Coiled-coil domain-containing protein 124 OS=Homo sapiens GN=CCDC124 PE=1 SV=1
Q92890	UFD1	Ubiquitin fusion degradation protein 1 homolog OS=Homo sapiens GN=UFD1L PE=1 SV=3
O43148	MCES	mRNA cap guanine-N7 methyltransferase OS=Homo sapiens GN=RNMT PE=1 SV=1
P84103	SRSF3	Serine/arginine-rich splicing factor 3 OS=Homo sapiens GN=SRSF3 PE=1 SV=1
Q13185	CBX3	Chromobox protein homolog 3 OS=Homo sapiens GN=CBX3 PE=1 SV=4
P56545	CTBP2	C-terminal-binding protein 2 OS=Homo sapiens GN=CTBP2 PE=1 SV=1
Q9NTJ3	SMC4	Structural maintenance of chromosomes protein 4 OS=Homo sapiens GN=SMC4 PE=1 SV=
Q12824	SNF5	SWI/SNF-related matrix-associated actin-dependent regulator of chromatin subfamily B m SV=2
P78527	PRKDC	DNA-dependent protein kinase catalytic subunit OS=Homo sapiens GN=PRKDC PE=1 SV=3
P24534	EF1B	Elongation factor 1-beta OS=Homo sapiens GN=EEF1B2 PE=1 SV=3
P35244	RFA3	Replication protein A 14 kDa subunit OS=Homo sapiens GN=RPA3 PE=1 SV=1
Q5VTR2	BRE1A	E3 ubiquitin-protein ligase BRE1A OS=Homo sapiens GN=RNF20 PE=1 SV=2
O15294	OGT1	UDP-N-acetylglucosamine--peptide N-acetylglucosaminyltransferase 110 kDa subunit OS=
Q15291	RBBP5	Retinoblastoma-binding protein 5 OS=Homo sapiens GN=RBBP5 PE=1 SV=2
Q9Y606	TRUA	tRNA pseudouridine synthase A mitochondrial OS=Homo sapiens GN=PUS1 PE=1 SV=3
P41091	IF2G	Eukaryotic translation initiation factor 2 subunit 3 OS=Homo sapiens GN=EIF2S3 PE=1 SV=
Q9H1D9	RPC6	DNA-directed RNA polymerase III subunit RPC6 OS=Homo sapiens GN=POLR3F PE=1 SV=1
Q9Y5U2	TSSC4	Protein TSSC4 OS=Homo sapiens GN=TSSC4 PE=1 SV=3
Q52LJ0	FA98B	Protein FAM98B OS=Homo sapiens GN=FAM98B PE=1 SV=1
Q12931	TRAP1	Heat shock protein 75 kDa mitochondrial OS=Homo sapiens GN=TRAP1 PE=1 SV=3

Q8N163	CCAR2	Cell cycle and apoptosis regulator protein 2 OS=Homo sapiens GN=CCAR2 PE=1 SV=2
Q04637	IF4G1	Eukaryotic translation initiation factor 4 gamma 1 OS=Homo sapiens GN=EIF4G1 PE=1 SV=
Q9P2J5	SYLC	Leucine--tRNA ligase cytoplasmic OS=Homo sapiens GN=LARS PE=1 SV=2
P20042	IF2B	Eukaryotic translation initiation factor 2 subunit 2 OS=Homo sapiens GN=EIF2S2 PE=1 SV=
P31946	1433B	14-3-3 protein beta/alpha OS=Homo sapiens GN=YWHAB PE=1 SV=3
O14602	IF1AY	Eukaryotic translation initiation factor 1A Y-chromosomal OS=Homo sapiens GN=EIF1AY
P47813	IF1AX	Eukaryotic translation initiation factor 1A X-chromosomal OS=Homo sapiens GN=EIF1AX
Q9UKS6	PACN3	Protein kinase C and casein kinase substrate in neurons protein 3 OS=Homo sapiens GN=
P68400	CSK21	Casein kinase II subunit alpha OS=Homo sapiens GN=CSNK2A1 PE=1 SV=1
Q13619	CUL4A	Cullin-4A OS=Homo sapiens GN=CUL4A PE=1 SV=3
Q4VXU2	PAP1L	Polyadenylate-binding protein 1-like OS=Homo sapiens GN=PABPC1L PE=2 SV=1
tr	H0YHG0	Uncharacterized protein (Fragment) OS=Homo sapiens PE=1 SV=1
Q9NQT4	EXOS5	Exosome complex component RRP46 OS=Homo sapiens GN=EXOSC5 PE=1 SV=1
P13010	XRCC5	X-ray repair cross-complementing protein 5 OS=Homo sapiens GN=XRCC5 PE=1 SV=3
O43583	DENR	Density-regulated protein OS=Homo sapiens GN=DENR PE=1 SV=2
Q9UQ35	SRRM2	Serine/arginine repetitive matrix protein 2 OS=Homo sapiens GN=SRRM2 PE=1 SV=2
Q08J23	NSUN2	tRNA (cytosine(34)-C(5))-methyltransferase OS=Homo sapiens GN=NSUN2 PE=1 SV=2
Q8NC51	PAIRB	Plasminogen activator inhibitor 1 RNA-binding protein OS=Homo sapiens GN=SERBP1 PE=
P51991	ROA3	Heterogeneous nuclear ribonucleoprotein A3 OS=Homo sapiens GN=HNRNPA3 PE=1 SV=
P61981	1433G	14-3-3 protein gamma OS=Homo sapiens GN=YWHAG PE=1 SV=2
P43243	MATR3	Matrin-3 OS=Homo sapiens GN=MATR3 PE=1 SV=2
P25789	PSA4	Proteasome subunit alpha type-4 OS=Homo sapiens GN=PSMA4 PE=1 SV=1
P49756	RBM25	RNA-binding protein 25 OS=Homo sapiens GN=RBM25 PE=1 SV=3
Q8NEV1	CSK23	Casein kinase II subunit alpha 3 OS=Homo sapiens GN=CSNK2A3 PE=1 SV=2
P63165	SUMO1	Small ubiquitin-related modifier 1 OS=Homo sapiens GN=SUMO1 PE=1 SV=1
Q2VIR3	IF2GL	Putative eukaryotic translation initiation factor 2 subunit 3-like protein OS=Homo sapiens
Q53GS9	SNUT2	U4/U6.U5 tri-snRNP-associated protein 2 OS=Homo sapiens GN=USP39 PE=1 SV=2
P17844	DDX5	Probable ATP-dependent RNA helicase DDX5 OS=Homo sapiens GN=DDX5 PE=1 SV=1
P33240	CSTF2	Cleavage stimulation factor subunit 2 OS=Homo sapiens GN=CSTF2 PE=1 SV=1
Q9Y5K6	CD2AP	CD2-associated protein OS=Homo sapiens GN=CD2AP PE=1 SV=1
Q9UJW0	DCTN4	Dynactin subunit 4 OS=Homo sapiens GN=DCTN4 PE=1 SV=1
Q66PJ3	AR6P4	ADP-ribosylation factor-like protein 6-interacting protein 4 OS=Homo sapiens GN=ARL6IP
Q16629	SRSF7	Serine/arginine-rich splicing factor 7 OS=Homo sapiens GN=SRSF7 PE=1 SV=1
P25490	TYY1	Transcriptional repressor protein YY1 OS=Homo sapiens GN=YY1 PE=1 SV=2
Q01105	SET	Protein SET OS=Homo sapiens GN=SET PE=1 SV=3
P0DME0	SETLP	Protein SETSIP OS=Homo sapiens GN=SETSIP PE=1 SV=1
Q99583	MNT	Max-binding protein MNT OS=Homo sapiens GN=MNT PE=1 SV=1
P53621	COPA	Coatomer subunit alpha OS=Homo sapiens GN=COPA PE=1 SV=2
O75475	PSIP1	PC4 and SFRS1-interacting protein OS=Homo sapiens GN=PSIP1 PE=1 SV=1
Q01780	EXOSX	Exosome component 10 OS=Homo sapiens GN=EXOSC10 PE=1 SV=2
P33176	KINH	Kinesin-1 heavy chain OS=Homo sapiens GN=KIF5B PE=1 SV=1

Q9Y6E2	BZW2	Basic leucine zipper and W2 domain-containing protein 2 OS=Homo sapiens GN=BZW2 PE
Q14204	DYHC1	Cytoplasmic dynein 1 heavy chain 1 OS=Homo sapiens GN=DYNC1H1 PE=1 SV=5
P48668	K2C6C	Keratin type II cytoskeletal 6C OS=Homo sapiens GN=KRT6C PE=1 SV=3
P49720	PSB3	Proteasome subunit beta type-3 OS=Homo sapiens GN=PSMB3 PE=1 SV=2
O00148	DX39A	ATP-dependent RNA helicase DDX39A OS=Homo sapiens GN=DDX39A PE=1 SV=2
Q9Y295	DRG1	Developmentally-regulated GTP-binding protein 1 OS=Homo sapiens GN=DRG1 PE=1 SV=
P09661	RU2A	U2 small nuclear ribonucleoprotein A' OS=Homo sapiens GN=SNRPA1 PE=1 SV=2
O14745	NHRF1	Na(+)/H(+) exchange regulatory cofactor NHE-RF1 OS=Homo sapiens GN=SLC9A3R1 PE=1
P67870	CSK2B	Casein kinase II subunit beta OS=Homo sapiens GN=CSNK2B PE=1 SV=1
O75909	CCNK	Cyclin-K OS=Homo sapiens GN=CCNK PE=1 SV=2
Q99436	PSB7	Proteasome subunit beta type-7 OS=Homo sapiens GN=PSMB7 PE=1 SV=1
P27348	1433T	14-3-3 protein theta OS=Homo sapiens GN=YWHAQ PE=1 SV=1
O43172	PRP4	U4/U6 small nuclear ribonucleoprotein Prp4 OS=Homo sapiens GN=PRPF4 PE=1 SV=2
P47755	CAZA2	F-actin-capping protein subunit alpha-2 OS=Homo sapiens GN=CAPZA2 PE=1 SV=3
Q9BR76	COR1B	Coronin-1B OS=Homo sapiens GN=CORO1B PE=1 SV=1
P41252	SYIC	Isoleucine--tRNA ligase cytoplasmic OS=Homo sapiens GN=IARS PE=1 SV=2
Q92793	CBP	CREB-binding protein OS=Homo sapiens GN=CREBBP PE=1 SV=3
P51531	SMCA2	Probable global transcription activator SNF2L2 OS=Homo sapiens GN=SMARCA2 PE=1 SV=
Q15024	EXOS7	Exosome complex component RRP42 OS=Homo sapiens GN=EXOSC7 PE=1 SV=3
P47897	SYQ	Glutamine--tRNA ligase OS=Homo sapiens GN=QARS PE=1 SV=1
P57721	PCBP3	Poly(rC)-binding protein 3 OS=Homo sapiens GN=PCBP3 PE=2 SV=2
Q9H0L4	CSTFT	Cleavage stimulation factor subunit 2 tau variant OS=Homo sapiens GN=CSTF2T PE=1 SV=
Q9BRP4	PAAF1	Proteasomal ATPase-associated factor 1 OS=Homo sapiens GN=PAAF1 PE=1 SV=2
Q9H814	PHAX	Phosphorylated adapter RNA export protein OS=Homo sapiens GN=PHAX PE=1 SV=1
P35606	COPB2	Coatomer subunit beta' OS=Homo sapiens GN=COPB2 PE=1 SV=2
P46060	RAGP1	Ran GTPase-activating protein 1 OS=Homo sapiens GN=RANGAP1 PE=1 SV=1
Q8IX18	DHX40	Probable ATP-dependent RNA helicase DHX40 OS=Homo sapiens GN=DHX40 PE=1 SV=2
O75526	RMXL2	RNA-binding motif protein X-linked-like-2 OS=Homo sapiens GN=RBMXL2 PE=1 SV=3
Q92804	RBP56	TATA-binding protein-associated factor 2N OS=Homo sapiens GN=TAF15 PE=1 SV=1
Q9UN86	G3BP2	Ras GTPase-activating protein-binding protein 2 OS=Homo sapiens GN=G3BP2 PE=1 SV=2
Q5VTL8	PR38B	Pre-mRNA-splicing factor 38B OS=Homo sapiens GN=PRPF38B PE=1 SV=1
Q9GZS3	WDR61	WD repeat-containing protein 61 OS=Homo sapiens GN=WDR61 PE=1 SV=1
Q07157	ZO1	Tight junction protein ZO-1 OS=Homo sapiens GN=TJP1 PE=1 SV=3
Q03252	LMNB2	Lamin-B2 OS=Homo sapiens GN=LMNB2 PE=1 SV=4
P28072	PSB6	Proteasome subunit beta type-6 OS=Homo sapiens GN=PSMB6 PE=1 SV=4
Q16763	UBE2S	Ubiquitin-conjugating enzyme E2 S OS=Homo sapiens GN=UBE2S PE=1 SV=2
Q6P1N0	C2D1A	Coiled-coil and C2 domain-containing protein 1A OS=Homo sapiens GN=CC2D1A PE=1 SV=
O43719	HTSF1	HIV Tat-specific factor 1 OS=Homo sapiens GN=HTATSF1 PE=1 SV=1
Q8NE71	ABCF1	ATP-binding cassette sub-family F member 1 OS=Homo sapiens GN=ABCF1 PE=1 SV=2
O95678	K2C75	Keratin type II cytoskeletal 75 OS=Homo sapiens GN=KRT75 PE=1 SV=2
Q9HCS7	SYF1	Pre-mRNA-splicing factor SYF1 OS=Homo sapiens GN=XAB2 PE=1 SV=2

Q15366	PCBP2	Poly(rC)-binding protein 2 OS=Homo sapiens GN=PCBP2 PE=1 SV=1
Q99627	CSN8	COP9 signalosome complex subunit 8 OS=Homo sapiens GN=COPS8 PE=1 SV=1
P50750	CDK9	Cyclin-dependent kinase 9 OS=Homo sapiens GN=CDK9 PE=1 SV=3
O60832	DKC1	H/ACA ribonucleoprotein complex subunit 4 OS=Homo sapiens GN=DKC1 PE=1 SV=3
P05204	HMGN2	Non-histone chromosomal protein HMG-17 OS=Homo sapiens GN=HMGN2 PE=1 SV=3
Q8WZ42	TITIN	Titin OS=Homo sapiens GN=TTN PE=1 SV=4
Q14839	CHD4	Chromodomain-helicase-DNA-binding protein 4 OS=Homo sapiens GN=CHD4 PE=1 SV=2
Q9UKN8	TF3C4	General transcription factor 3C polypeptide 4 OS=Homo sapiens GN=GTF3C4 PE=1 SV=2
Q9UPN6	SCAF8	Protein SCAF8 OS=Homo sapiens GN=SCAF8 PE=1 SV=1
Q6P6C2	ALKB5	RNA demethylase ALKBH5 OS=Homo sapiens GN=ALKBH5 PE=1 SV=2
Q9Y2W2	WBP11	WW domain-binding protein 11 OS=Homo sapiens GN=WBP11 PE=1 SV=1
Q92841	DDX17	Probable ATP-dependent RNA helicase DDX17 OS=Homo sapiens GN=DDX17 PE=1 SV=2
Q06323	PSME1	Proteasome activator complex subunit 1 OS=Homo sapiens GN=PSME1 PE=1 SV=1
P13647	K2C5	Keratin type II cytoskeletal 5 OS=Homo sapiens GN=KRT5 PE=1 SV=3
Q9Y3B4	SF3B6	Splicing factor 3B subunit 6 OS=Homo sapiens GN=SF3B6 PE=1 SV=1
O60884	DNJA2	DnaJ homolog subfamily A member 2 OS=Homo sapiens GN=DNAJA2 PE=1 SV=1
Q9BXS6	NUSAP	Nucleolar and spindle-associated protein 1 OS=Homo sapiens GN=NUSAP1 PE=1 SV=1
Q99584	S10AD	Protein S100-A13 OS=Homo sapiens GN=S100A13 PE=1 SV=1
P63208	SKP1	S-phase kinase-associated protein 1 OS=Homo sapiens GN=SKP1 PE=1 SV=2
O94763	RMP	Unconventional prefoldin RPB5 interactor 1 OS=Homo sapiens GN=URI1 PE=1 SV=3
Q8N7X1	RMXL3	RNA-binding motif protein X-linked-like-3 OS=Homo sapiens GN=RBMXL3 PE=2 SV=2
Q96E39	RMXL1	RNA binding motif protein X-linked-like-1 OS=Homo sapiens GN=RBMXL1 PE=1 SV=1
O14979	HNRDL	Heterogeneous nuclear ribonucleoprotein D-like OS=Homo sapiens GN=HNRNPDL PE=1 S[illegible]
Q08043	ACTN3	Alpha-actinin-3 OS=Homo sapiens GN=ACTN3 PE=1 SV=2
P02786	TFR1	Transferrin receptor protein 1 OS=Homo sapiens GN=TFRC PE=1 SV=2
Q9BRL6	SRSF8	Serine/arginine-rich splicing factor 8 OS=Homo sapiens GN=SRSF8 PE=1 SV=1
Q15287	RNPS1	RNA-binding protein with serine-rich domain 1 OS=Homo sapiens GN=RNPS1 PE=1 SV=1
O60282	KIF5C	Kinesin heavy chain isoform 5C OS=Homo sapiens GN=KIF5C PE=1 SV=1
Q12840	KIF5A	Kinesin heavy chain isoform 5A OS=Homo sapiens GN=KIF5A PE=1 SV=2
Q13620	CUL4B	Cullin-4B OS=Homo sapiens GN=CUL4B PE=1 SV=4
O95232	LC7L3	Luc7-like protein 3 OS=Homo sapiens GN=LUC7L3 PE=1 SV=2
Q99622	C10	Protein C10 OS=Homo sapiens GN=C12orf57 PE=1 SV=1
P12107	COBA1	Collagen alpha-1(XI) chain OS=Homo sapiens GN=COL11A1 PE=1 SV=4
Q96B26	EXOS8	Exosome complex component RRP43 OS=Homo sapiens GN=EXOSC8 PE=1 SV=1
P42771	CDN2A	Cyclin-dependent kinase inhibitor 2A OS=Homo sapiens GN=CDKN2A PE=1 SV=2
P23284	PPIB	Peptidyl-prolyl cis-trans isomerase B OS=Homo sapiens GN=PPIB PE=1 SV=2
P50990	TCPQ	T-complex protein 1 subunit theta OS=Homo sapiens GN=CCT8 PE=1 SV=4
Q92769	HDAC2	Histone deacetylase 2 OS=Homo sapiens GN=HDAC2 PE=1 SV=2
O43396	TXNL1	Thioredoxin-like protein 1 OS=Homo sapiens GN=TXNL1 PE=1 SV=3
Q8IZQ5	SELH	Selenoprotein H OS=Homo sapiens GN=SELH PE=1 SV=2
P25786	PSA1	Proteasome subunit alpha type-1 OS=Homo sapiens GN=PSMA1 PE=1 SV=1

Q5JWF2	GNAS1	Guanine nucleotide-binding protein G(s) subunit alpha isoforms XLas OS=Homo sapiens G
P62993	GRB2	Growth factor receptor-bound protein 2 OS=Homo sapiens GN=GRB2 PE=1 SV=1
P63167	DYL1	Dynein light chain 1 cytoplasmic OS=Homo sapiens GN=DYNLL1 PE=1 SV=1
Q9NPA8	ENY2	Transcription and mRNA export factor ENY2 OS=Homo sapiens GN=ENY2 PE=1 SV=1
Q16186	ADRM1	Proteasomal ubiquitin receptor ADRM1 OS=Homo sapiens GN=ADRM1 PE=1 SV=2
Q8TDI0	CHD5	Chromodomain-helicase-DNA-binding protein 5 OS=Homo sapiens GN=CHD5 PE=1 SV=1
Q15555	MARE2	Microtubule-associated protein RP/EB family member 2 OS=Homo sapiens GN=MAPRE2 P
Q8WW12	PCNP	PEST proteolytic signal-containing nuclear protein OS=Homo sapiens GN=PCNP PE=1 SV=2
P62857	RS28	40S ribosomal protein S28 OS=Homo sapiens GN=RPS28 PE=1 SV=1
P04792	HSPB1	Heat shock protein beta-1 OS=Homo sapiens GN=HSPB1 PE=1 SV=2
Q96EN8	MOCOS	Molybdenum cofactor sulfurase OS=Homo sapiens GN=MOCOS PE=1 SV=2
O00712	NFIB	Nuclear factor 1 B-type OS=Homo sapiens GN=NFIB PE=1 SV=2
O75934	SPF27	Pre-mRNA-splicing factor SPF27 OS=Homo sapiens GN=BCAS2 PE=1 SV=1
P30419	NMT1	Glycylpeptide N-tetradecanoyltransferase 1 OS=Homo sapiens GN=NMT1 PE=1 SV=2
Q9NRL2	BAZ1A	Bromodomain adjacent to zinc finger domain protein 1A OS=Homo sapiens GN=BAZ1A PE
P48444	COPD	Coatomer subunit delta OS=Homo sapiens GN=ARCN1 PE=1 SV=1
P49458	SRP09	Signal recognition particle 9 kDa protein OS=Homo sapiens GN=SRP9 PE=1 SV=2
P11277	SPTB1	Spectrin beta chain erythrocytic OS=Homo sapiens GN=SPTB PE=1 SV=5
Q9BVC3	DCC1	Sister chromatid cohesion protein DCC1 OS=Homo sapiens GN=DSCC1 PE=1 SV=2
O43175	SERA	D-3-phosphoglycerate dehydrogenase OS=Homo sapiens GN=PHGDH PE=1 SV=4
P60842	IF4A1	Eukaryotic initiation factor 4A-I OS=Homo sapiens GN=EIF4A1 PE=1 SV=1
Q14240	IF4A2	Eukaryotic initiation factor 4A-II OS=Homo sapiens GN=EIF4A2 PE=1 SV=2
Q9BXP5	SRRT	Serrate RNA effector molecule homolog OS=Homo sapiens GN=SRRT PE=1 SV=1
Q8N1G2	CMTR1	Cap-specific mRNA (nucleoside-2'-O-)-methyltransferase 1 OS=Homo sapiens GN=CMTR1
Q06587	RING1	E3 ubiquitin-protein ligase RING1 OS=Homo sapiens GN=RING1 PE=1 SV=2
O43633	CHM2A	Charged multivesicular body protein 2a OS=Homo sapiens GN=CHMP2A PE=1 SV=1
P02461	CO3A1	Collagen alpha-1(III) chain OS=Homo sapiens GN=COL3A1 PE=1 SV=4
P63162	RSMN	Small nuclear ribonucleoprotein-associated protein N OS=Homo sapiens GN=SNRPN PE=1
Q9BQ67	GRWD1	Glutamate-rich WD repeat-containing protein 1 OS=Homo sapiens GN=GRWD1 PE=1 SV=
P49721	PSB2	Proteasome subunit beta type-2 OS=Homo sapiens GN=PSMB2 PE=1 SV=1
Q96I24	FUBP3	Far upstream element-binding protein 3 OS=Homo sapiens GN=FUBP3 PE=1 SV=2
Q9GZP4	PITH1	PITH domain-containing protein 1 OS=Homo sapiens GN=PITHD1 PE=1 SV=1
Q86W42	THOC6	THO complex subunit 6 homolog OS=Homo sapiens GN=THOC6 PE=1 SV=1
Q15843	NEDD8	NEDD8 OS=Homo sapiens GN=NEDD8 PE=1 SV=1
tr	E9PL57	Protein NEDD8-MDP1 (Fragment) OS=Homo sapiens GN=NEDD8-MDP1 PE=4 SV=1
P14868	SYDC	Aspartate--tRNA ligase cytoplasmic OS=Homo sapiens GN=DARS PE=1 SV=2
Q96SI9	STRBP	Spermatid perinuclear RNA-binding protein OS=Homo sapiens GN=STRBP PE=1 SV=1
Q13151	ROA0	Heterogeneous nuclear ribonucleoprotein A0 OS=Homo sapiens GN=HNRNPA0 PE=1 SV=
Q9Y5Z7	HCFC2	Host cell factor 2 OS=Homo sapiens GN=HCFC2 PE=1 SV=1
P43034	LIS1	Platelet-activating factor acetylhydrolase IB subunit alpha OS=Homo sapiens GN=PAFAH1
Q14974	IMB1	Importin subunit beta-1 OS=Homo sapiens GN=KPNB1 PE=1 SV=2

P78318	IGBP1	Immunoglobulin-binding protein 1 OS=Homo sapiens GN=IGBP1 PE=1 SV=1
P20290	BTF3	Transcription factor BTF3 OS=Homo sapiens GN=BTF3 PE=1 SV=1
Q9BQ61	CS043	Uncharacterized protein C19orf43 OS=Homo sapiens GN=C19orf43 PE=1 SV=1
Q14258	TRI25	E3 ubiquitin/ISG15 ligase TRIM25 OS=Homo sapiens GN=TRIM25 PE=1 SV=2
Q9BWU0	NADAP	Kanadaptin OS=Homo sapiens GN=SLC4A1AP PE=1 SV=1
Q15436	SC23A	Protein transport protein Sec23A OS=Homo sapiens GN=SEC23A PE=1 SV=2
Q9NUW8	TYDP1	Tyrosyl-DNA phosphodiesterase 1 OS=Homo sapiens GN=TDP1 PE=1 SV=2
P59998	ARPC4	Actin-related protein 2/3 complex subunit 4 OS=Homo sapiens GN=ARPC4 PE=1 SV=3
tr	A0A0A6YYG9	Protein ARPC4-TTLL3 OS=Homo sapiens GN=ARPC4-TTLL3 PE=4 SV=1
Q14151	SAFB2	Scaffold attachment factor B2 OS=Homo sapiens GN=SAFB2 PE=1 SV=1
P52655	TF2AA	Transcription initiation factor IIA subunit 1 OS=Homo sapiens GN=GTF2A1 PE=1 SV=1
Q460N5	PAR14	Poly [ADP-ribose] polymerase 14 OS=Homo sapiens GN=PARP14 PE=1 SV=3
P38646	GRP75	Stress-70 protein mitochondrial OS=Homo sapiens GN=HSPA9 PE=1 SV=2
Q9Y4E8	UBP15	Ubiquitin carboxyl-terminal hydrolase 15 OS=Homo sapiens GN=USP15 PE=1 SV=3
Q96FX7	TRM61	tRNA (adenine(58)-N(1))-methyltransferase catalytic subunit TRMT61A OS=Homo sapiens
O75940	SPF30	Survival of motor neuron-related-splicing factor 30 OS=Homo sapiens GN=SMNDC1 PE=1
Q9BT78	CSN4	COP9 signalosome complex subunit 4 OS=Homo sapiens GN=COPS4 PE=1 SV=1
P07910	HNRPC	Heterogeneous nuclear ribonucleoproteins C1/C2 OS=Homo sapiens GN=HNRNPC PE=1 S
P61289	PSME3	Proteasome activator complex subunit 3 OS=Homo sapiens GN=PSME3 PE=1 SV=1
Q8NCF5	NF2IP	NFATC2-interacting protein OS=Homo sapiens GN=NFATC2IP PE=1 SV=1
Q9Y333	LSM2	U6 snRNA-associated Sm-like protein LSm2 OS=Homo sapiens GN=LSM2 PE=1 SV=1
Q5XKE5	K2C79	Keratin type II cytoskeletal 79 OS=Homo sapiens GN=KRT79 PE=1 SV=2
P61244	MAX	Protein max OS=Homo sapiens GN=MAX PE=1 SV=1
Q9UI30	TR112	Multifunctional methyltransferase subunit TRM112-like protein OS=Homo sapiens GN=TR
Q9UPT8	ZC3H4	Zinc finger CCCH domain-containing protein 4 OS=Homo sapiens GN=ZC3H4 PE=1 SV=3
Q96SB4	SRPK1	SRSF protein kinase 1 OS=Homo sapiens GN=SRPK1 PE=1 SV=2
Q9ULC4	MCTS1	Malignant T-cell-amplified sequence 1 OS=Homo sapiens GN=MCTS1 PE=1 SV=1
Q86VP6	CAND1	Cullin-associated NEDD8-dissociated protein 1 OS=Homo sapiens GN=CAND1 PE=1 SV=2
Q96C19	EFHD2	EF-hand domain-containing protein D2 OS=Homo sapiens GN=EFHD2 PE=1 SV=1
Q8WUF8	F172A	Protein FAM172A OS=Homo sapiens GN=FAM172A PE=2 SV=1
O75717	WDHD1	WD repeat and HMG-box DNA-binding protein 1 OS=Homo sapiens GN=WDHD1 PE=1 SV=
Q9H2H8	PPIL3	Peptidyl-prolyl cis-trans isomerase-like 3 OS=Homo sapiens GN=PPIL3 PE=1 SV=1
P19338	NUCL	Nucleolin OS=Homo sapiens GN=NCL PE=1 SV=3
Q9Y5B9	SP16H	FACT complex subunit SPT16 OS=Homo sapiens GN=SUPT16H PE=1 SV=1
Q8TER5	ARH40	Rho guanine nucleotide exchange factor 40 OS=Homo sapiens GN=ARHGEF40 PE=1 SV=3
P22105	TENX	Tenascin-X OS=Homo sapiens GN=TNXB PE=1 SV=4
Q9Y294	ASF1A	Histone chaperone ASF1A OS=Homo sapiens GN=ASF1A PE=1 SV=1
P43487	RANG	Ran-specific GTPase-activating protein OS=Homo sapiens GN=RANBP1 PE=1 SV=1
Q13616	CUL1	Cullin-1 OS=Homo sapiens GN=CUL1 PE=1 SV=2
Q9C0C2	TB182	182 kDa tankyrase-1-binding protein OS=Homo sapiens GN=TNKS1BP1 PE=1 SV=4
Q92616	GCN1	eIF-2-alpha kinase activator GCN1 OS=Homo sapiens GN=GCN1 PE=1 SV=6

Q86W56	PARG	Poly(ADP-ribose) glycohydrolase OS=Homo sapiens GN=PARG PE=1 SV=1
Q92538	GBF1	Golgi-specific brefeldin A-resistance guanine nucleotide exchange factor 1 OS=Homo sapi
P52272	HNRPM	Heterogeneous nuclear ribonucleoprotein M OS=Homo sapiens GN=HNRNPM PE=1 SV=3
O00571	DDX3X	ATP-dependent RNA helicase DDX3X OS=Homo sapiens GN=DDX3X PE=1 SV=3
O15523	DDX3Y	ATP-dependent RNA helicase DDX3Y OS=Homo sapiens GN=DDX3Y PE=1 SV=2
Q12904	AIMP1	Aminoacyl tRNA synthase complex-interacting multifunctional protein 1 OS=Homo sapien
Q9NP97	DLRB1	Dynein light chain roadblock-type 1 OS=Homo sapiens GN=DYNLRB1 PE=1 SV=3
O60841	IF2P	Eukaryotic translation initiation factor 5B OS=Homo sapiens GN=EIF5B PE=1 SV=4
O00268	TAF4	Transcription initiation factor TFIID subunit 4 OS=Homo sapiens GN=TAF4 PE=1 SV=2
P08754	GNAI3	Guanine nucleotide-binding protein G(k) subunit alpha OS=Homo sapiens GN=GNAI3 PE=
P38405	GNAL	Guanine nucleotide-binding protein G(olf) subunit alpha OS=Homo sapiens GN=GNAL PE=
P04899	GNAI2	Guanine nucleotide-binding protein G(i) subunit alpha-2 OS=Homo sapiens GN=GNAI2 PE
A8MTJ3	GNAT3	Guanine nucleotide-binding protein G(t) subunit alpha-3 OS=Homo sapiens GN=GNAT3 P
P55884	EIF3B	Eukaryotic translation initiation factor 3 subunit B OS=Homo sapiens GN=EIF3B PE=1 SV=
P40425	PBX2	Pre-B-cell leukemia transcription factor 2 OS=Homo sapiens GN=PBX2 PE=1 SV=2
P48556	PSMD8	26S proteasome non-ATPase regulatory subunit 8 OS=Homo sapiens GN=PSMD8 PE=1 SV
O75822	EIF3J	Eukaryotic translation initiation factor 3 subunit J OS=Homo sapiens GN=EIF3J PE=1 SV=2
Q8TBX8	PI42C	Phosphatidylinositol 5-phosphate 4-kinase type-2 gamma OS=Homo sapiens GN=PIP4K2C
P51784	UBP11	Ubiquitin carboxyl-terminal hydrolase 11 OS=Homo sapiens GN=USP11 PE=1 SV=3
Q9H0G5	NSRP1	Nuclear speckle splicing regulatory protein 1 OS=Homo sapiens GN=NSRP1 PE=1 SV=1
Q9NRY2	SOSSC	SOSS complex subunit C OS=Homo sapiens GN=INIP PE=1 SV=1
Q9UBC2	EP15R	Epidermal growth factor receptor substrate 15-like 1 OS=Homo sapiens GN=EPS15L1 PE=
P62937	PPIA	Peptidyl-prolyl cis-trans isomerase A OS=Homo sapiens GN=PPIA PE=1 SV=2
Q9NW82	WDR70	WD repeat-containing protein 70 OS=Homo sapiens GN=WDR70 PE=1 SV=1
O60524	NEMF	Nuclear export mediator factor NEMF OS=Homo sapiens GN=NEMF PE=1 SV=4
Q14181	DPOA2	DNA polymerase alpha subunit B OS=Homo sapiens GN=POLA2 PE=1 SV=2
P54727	RD23B	UV excision repair protein RAD23 homolog B OS=Homo sapiens GN=RAD23B PE=1 SV=1
P13942	COBA2	Collagen alpha-2(XI) chain OS=Homo sapiens GN=COL11A2 PE=1 SV=5
P49643	PRI2	DNA primase large subunit OS=Homo sapiens GN=PRIM2 PE=1 SV=2
Q9NX24	NHP2	H/ACA ribonucleoprotein complex subunit 2 OS=Homo sapiens GN=NHP2 PE=1 SV=1
Q5JUK3	KCNT1	Potassium channel subfamily T member 1 OS=Homo sapiens GN=KCNT1 PE=1 SV=2
P62195	PRS8	26S protease regulatory subunit 8 OS=Homo sapiens GN=PSMC5 PE=1 SV=1
Q9H6T3	RPAP3	RNA polymerase II-associated protein 3 OS=Homo sapiens GN=RPAP3 PE=1 SV=2
Q9NV56	MRGBP	MRG/MORF4L-binding protein OS=Homo sapiens GN=MRGBP PE=1 SV=1
Q9Y224	CN166	UPF0568 protein C14orf166 OS=Homo sapiens GN=C14orf166 PE=1 SV=1
O15357	SHIP2	Phosphatidylinositol 3 4 5-trisphosphate 5-phosphatase 2 OS=Homo sapiens GN=INPPL1
Q96JJ3	ELMO2	Engulfment and cell motility protein 2 OS=Homo sapiens GN=ELMO2 PE=1 SV=2
Q9NVP2	ASF1B	Histone chaperone ASF1B OS=Homo sapiens GN=ASF1B PE=1 SV=1
Q9GZN8	CT027	UPF0687 protein C20orf27 OS=Homo sapiens GN=C20orf27 PE=1 SV=3
Q9UHV9	PFD2	Prefoldin subunit 2 OS=Homo sapiens GN=PFDN2 PE=1 SV=1
P18615	NELFE	Negative elongation factor E OS=Homo sapiens GN=NELFE PE=1 SV=3

O76003	GLRX3	Glutaredoxin-3 OS=Homo sapiens GN=GLRX3 PE=1 SV=2
Q15637	SF01	Splicing factor 1 OS=Homo sapiens GN=SF1 PE=1 SV=4
Q96ST3	SIN3A	Paired amphipathic helix protein Sin3a OS=Homo sapiens GN=SIN3A PE=1 SV=2
P48634	PRC2A	Protein PRRC2A OS=Homo sapiens GN=PRRC2A PE=1 SV=3
Q9BUQ8	DDX23	Probable ATP-dependent RNA helicase DDX23 OS=Homo sapiens GN=DDX23 PE=1 SV=3
Q8IYJ1	CPNE9	Copine-9 OS=Homo sapiens GN=CPNE9 PE=1 SV=3
Q86YQ8	CPNE8	Copine-8 OS=Homo sapiens GN=CPNE8 PE=1 SV=2
Q9HCH3	CPNE5	Copine-5 OS=Homo sapiens GN=CPNE5 PE=1 SV=2
O75155	CAND2	Cullin-associated NEDD8-dissociated protein 2 OS=Homo sapiens GN=CAND2 PE=1 SV=3
P61601	NCALD	Neurocalcin-delta OS=Homo sapiens GN=NCALD PE=1 SV=2
Q9Y566	SHAN1	SH3 and multiple ankyrin repeat domains protein 1 OS=Homo sapiens GN=SHANK1 PE=1 [illegible]
P0DN76	U2AF5	Splicing factor U2AF 35 kDa subunit-like protein OS=Homo sapiens GN=U2AF1L5 PE=1 SV[illegible]
Q9H269	VPS16	Vacuolar protein sorting-associated protein 16 homolog OS=Homo sapiens GN=VPS16 PE[illegible]
O96013	PAK4	Serine/threonine-protein kinase PAK 4 OS=Homo sapiens GN=PAK4 PE=1 SV=1
Q92620	PRP16	Pre-mRNA-splicing factor ATP-dependent RNA helicase PRP16 OS=Homo sapiens GN=DH[illegible]
P62304	RUXE	Small nuclear ribonucleoprotein E OS=Homo sapiens GN=SNRPE PE=1 SV=1
Q9H078	CLPB	Caseinolytic peptidase B protein homolog OS=Homo sapiens GN=CLPB PE=1 SV=1
Q96PU8	QKI	Protein quaking OS=Homo sapiens GN=QKI PE=1 SV=1
P35241	RADI	Radixin OS=Homo sapiens GN=RDX PE=1 SV=1
P85037	FOXK1	Forkhead box protein K1 OS=Homo sapiens GN=FOXK1 PE=1 SV=1
P48729	KC1A	Casein kinase I isoform alpha OS=Homo sapiens GN=CSNK1A1 PE=1 SV=2
Q9BY42	RTF2	Protein RTF2 homolog OS=Homo sapiens GN=RTFDC1 PE=1 SV=3
Q9NQT5	EXOS3	Exosome complex component RRP40 OS=Homo sapiens GN=EXOSC3 PE=1 SV=3
Q14D33	RTP5	Receptor-transporting protein 5 OS=Homo sapiens GN=RTP5 PE=1 SV=2
Q2VWA4	SKOR2	SKI family transcriptional corepressor 2 OS=Homo sapiens GN=SKOR2 PE=1 SV=2
Q9Y490	TLN1	Talin-1 OS=Homo sapiens GN=TLN1 PE=1 SV=3
Q9P289	STK26	Serine/threonine-protein kinase 26 OS=Homo sapiens GN=STK26 PE=1 SV=2
Q9Y6E0	STK24	Serine/threonine-protein kinase 24 OS=Homo sapiens GN=STK24 PE=1 SV=1
P06576	ATPB	ATP synthase subunit beta mitochondrial OS=Homo sapiens GN=ATP5B PE=1 SV=3
Q9Y5L4	TIM13	Mitochondrial import inner membrane translocase subunit Tim13 OS=Homo sapiens GN[illegible]
P57723	PCBP4	Poly(rC)-binding protein 4 OS=Homo sapiens GN=PCBP4 PE=2 SV=1
Q8TD16	BICD2	Protein bicaudal D homolog 2 OS=Homo sapiens GN=BICD2 PE=1 SV=1
O95071	UBR5	E3 ubiquitin-protein ligase UBR5 OS=Homo sapiens GN=UBR5 PE=1 SV=2
Q96F63	CCD97	Coiled-coil domain-containing protein 97 OS=Homo sapiens GN=CCDC97 PE=1 SV=1
P58107	EPIPL	Epiplakin OS=Homo sapiens GN=EPPK1 PE=1 SV=2
Q9Y4C8	RBM19	Probable RNA-binding protein 19 OS=Homo sapiens GN=RBM19 PE=1 SV=3
Q13428	TCOF	Treacle protein OS=Homo sapiens GN=TCOF1 PE=1 SV=3
Q9BUL8	PDC10	Programmed cell death protein 10 OS=Homo sapiens GN=PDCD10 PE=1 SV=1
Q9NTI5	PDS5B	Sister chromatid cohesion protein PDS5 homolog B OS=Homo sapiens GN=PDS5B PE=1 S[illegible]
Q96HC4	PDLI5	PDZ and LIM domain protein 5 OS=Homo sapiens GN=PDLIM5 PE=1 SV=5
Q96NB3	ZN830	Zinc finger protein 830 OS=Homo sapiens GN=ZNF830 PE=1 SV=2

Q06265	EXOS9	Exosome complex component RRP45 OS=Homo sapiens GN=EXOSC9 PE=1 SV=3
Q15417	CNN3	Calponin-3 OS=Homo sapiens GN=CNN3 PE=1 SV=1
Q86UQ4	ABCAD	ATP-binding cassette sub-family A member 13 OS=Homo sapiens GN=ABCA13 PE=2 SV=3
Q9UKX7	NUP50	Nuclear pore complex protein Nup50 OS=Homo sapiens GN=NUP50 PE=1 SV=2
O00287	RFXAP	Regulatory factor X-associated protein OS=Homo sapiens GN=RFXAP PE=1 SV=1
Q8WU90	ZC3HF	Zinc finger CCCH domain-containing protein 15 OS=Homo sapiens GN=ZC3H15 PE=1 SV=1
Q9UPQ0	LIMC1	LIM and calponin homology domains-containing protein 1 OS=Homo sapiens GN=LIMCH1
Q12996	CSTF3	Cleavage stimulation factor subunit 3 OS=Homo sapiens GN=CSTF3 PE=1 SV=1
P35659	DEK	Protein DEK OS=Homo sapiens GN=DEK PE=1 SV=1
Q9P218	COKA1	Collagen alpha-1(XX) chain OS=Homo sapiens GN=COL20A1 PE=1 SV=4
Q99873	ANM1	Protein arginine N-methyltransferase 1 OS=Homo sapiens GN=PRMT1 PE=1 SV=2
Q15025	TNIP1	TNFAIP3-interacting protein 1 OS=Homo sapiens GN=TNIP1 PE=1 SV=2
Q14331	FRG1	Protein FRG1 OS=Homo sapiens GN=FRG1 PE=1 SV=1
Q68CQ4	DIEXF	Digestive organ expansion factor homolog OS=Homo sapiens GN=DIEXF PE=1 SV=2
Q8NEM2	SHCBP	SHC SH2 domain-binding protein 1 OS=Homo sapiens GN=SHCBP1 PE=1 SV=3
Q14814	MEF2D	Myocyte-specific enhancer factor 2D OS=Homo sapiens GN=MEF2D PE=1 SV=1
Q14008	CKAP5	Cytoskeleton-associated protein 5 OS=Homo sapiens GN=CKAP5 PE=1 SV=3
O95400	CD2B2	CD2 antigen cytoplasmic tail-binding protein 2 OS=Homo sapiens GN=CD2BP2 PE=1 SV=1
Q9UKV3	ACINU	Apoptotic chromatin condensation inducer in the nucleus OS=Homo sapiens GN=ACIN1 P
P11279	LAMP1	Lysosome-associated membrane glycoprotein 1 OS=Homo sapiens GN=LAMP1 PE=1 SV=3
Q86UV5	UBP48	Ubiquitin carboxyl-terminal hydrolase 48 OS=Homo sapiens GN=USP48 PE=1 SV=1
tr	C9J1V9	HCG2043275 OS=Homo sapiens GN=EEF1E1-BLOC1S5 PE=4 SV=2
O43324	MCA3	Eukaryotic translation elongation factor 1 epsilon-1 OS=Homo sapiens GN=EEF1E1 PE=1 S
Q9UKA9	PTBP2	Polypyrimidine tract-binding protein 2 OS=Homo sapiens GN=PTBP2 PE=1 SV=1
P17535	JUND	Transcription factor jun-D OS=Homo sapiens GN=JUND PE=1 SV=3
Q9H2U1	DHX36	ATP-dependent RNA helicase DHX36 OS=Homo sapiens GN=DHX36 PE=1 SV=2
Q8IZ73	RUSD2	RNA pseudouridylate synthase domain-containing protein 2 OS=Homo sapiens GN=RPUSI
O75821	EIF3G	Eukaryotic translation initiation factor 3 subunit G OS=Homo sapiens GN=EIF3G PE=1 SV=
P35670	ATP7B	Copper-transporting ATPase 2 OS=Homo sapiens GN=ATP7B PE=1 SV=4
P17480	UBF1	Nucleolar transcription factor 1 OS=Homo sapiens GN=UBTF PE=1 SV=1
P02452	CO1A1	Collagen alpha-1(I) chain OS=Homo sapiens GN=COL1A1 PE=1 SV=5
Q93052	LPP	Lipoma-preferred partner OS=Homo sapiens GN=LPP PE=1 SV=1
Q13796	SHRM2	Protein Shroom2 OS=Homo sapiens GN=SHROOM2 PE=1 SV=1
Q9H1B7	I2BPL	Interferon regulatory factor 2-binding protein-like OS=Homo sapiens GN=IRF2BPL PE=1 S\
P05771	KPCB	Protein kinase C beta type OS=Homo sapiens GN=PRKCB PE=1 SV=4
Q5ST30	SYVM	Valine--tRNA ligase mitochondrial OS=Homo sapiens GN=VARS2 PE=1 SV=2
Q12802	AKP13	A-kinase anchor protein 13 OS=Homo sapiens GN=AKAP13 PE=1 SV=2
Q8IXJ9	ASXL1	Putative Polycomb group protein ASXL1 OS=Homo sapiens GN=ASXL1 PE=1 SV=3
Q9UJA5	TRM6	tRNA (adenine(58)-N(1))-methyltransferase non-catalytic subunit TRM6 OS=Homo sapien
Q15007	FL2D	Pre-mRNA-splicing regulator WTAP OS=Homo sapiens GN=WTAP PE=1 SV=2
O95229	ZWINT	ZW10 interactor OS=Homo sapiens GN=ZWINT PE=1 SV=2

P50991	TCPD	T-complex protein 1 subunit delta OS=Homo sapiens GN=CCT4 PE=1 SV=4
Q5RKV6	EXOS6	Exosome complex component MTR3 OS=Homo sapiens GN=EXOSC6 PE=1 SV=1
Q96SZ4	ZSC10	Zinc finger and SCAN domain-containing protein 10 OS=Homo sapiens GN=ZSCAN10 PE=1
O43765	SGTA	Small glutamine-rich tetratricopeptide repeat-containing protein alpha OS=Homo sapiens
Q6ZUT6	CO052	Uncharacterized protein C15orf52 OS=Homo sapiens GN=C15orf52 PE=1 SV=1
Q9Y448	SKAP	Small kinetochore-associated protein OS=Homo sapiens GN=KNSTRN PE=1 SV=2
Q9BTC0	DIDO1	Death-inducer obliterator 1 OS=Homo sapiens GN=DIDO1 PE=1 SV=5
Q9NQG5	RPR1B	Regulation of nuclear pre-mRNA domain-containing protein 1B OS=Homo sapiens GN=RP
Q9NZT2	OGFR	Opioid growth factor receptor OS=Homo sapiens GN=OGFR PE=1 SV=3
Q6ZSJ8	CA122	Uncharacterized protein C1orf122 OS=Homo sapiens GN=C1orf122 PE=4 SV=2
Q8NFW1	COMA1	Collagen alpha-1(XXII) chain OS=Homo sapiens GN=COL22A1 PE=2 SV=2
Q01826	SATB1	DNA-binding protein SATB1 OS=Homo sapiens GN=SATB1 PE=1 SV=1
P13639	EF2	Elongation factor 2 OS=Homo sapiens GN=EEF2 PE=1 SV=4
Q5CZC0	FSIP2	Fibrous sheath-interacting protein 2 OS=Homo sapiens GN=FSIP2 PE=2 SV=4
P01891	1A68	HLA class I histocompatibility antigen A-68 alpha chain OS=Homo sapiens GN=HLA-A PE=
Q9ULL5	PRR12	Proline-rich protein 12 OS=Homo sapiens GN=PRR12 PE=1 SV=2
Q8N2R8	FA43A	Protein FAM43A OS=Homo sapiens GN=FAM43A PE=2 SV=2
Q8TDM6	DLG5	Disks large homolog 5 OS=Homo sapiens GN=DLG5 PE=1 SV=4
P39748	FEN1	Flap endonuclease 1 OS=Homo sapiens GN=FEN1 PE=1 SV=1
P42025	ACTY	Beta-centractin OS=Homo sapiens GN=ACTR1B PE=1 SV=1
Q9NX74	DUS2L	tRNA-dihydrouridine(20) synthase [NAD(P)+]-like OS=Homo sapiens GN=DUS2 PE=1 SV=1
Q92888	ARHG1	Rho guanine nucleotide exchange factor 1 OS=Homo sapiens GN=ARHGEF1 PE=1 SV=2
P02458	CO2A1	Collagen alpha-1(II) chain OS=Homo sapiens GN=COL2A1 PE=1 SV=3
P19793	RXRA	Retinoic acid receptor RXR-alpha OS=Homo sapiens GN=RXRA PE=1 SV=1
P48443	RXRG	Retinoic acid receptor RXR-gamma OS=Homo sapiens GN=RXRG PE=1 SV=1
Q8WXF1	PSPC1	Paraspeckle component 1 OS=Homo sapiens GN=PSPC1 PE=1 SV=1
Q8WUD4	CCD12	Coiled-coil domain-containing protein 12 OS=Homo sapiens GN=CCDC12 PE=1 SV=1
Q32P44	EMAL3	Echinoderm microtubule-associated protein-like 3 OS=Homo sapiens GN=EML3 PE=1 SV=
Q8TF61	FBX41	F-box only protein 41 OS=Homo sapiens GN=FBXO41 PE=2 SV=5
Q8NC06	ACBD4	Acyl-CoA-binding domain-containing protein 4 OS=Homo sapiens GN=ACBD4 PE=1 SV=2
Q70Z53	F10C1	Protein FRA10AC1 OS=Homo sapiens GN=FRA10AC1 PE=1 SV=3
O43815	STRN	Striatin OS=Homo sapiens GN=STRN PE=1 SV=4
tr	D6RIA3	Protein LOC285556 OS=Homo sapiens GN=LOC285556 PE=4 SV=1
P39880	CUX1	Homeobox protein cut-like 1 OS=Homo sapiens GN=CUX1 PE=1 SV=3
A7E2V4	ZSWM8	Zinc finger SWIM domain-containing protein 8 OS=Homo sapiens GN=ZSWIM8 PE=1 SV=1
Q6QEF8	CORO6	Coronin-6 OS=Homo sapiens GN=CORO6 PE=1 SV=2
P18846	ATF1	Cyclic AMP-dependent transcription factor ATF-1 OS=Homo sapiens GN=ATF1 PE=1 SV=2
Q14320	FA50A	Protein FAM50A OS=Homo sapiens GN=FAM50A PE=1 SV=2
Q13111	CAF1A	Chromatin assembly factor 1 subunit A OS=Homo sapiens GN=CHAF1A PE=1 SV=2
Q29RF7	PDS5A	Sister chromatid cohesion protein PDS5 homolog A OS=Homo sapiens GN=PDS5A PE=1 SV
Q92925	SMRD2	SWI/SNF-related matrix-associated actin-dependent regulator of chromatin subfamily D r SV=3

P05787	K2C8	Keratin type II cytoskeletal 8 OS=Homo sapiens GN=KRT8 PE=1 SV=7
O75340	PDCD6	Programmed cell death protein 6 OS=Homo sapiens GN=PDCD6 PE=1 SV=1
Q86UR5	RIMS1	Regulating synaptic membrane exocytosis protein 1 OS=Homo sapiens GN=RIMS1 PE=1 S
O14654	IRS4	Insulin receptor substrate 4 OS=Homo sapiens GN=IRS4 PE=1 SV=1
O75376	NCOR1	Nuclear receptor corepressor 1 OS=Homo sapiens GN=NCOR1 PE=1 SV=2
P45974	UBP5	Ubiquitin carboxyl-terminal hydrolase 5 OS=Homo sapiens GN=USP5 PE=1 SV=2
Q96HR8	NAF1	H/ACA ribonucleoprotein complex non-core subunit NAF1 OS=Homo sapiens GN=NAF1 P
Q5VW00	DC122	DDB1- and CUL4-associated factor 12-like protein 2 OS=Homo sapiens GN=DCAF12L2 PE=
Q5T6F0	DCA12	DDB1- and CUL4-associated factor 12 OS=Homo sapiens GN=DCAF12 PE=1 SV=1
Q9C0G6	DYH6	Dynein heavy chain 6 axonemal OS=Homo sapiens GN=DNAH6 PE=2 SV=3
Q9UBP6	TRMB	tRNA (guanine-N(7)-)-methyltransferase OS=Homo sapiens GN=METTL1 PE=1 SV=1
Q9H6K5	PRR36	Proline-rich protein 36 OS=Homo sapiens GN=PRR36 PE=1 SV=2
P78371	TCPB	T-complex protein 1 subunit beta OS=Homo sapiens GN=CCT2 PE=1 SV=4
Q02224	CENPE	Centromere-associated protein E OS=Homo sapiens GN=CENPE PE=1 SV=2
Q969Z4	TR19L	Tumor necrosis factor receptor superfamily member 19L OS=Homo sapiens GN=RELT PE=
O14523	C2C2L	C2 domain-containing protein 2-like OS=Homo sapiens GN=C2CD2L PE=1 SV=3
Q5JV73	FRPD3	FERM and PDZ domain-containing protein 3 OS=Homo sapiens GN=FRMPD3 PE=2 SV=2
O60673	DPOLZ	DNA polymerase zeta catalytic subunit OS=Homo sapiens GN=REV3L PE=1 SV=2
Q9NRU3	CNNM1	Metal transporter CNNM1 OS=Homo sapiens GN=CNNM1 PE=2 SV=3
Q9H8M5	CNNM2	Metal transporter CNNM2 OS=Homo sapiens GN=CNNM2 PE=1 SV=2
P46939	UTRO	Utrophin OS=Homo sapiens GN=UTRN PE=1 SV=2
Q9Y2H2	SAC2	Phosphatidylinositide phosphatase SAC2 OS=Homo sapiens GN=INPP5F PE=1 SV=3
Q8WYP5	ELYS	Protein ELYS OS=Homo sapiens GN=AHCTF1 PE=1 SV=3
Q86TC9	MYPN	Myopalladin OS=Homo sapiens GN=MYPN PE=1 SV=2
Q9Y657	SPIN1	Spindlin-1 OS=Homo sapiens GN=SPIN1 PE=1 SV=3
Q13485	SMAD4	Mothers against decapentaplegic homolog 4 OS=Homo sapiens GN=SMAD4 PE=1 SV=1
P62308	RUXG	Small nuclear ribonucleoprotein G OS=Homo sapiens GN=SNRPG PE=1 SV=1
A8MWD9	RUXGL	Putative small nuclear ribonucleoprotein G-like protein 15 OS=Homo sapiens GN=SNRPG
Q14957	NMDE3	Glutamate receptor ionotropic NMDA 2C OS=Homo sapiens GN=GRIN2C PE=1 SV=3
Q07864	DPOE1	DNA polymerase epsilon catalytic subunit A OS=Homo sapiens GN=POLE PE=1 SV=5
P07814	SYEP	Bifunctional glutamate/proline--tRNA ligase OS=Homo sapiens GN=EPRS PE=1 SV=5
Q9NQB0	TF7L2	Transcription factor 7-like 2 OS=Homo sapiens GN=TCF7L2 PE=1 SV=2
Q9NUP9	LIN7C	Protein lin-7 homolog C OS=Homo sapiens GN=LIN7C PE=1 SV=1
Q9UHI6	DDX20	Probable ATP-dependent RNA helicase DDX20 OS=Homo sapiens GN=DDX20 PE=1 SV=2
Q9BZZ5	API5	Apoptosis inhibitor 5 OS=Homo sapiens GN=API5 PE=1 SV=3
Q8NHZ8	CDC26	Anaphase-promoting complex subunit CDC26 OS=Homo sapiens GN=CDC26 PE=1 SV=1
Q8IUG5	MY18B	Unconventional myosin-XVIIIb OS=Homo sapiens GN=MYO18B PE=1 SV=1
Q92733	PRCC	Proline-rich protein PRCC OS=Homo sapiens GN=PRCC PE=1 SV=1
Q9Y4D8	HECD4	Probable E3 ubiquitin-protein ligase HECTD4 OS=Homo sapiens GN=HECTD4 PE=1 SV=5
Q9BXP8	PAPP2	Pappalysin-2 OS=Homo sapiens GN=PAPPA2 PE=1 SV=4
Q15750	TAB1	TGF-beta-activated kinase 1 and MAP3K7-binding protein 1 OS=Homo sapiens GN=TAB1

Q9UBS9	SUCO	SUN domain-containing ossification factor OS=Homo sapiens GN=SUCO PE=1 SV=1
Q8NEE6	FXL13	F-box/LRR-repeat protein 13 OS=Homo sapiens GN=FBXL13 PE=2 SV=3
Q9H4Q3	PRD13	PR domain zinc finger protein 13 OS=Homo sapiens GN=PRDM13 PE=2 SV=2
Q6VMQ6	MCAF1	Activating transcription factor 7-interacting protein 1 OS=Homo sapiens GN=ATF7IP PE=1
Q6UXN9	WDR82	WD repeat-containing protein 82 OS=Homo sapiens GN=WDR82 PE=1 SV=1
Q5K4L6	S27A3	Long-chain fatty acid transport protein 3 OS=Homo sapiens GN=SLC27A3 PE=2 SV=3
O95777	LSM8	U6 snRNA-associated Sm-like protein LSm8 OS=Homo sapiens GN=LSM8 PE=1 SV=3
Q06546	GABPA	GA-binding protein alpha chain OS=Homo sapiens GN=GABPA PE=1 SV=1
Q7Z6J9	SEN54	tRNA-splicing endonuclease subunit Sen54 OS=Homo sapiens GN=TSEN54 PE=1 SV=3
P49327	FAS	Fatty acid synthase OS=Homo sapiens GN=FASN PE=1 SV=3
Q8IYE1	CCD13	Coiled-coil domain-containing protein 13 OS=Homo sapiens GN=CCDC13 PE=1 SV=2
P20248	CCNA2	Cyclin-A2 OS=Homo sapiens GN=CCNA2 PE=1 SV=2
Q9NVI1	FANCI	Fanconi anemia group I protein OS=Homo sapiens GN=FANCI PE=1 SV=4
Q9UKI8	TLK1	Serine/threonine-protein kinase tousled-like 1 OS=Homo sapiens GN=TLK1 PE=1 SV=2
P48382	RFX5	DNA-binding protein RFX5 OS=Homo sapiens GN=RFX5 PE=1 SV=1
O95996	APC2	Adenomatous polyposis coli protein 2 OS=Homo sapiens GN=APC2 PE=1 SV=1
Q9UBB5	MBD2	Methyl-CpG-binding domain protein 2 OS=Homo sapiens GN=MBD2 PE=1 SV=1
P11387	TOP1	DNA topoisomerase 1 OS=Homo sapiens GN=TOP1 PE=1 SV=2
Q14789	GOGB1	Golgin subfamily B member 1 OS=Homo sapiens GN=GOLGB1 PE=1 SV=2
P09496	CLCA	Clathrin light chain A OS=Homo sapiens GN=CLTA PE=1 SV=1
Q76NI1	VKIND	Protein very KIND OS=Homo sapiens GN=KNDC1 PE=2 SV=2
Q12772	SRBP2	Sterol regulatory element-binding protein 2 OS=Homo sapiens GN=SREBF2 PE=1 SV=2
Q9HCE0	EPG5	Ectopic P granules protein 5 homolog OS=Homo sapiens GN=EPG5 PE=1 SV=2
O95644	NFAC1	Nuclear factor of activated T-cells cytoplasmic 1 OS=Homo sapiens GN=NFATC1 PE=1 SV=
Q5VZP5	DUS27	Inactive dual specificity phosphatase 27 OS=Homo sapiens GN=DUSP27 PE=2 SV=1
Q8NH83	OR4A5	Olfactory receptor 4A5 OS=Homo sapiens GN=OR4A5 PE=3 SV=4
Q9BZZ2	SN	Sialoadhesin OS=Homo sapiens GN=SIGLEC1 PE=1 SV=2
Q9Y2A7	NCKP1	Nck-associated protein 1 OS=Homo sapiens GN=NCKAP1 PE=1 SV=1
Q9NQ48	LZTL1	Leucine zipper transcription factor-like protein 1 OS=Homo sapiens GN=LZTFL1 PE=1 SV=1
P25054	APC	Adenomatous polyposis coli protein OS=Homo sapiens GN=APC PE=1 SV=2
Q5SW79	CE170	Centrosomal protein of 170 kDa OS=Homo sapiens GN=CEP170 PE=1 SV=1
P0C860	MS3L2	Putative male-specific lethal-3 protein-like 2 OS=Homo sapiens GN=MSL3P1 PE=5 SV=1
O43422	P52K	52 kDa repressor of the inhibitor of the protein kinase OS=Homo sapiens GN=THAP12 PE=
O15027	SC16A	Protein transport protein Sec16A OS=Homo sapiens GN=SEC16A PE=1 SV=3
Q5TIE3	VW5B1	von Willebrand factor A domain-containing protein 5B1 OS=Homo sapiens GN=VWA5B1 P
Q03052	PO3F1	POU domain class 3 transcription factor 1 OS=Homo sapiens GN=POU3F1 PE=2 SV=3
Q9C0A1	ZFHX2	Zinc finger homeobox protein 2 OS=Homo sapiens GN=ZFHX2 PE=2 SV=3
Q5U651	RAIN	Ras-interacting protein 1 OS=Homo sapiens GN=RASIP1 PE=1 SV=1
Q9BTC8	MTA3	Metastasis-associated protein MTA3 OS=Homo sapiens GN=MTA3 PE=1 SV=2
O60312	AT10A	Probable phospholipid-transporting ATPase VA OS=Homo sapiens GN=ATP10A PE=2 SV=2
Q6UXI9	NPNT	Nephronectin OS=Homo sapiens GN=NPNT PE=2 SV=3

Q96EB1	ELP4	Elongator complex protein 4 OS=Homo sapiens GN=ELP4 PE=1 SV=2
Q96L96	ALPK3	Alpha-protein kinase 3 OS=Homo sapiens GN=ALPK3 PE=2 SV=2
tr	G3V2T6	Uncharacterized protein (Fragment) OS=Homo sapiens PE=4 SV=2
Q9H6D7	HAUS4	HAUS augmin-like complex subunit 4 OS=Homo sapiens GN=HAUS4 PE=1 SV=1
Q9NYF0	DACT1	Dapper homolog 1 OS=Homo sapiens GN=DACT1 PE=1 SV=2
P23610	F8I2	Factor VIII intron 22 protein OS=Homo sapiens GN=F8A1 PE=1 SV=2
Q8NER1	TRPV1	Transient receptor potential cation channel subfamily V member 1 OS=Homo sapiens GN=
P00751	CFAB	Complement factor B OS=Homo sapiens GN=CFB PE=1 SV=2
tr	B4E1Z4	Uncharacterized protein OS=Homo sapiens PE=1 SV=1
