# Supplementary material for: Polypyrimidine tract binding proteins PTBP1 and PTBP2 interact with distinct proteins under splicing conditions
Source: PLoS One. 2022 Feb 3;17(2):e0263287. doi: 10.1371/journal.pone.0263287 (PMC8812845; doi:10.1371/journal.pone.0263287)
Supplement: S3 Table — A list of proteins identified in the pull-down sample in the presence of His6 tagged PTBP2 under Buffer DG conditions. (PDF) [file pone.0263287.s003.pdf]

UniProtID	Gene	Description
Q9UKA9	PTBP2	Polypyrimidine tract-binding protein 2 OS=Homo sapiens GN=PTBP2 PE=1 SV=1
P04264	K2C1	Keratin type II cytoskeletal 1 OS=Homo sapiens GN=KRT1 PE=1 SV=6
P35908	K22E	Keratin type II cytoskeletal 2 epidermal OS=Homo sapiens GN=KRT2 PE=1 SV=2
P35527	K1C9	Keratin type I cytoskeletal 9 OS=Homo sapiens GN=KRT9 PE=1 SV=3
P13645	K1C10	Keratin type I cytoskeletal 10 OS=Homo sapiens GN=KRT10 PE=1 SV=6
P02533	K1C14	Keratin type I cytoskeletal 14 OS=Homo sapiens GN=KRT14 PE=1 SV=4
P04259	K2C6B	Keratin type II cytoskeletal 6B OS=Homo sapiens GN=KRT6B PE=1 SV=5
P48668	K2C6C	Keratin type II cytoskeletal 6C OS=Homo sapiens GN=KRT6C PE=1 SV=3
P02538	K2C6A	Keratin type II cytoskeletal 6A OS=Homo sapiens GN=KRT6A PE=1 SV=3
P13647	K2C5	Keratin type II cytoskeletal 5 OS=Homo sapiens GN=KRT5 PE=1 SV=3
P02768	ALBU	Serum albumin OS=Homo sapiens GN=ALB PE=1 SV=2
O95678	K2C75	Keratin type II cytoskeletal 75 OS=Homo sapiens GN=KRT75 PE=1 SV=2
Q5XKE5	K2C79	Keratin type II cytoskeletal 79 OS=Homo sapiens GN=KRT79 PE=1 SV=2
P08727	K1C19	Keratin type I cytoskeletal 19 OS=Homo sapiens GN=KRT19 PE=1 SV=4
P08779	K1C16	Keratin type I cytoskeletal 16 OS=Homo sapiens GN=KRT16 PE=1 SV=4
P26599	PTBP1	Polypyrimidine tract-binding protein 1 OS=Homo sapiens GN=PTBP1 PE=1 SV=1
Q04695	K1C17	Keratin type I cytoskeletal 17 OS=Homo sapiens GN=KRT17 PE=1 SV=2
P13646	K1C13	Keratin type I cytoskeletal 13 OS=Homo sapiens GN=KRT13 PE=1 SV=4
P19012	K1C15	Keratin type I cytoskeletal 15 OS=Homo sapiens GN=KRT15 PE=1 SV=3
P12035	K2C3	Keratin type II cytoskeletal 3 OS=Homo sapiens GN=KRT3 PE=1 SV=3
Q7Z3Y8	K1C27	Keratin type I cytoskeletal 27 OS=Homo sapiens GN=KRT27 PE=1 SV=2
Q2M2I5	K1C24	Keratin type I cytoskeletal 24 OS=Homo sapiens GN=KRT24 PE=1 SV=1
P81605	DCD	Dermcidin OS=Homo sapiens GN=DCD PE=1 SV=2
Q7Z794	K2C1B	Keratin type II cytoskeletal 1b OS=Homo sapiens GN=KRT77 PE=2 SV=3
Q7Z3Y7	K1C28	Keratin type I cytoskeletal 28 OS=Homo sapiens GN=KRT28 PE=1 SV=2
P05787	K2C8	Keratin type II cytoskeletal 8 OS=Homo sapiens GN=KRT8 PE=1 SV=7
O95758	PTBP3	Polypyrimidine tract-binding protein 3 OS=Homo sapiens GN=PTBP3 PE=1 SV=2
Q12802	AKP13	A-kinase anchor protein 13 OS=Homo sapiens GN=AKAP13 PE=1 SV=2
Q99456	K1C12	Keratin type I cytoskeletal 12 OS=Homo sapiens GN=KRT12 PE=1 SV=1
P05783	K1C18	Keratin type I cytoskeletal 18 OS=Homo sapiens GN=KRT18 PE=1 SV=2
O76013	KRT36	Keratin type I cuticular Ha6 OS=Homo sapiens GN=KRT36 PE=2 SV=1
Q01546	K22O	Keratin type II cytoskeletal 2 oral OS=Homo sapiens GN=KRT76 PE=1 SV=2
P01861	IGHG4	Ig gamma-4 chain C region OS=Homo sapiens GN=IGHG4 PE=1 SV=1
O76015	KRT38	Keratin type I cuticular Ha8 OS=Homo sapiens GN=KRT38 PE=1 SV=3
Q14525	KT33B	Keratin type I cuticular Ha3-II OS=Homo sapiens GN=KRT33B PE=1 SV=3
Q14532	K1H2	Keratin type I cuticular Ha2 OS=Homo sapiens GN=KRT32 PE=2 SV=3
Q92764	KRT35	Keratin type I cuticular Ha5 OS=Homo sapiens GN=KRT35 PE=2 SV=5
Q8TDM6	DLG5	Disks large homolog 5 OS=Homo sapiens GN=DLG5 PE=1 SV=4
Q8N1N4	K2C78	Keratin type II cytoskeletal 78 OS=Homo sapiens GN=KRT78 PE=2 SV=2
Q86TJ2	TAD2B	Transcriptional adapter 2-beta OS=Homo sapiens GN=TADA2B PE=1 SV=2

P10828	THB	Thyroid hormone receptor beta OS=Homo sapiens GN=THRB PE=1 SV=2
P10827	THA	Thyroid hormone receptor alpha OS=Homo sapiens GN=THRA PE=1 SV=1
Q8TF72	SHRM3	Protein Shroom3 OS=Homo sapiens GN=SHROOM3 PE=1 SV=2
P15924	DESP	Desmoplakin OS=Homo sapiens GN=DSP PE=1 SV=3
Q8WXH0	SYNE2	Nesprin-2 OS=Homo sapiens GN=SYNE2 PE=1 SV=3
P14618	KPYM	Pyruvate kinase PKM OS=Homo sapiens GN=PKM PE=1 SV=4
Q9P225	DYH2	Dynein heavy chain 2 axonemal OS=Homo sapiens GN=DNAH2 PE=2 SV=3
Q7Z3Z0	K1C25	Keratin type I cytoskeletal 25 OS=Homo sapiens GN=KRT25 PE=1 SV=1
Q9UKX3	MYH13	Myosin-13 OS=Homo sapiens GN=MYH13 PE=2 SV=2
Q9ULL4	PLXB3	Plexin-B3 OS=Homo sapiens GN=PLXNB3 PE=1 SV=2
P31271	HXA13	Homeobox protein Hox-A13 OS=Homo sapiens GN=HOXA13 PE=1 SV=3
Q9NW07	ZN358	Zinc finger protein 358 OS=Homo sapiens GN=ZNF358 PE=1 SV=2
Q9BTC0	DIDO1	Death-inducer obliterator 1 OS=Homo sapiens GN=DIDO1 PE=1 SV=5
Q9NSB2	KRT84	Keratin type II cuticular Hb4 OS=Homo sapiens GN=KRT84 PE=2 SV=2
Q7RTS7	K2C74	Keratin type II cytoskeletal 74 OS=Homo sapiens GN=KRT74 PE=1 SV=2
Q3SY84	K2C71	Keratin type II cytoskeletal 71 OS=Homo sapiens GN=KRT71 PE=1 SV=3
Q14CN4	K2C72	Keratin type II cytoskeletal 72 OS=Homo sapiens GN=KRT72 PE=1 SV=2
P08729	K2C7	Keratin type II cytoskeletal 7 OS=Homo sapiens GN=KRT7 PE=1 SV=5
Q9NSB4	KRT82	Keratin type II cuticular Hb2 OS=Homo sapiens GN=KRT82 PE=3 SV=3
Q8TD26	CHD6	Chromodomain-helicase-DNA-binding protein 6 OS=Homo sapiens GN=CHD6 PE=1 SV=4
Q9NRF2	SH2B1	SH2B adapter protein 1 OS=Homo sapiens GN=SH2B1 PE=1 SV=3
Q96JG9	ZN469	Zinc finger protein 469 OS=Homo sapiens GN=ZNF469 PE=2 SV=3
Q8NDA8	MROH1	Maestro heat-like repeat-containing protein family member 1 OS=Homo sapiens GN=MROH1 PE
P01024	CO3	Complement C3 OS=Homo sapiens GN=C3 PE=1 SV=2
Q5T481	RBM20	RNA-binding protein 20 OS=Homo sapiens GN=RBM20 PE=1 SV=3
Q92953	KCNB2	Potassium voltage-gated channel subfamily B member 2 OS=Homo sapiens GN=KCNB2 PE=2 SV=
Q96JB1	DYH8	Dynein heavy chain 8 axonemal OS=Homo sapiens GN=DNAH8 PE=1 SV=2
P84550	SKOR1	SKI family transcriptional corepressor 1 OS=Homo sapiens GN=SKOR1 PE=1 SV=1
Q6PJT7	ZC3HE	Zinc finger CCCH domain-containing protein 14 OS=Homo sapiens GN=ZC3H14 PE=1 SV=1
Q9UID3	VPS51	Vacuolar protein sorting-associated protein 51 homolog OS=Homo sapiens GN=VPS51 PE=1 SV=[illegible]
Q3KR37	GRM1B	GRAM domain-containing protein 1B OS=Homo sapiens GN=GRAMD1B PE=1 SV=1
Q15256	PTPRR	Receptor-type tyrosine-protein phosphatase R OS=Homo sapiens GN=PTPRR PE=1 SV=2
Q8TF61	FBX41	F-box only protein 41 OS=Homo sapiens GN=FBXO41 PE=2 SV=5
Q9HCS5	E41LA	Band 4.1-like protein 4A OS=Homo sapiens GN=EPB41L4A PE=1 SV=2
P54098	DPOG1	DNA polymerase subunit gamma-1 OS=Homo sapiens GN=POLG PE=1 SV=1
Q7Z5P9	MUC19	Mucin-19 OS=Homo sapiens GN=MUC19 PE=1 SV=3
P12883	MYH7	Myosin-7 OS=Homo sapiens GN=MYH7 PE=1 SV=5
P11055	MYH3	Myosin-3 OS=Homo sapiens GN=MYH3 PE=1 SV=3

Q5VST9	OBSCN	Obscurin OS=Homo sapiens GN=OBSCN PE=1 SV=3
P35568	IRS1	Insulin receptor substrate 1 OS=Homo sapiens GN=IRS1 PE=1 SV=1
P25940	CO5A3	Collagen alpha-3(V) chain OS=Homo sapiens GN=COL5A3 PE=1 SV=3
Q9NYQ7	CELR3	Cadherin EGF LAG seven-pass G-type receptor 3 OS=Homo sapiens GN=CELSR3 PE=1 SV=2
Q96A65	EXOC4	Exocyst complex component 4 OS=Homo sapiens GN=EXOC4 PE=1 SV=1
Q9UL36	ZN236	Zinc finger protein 236 OS=Homo sapiens GN=ZNF236 PE=2 SV=2
Q03001	DYST	Dystonin OS=Homo sapiens GN=DST PE=1 SV=4
Q86UL8	MAGI2	Membrane-associated guanylate kinase WW and PDZ domain-containing protein 2 OS=Homo sa SV=3
A6PVC2	TTLL8	Protein monoglycylase TTLL8 OS=Homo sapiens GN=TTLL8 PE=2 SV=4
Q9BXF3	CECR2	Cat eye syndrome critical region protein 2 OS=Homo sapiens GN=CECR2 PE=1 SV=2
Q8NDA2	HMCN2	Hemicentin-2 OS=Homo sapiens GN=HMCN2 PE=2 SV=3
Q6ZUT9	DEN5B	DENN domain-containing protein 5B OS=Homo sapiens GN=DENND5B PE=1 SV=2
Q5JUK3	KCNT1	Potassium channel subfamily T member 1 OS=Homo sapiens GN=KCNT1 PE=1 SV=2
Q6ZR08	DYH12	Dynein heavy chain 12 axonemal OS=Homo sapiens GN=DNAH12 PE=2 SV=2
Q9UQ35	SRRM2	Serine/arginine repetitive matrix protein 2 OS=Homo sapiens GN=SRRM2 PE=1 SV=2
Q8N157	AHI1	Jouberin OS=Homo sapiens GN=AHI1 PE=1 SV=1
Q9NYV4	CDK12	Cyclin-dependent kinase 12 OS=Homo sapiens GN=CDK12 PE=1 SV=2
Q9H6S0	YTDC2	Probable ATP-dependent RNA helicase YTHDC2 OS=Homo sapiens GN=YTHDC2 PE=1 SV=2
Q8NFU7	TET1	Methylcytosine dioxygenase TET1 OS=Homo sapiens GN=TET1 PE=1 SV=2
P40227	TCPZ	T-complex protein 1 subunit zeta OS=Homo sapiens GN=CCT6A PE=1 SV=3
Q9UHN1	DPOG2	DNA polymerase subunit gamma-2 mitochondrial OS=Homo sapiens GN=POLG2 PE=1 SV=1
Q68DE3	USF3	Basic helix-loop-helix domain-containing protein USF3 OS=Homo sapiens GN=USF3 PE=1 SV=3
Q9UKA4	AKA11	A-kinase anchor protein 11 OS=Homo sapiens GN=AKAP11 PE=1 SV=1
Q13435	SF3B2	Splicing factor 3B subunit 2 OS=Homo sapiens GN=SF3B2 PE=1 SV=2
Q9UNF1	MAGD2	Melanoma-associated antigen D2 OS=Homo sapiens GN=MAGED2 PE=1 SV=2
Q9Y446	PKP3	Plakophilin-3 OS=Homo sapiens GN=PKP3 PE=1 SV=1
O00481	BT3A1	Butyrophilin subfamily 3 member A1 OS=Homo sapiens GN=BTN3A1 PE=1 SV=3
Q9ULI0	ATD2B	ATPase family AAA domain-containing protein 2B OS=Homo sapiens GN=ATAD2B PE=1 SV=3
P49750	YLPM1	YLP motif-containing protein 1 OS=Homo sapiens GN=YLPM1 PE=1 SV=3
O15523	DDX3Y	ATP-dependent RNA helicase DDX3Y OS=Homo sapiens GN=DDX3Y PE=1 SV=2
Q9UPU7	TBD2B	TBC1 domain family member 2B OS=Homo sapiens GN=TBC1D2B PE=1 SV=2
Q9P1Z0	ZBTB4	Zinc finger and BTB domain-containing protein 4 OS=Homo sapiens GN=ZBTB4 PE=1 SV=3
Q86VW0	SESD1	SEC14 domain and spectrin repeat-containing protein 1 OS=Homo sapiens GN=SESTD1 PE=1 SV=
Q96KR1	ZFR	Zinc finger RNA-binding protein OS=Homo sapiens GN=ZFR PE=1 SV=2
Q6P9B9	INT5	Integrator complex subunit 5 OS=Homo sapiens GN=INTS5 PE=1 SV=1
P31327	CPSM	Carbamoyl-phosphate synthase [ammonia] mitochondrial OS=Homo sapiens GN=CPS1 PE=1 SV=
O43516	WIPF1	WAS/WASL-interacting protein family member 1 OS=Homo sapiens GN=WIPF1 PE=1 SV=3
Q93009	UBP7	Ubiquitin carboxyl-terminal hydrolase 7 OS=Homo sapiens GN=USP7 PE=1 SV=2
Q8WXI7	MUC16	Mucin-16 OS=Homo sapiens GN=MUC16 PE=1 SV=3
Q12986	NFX1	Transcriptional repressor NF-X1 OS=Homo sapiens GN=NFX1 PE=1 SV=2
Q8NDV7	TNR6A	Trinucleotide repeat-containing gene 6A protein OS=Homo sapiens GN=TNRC6A PE=1 SV=2

Q9UPZ6	THS7A	Thrombospondin type-1 domain-containing protein 7A OS=Homo sapiens GN=THSD7A PE=1 SV=
O60641	AP180	Clathrin coat assembly protein AP180 OS=Homo sapiens GN=SNAP91 PE=1 SV=2
P57058	HUNK	Hormonally up-regulated neu tumor-associated kinase OS=Homo sapiens GN=HUNK PE=1 SV=1
P29966	MARCS	Myristoylated alanine-rich C-kinase substrate OS=Homo sapiens GN=MARCKS PE=1 SV=4
Q5S007	LRRK2	Leucine-rich repeat serine/threonine-protein kinase 2 OS=Homo sapiens GN=LRRK2 PE=1 SV=2
Q9Y4A5	TRRAP	Transformation/transcription domain-associated protein OS=Homo sapiens GN=TRRAP PE=1 SV=
P98164	LRP2	Low-density lipoprotein receptor-related protein 2 OS=Homo sapiens GN=LRP2 PE=1 SV=3
A4D1P6	WDR91	WD repeat-containing protein 91 OS=Homo sapiens GN=WDR91 PE=1 SV=2
A7E2V4	ZSWM8	Zinc finger SWIM domain-containing protein 8 OS=Homo sapiens GN=ZSWIM8 PE=1 SV=1
P58107	EPIPL	Epiplakin OS=Homo sapiens GN=EPPK1 PE=1 SV=2
P48029	SC6A8	Sodium- and chloride-dependent creatine transporter 1 OS=Homo sapiens GN=SLC6A8 PE=1 SV=
P19013	K2C4	Keratin type II cytoskeletal 4 OS=Homo sapiens GN=KRT4 PE=1 SV=4
Q9P2P6	STAR9	StAR-related lipid transfer protein 9 OS=Homo sapiens GN=STARD9 PE=1 SV=3
Q7Z478	DHX29	ATP-dependent RNA helicase DHX29 OS=Homo sapiens GN=DHX29 PE=1 SV=2
Q8IY63	AMOL1	Angiomotin-like protein 1 OS=Homo sapiens GN=AMOTL1 PE=1 SV=1
