# Supplementary material for: Polypyrimidine tract binding proteins PTBP1 and PTBP2 interact with distinct proteins under splicing conditions
Source: PLoS One. 2022 Feb 3;17(2):e0263287. doi: 10.1371/journal.pone.0263287 (PMC8812845; doi:10.1371/journal.pone.0263287)
Supplement: S4 Table — A list of proteins considered common contaminants in mass spectrometry assays. (PDF) [file pone.0263287.s004.pdf]

P1 Hela Contaminant Proteins

UniProtID	Gene	Description
P68032	ACTC	Actin alpha cardiac muscle 1 OS=Homo sapiens GN=ACTC1 PE=1 SV=1
P68366	TBA4A	Tubulin alpha-4A chain OS=Homo sapiens GN=TUBA4A PE=1 SV=1
Q9BVA1	TBB2B	Tubulin beta-2B chain OS=Homo sapiens GN=TUBB2B PE=1 SV=1
Q13885	TBB2A	Tubulin beta-2A chain OS=Homo sapiens GN=TUBB2A PE=1 SV=1
P26038	MOES	Moesin OS=Homo sapiens GN=MSN PE=1 SV=3
Q9NQP4	PFD4	Prefoldin subunit 4 OS=Homo sapiens GN=PFDN4 PE=1 SV=1
O00151	PDLI1	PDZ and LIM domain protein 1 OS=Homo sapiens GN=PDLIM1 PE=1 SV=4
P09497	CLCB	Clathrin light chain B OS=Homo sapiens GN=CLTB PE=1 SV=1
Q05682	CALD1	Caldesmon OS=Homo sapiens GN=CALD1 PE=1 SV=3
A0MZ66	SHOT1	Shootin-1 OS=Homo sapiens GN=SHTN1 PE=1 SV=4
P35611	ADDA	Alpha-adducin OS=Homo sapiens GN=ADD1 PE=1 SV=2
P61758	PFD3	Prefoldin subunit 3 OS=Homo sapiens GN=VBP1 PE=1 SV=3
Q8TF09	DLRB2	Dynein light chain roadblock-type 2 OS=Homo sapiens GN=DYNLRB2 PE=1 SV=1
Q9ULW0	TPX2	Targeting protein for Xklp2 OS=Homo sapiens GN=TPX2 PE=1 SV=2
P05997	CO5A2	Collagen alpha-2(V) chain OS=Homo sapiens GN=COL5A2 PE=1 SV=3
Q12955	ANK3	Ankyrin-3 OS=Homo sapiens GN=ANK3 PE=1 SV=3
O95996	APC2	Adenomatous polyposis coli protein 2 OS=Homo sapiens GN=APC2 PE=1 SV=1
Q99661	KIF2C	Kinesin-like protein KIF2C OS=Homo sapiens GN=KIF2C PE=1 SV=2
O95239	KIF4A	Chromosome-associated kinesin KIF4A OS=Homo sapiens GN=KIF4A PE=1 SV=3
O60610	DIAP1	Protein diaphanous homolog 1 OS=Homo sapiens GN=DIAPH1 PE=1 SV=2
P35221	CTNA1	Catenin alpha-1 OS=Homo sapiens GN=CTNNA1 PE=1 SV=1
P10636	TAU	Microtubule-associated protein tau OS=Homo sapiens GN=MAPT PE=1 SV=5
Q17RW2	COOA1	Collagen alpha-1(XXIV) chain OS=Homo sapiens GN=COL24A1 PE=1 SV=2
Q9NRC6	SPTN5	Spectrin beta chain non-erythrocytic 5 OS=Homo sapiens GN=SPTBN5 PE=1 SV=2
Q8N3U4	STAG2	Cohesin subunit SA-2 OS=Homo sapiens GN=STAG2 PE=1 SV=3
P02462	CO4A1	Collagen alpha-1(IV) chain OS=Homo sapiens GN=COL4A1 PE=1 SV=3
Q68DC2	ANKS6	Ankyrin repeat and SAM domain-containing protein 6 OS=Homo sapiens GN=ANKS[illegible]
O60504	VINEX	Vinexin OS=Homo sapiens GN=SORBS3 PE=1 SV=2
P20929	NEBU	Nebulin OS=Homo sapiens GN=NEB PE=1 SV=5
Q5JU85	IQEC2	IQ motif and SEC7 domain-containing protein 2 OS=Homo sapiens GN=IQSEC2 PE=[illegible]
Q5VST9	OBSCN	Obscurin OS=Homo sapiens GN=OBSCN PE=1 SV=3
P35749	MYH11	Myosin-11 OS=Homo sapiens GN=MYH11 PE=1 SV=3
Q5QGS0	K2022	Protein KIAA2022 OS=Homo sapiens GN=KIAA2022 PE=2 SV=1
Q14324	MYPC2	Myosin-binding protein C fast-type OS=Homo sapiens GN=MYBPC2 PE=1 SV=2
Q8ND23	CARL3	Capping protein Arp2/3 and myosin-I linker protein 3 OS=Homo sapiens GN=CARM[illegible]
P53420	CO4A4	Collagen alpha-4(IV) chain OS=Homo sapiens GN=COL4A4 PE=1 SV=3
O76041	NEBL	Nebulette OS=Homo sapiens GN=NEBL PE=1 SV=1
P98088	MUC5A	Mucin-5AC OS=Homo sapiens GN=MUC5AC PE=1 SV=4

Q6UB99	ANR11	Ankyrin repeat domain-containing protein 11 OS=Homo sapiens GN=ANKRD11 PE=
Q8N3X1	FNBP4	Formin-binding protein 4 OS=Homo sapiens GN=FNBP4 PE=1 SV=3
B0I1T2	MYO1G	Unconventional myosin-Ig OS=Homo sapiens GN=MYO1G PE=1 SV=2
**P2 Hela Contaminant Proteins**		
**UniProtID**	**Gene**	**Description**
P04259	K2C6B	Keratin type II cytoskeletal 6B OS=Homo sapiens GN=KRT6B PE=1 SV=5
O60292	SI1L3	Signal-induced proliferation-associated 1-like protein 3 OS=Homo sapiens GN=SIPA
Q07092	COGA1	Collagen alpha-1(XVI) chain OS=Homo sapiens GN=COL16A1 PE=1 SV=2
Q96JM3	CHAP1	Chromosome alignment-maintaining phosphoprotein 1 OS=Homo sapiens GN=CHA
Q15149	PLEC	Plectin OS=Homo sapiens GN=PLEC PE=1 SV=3
O95613	PCNT	Pericentrin OS=Homo sapiens GN=PCNT PE=1 SV=4
P25067	CO8A2	Collagen alpha-2(VIII) chain OS=Homo sapiens GN=COL8A2 PE=1 SV=2
Q9H2D6	TARA	TRIO and F-actin-binding protein OS=Homo sapiens GN=TRIOBP PE=1 SV=3
P00519	ABL1	Tyrosine-protein kinase ABL1 OS=Homo sapiens GN=ABL1 PE=1 SV=4
Q5JR59	MTUS2	Microtubule-associated tumor suppressor candidate 2 OS=Homo sapiens GN=MTU
Q9NR99	MXRA5	Matrix-remodeling-associated protein 5 OS=Homo sapiens GN=MXRA5 PE=2 SV=3
Q8TE73	DYH5	Dynein heavy chain 5 axonemal OS=Homo sapiens GN=DNAH5 PE=1 SV=3
P11137	MTAP2	Microtubule-associated protein 2 OS=Homo sapiens GN=MAP2 PE=1 SV=4
Q9Y4B5	MTCL1	Microtubule cross-linking factor 1 OS=Homo sapiens GN=MTCL1 PE=1 SV=5
Q15642	CIP4	Cdc42-interacting protein 4 OS=Homo sapiens GN=TRIP10 PE=1 SV=3
Q5VUJ9	EFCB2	EF-hand calcium-binding domain-containing protein 2 OS=Homo sapiens GN=EFCA
Q02388	CO7A1	Collagen alpha-1(VII) chain OS=Homo sapiens GN=COL7A1 PE=1 SV=2
Q96EA4	SPDLY	Protein Spindly OS=Homo sapiens GN=SPDL1 PE=1 SV=2
Q9Y2I6	NINL	Ninein-like protein OS=Homo sapiens GN=NINL PE=1 SV=2
A8MU46	SMTL1	Smoothelin-like protein 1 OS=Homo sapiens GN=SMTNL1 PE=1 SV=1
P08572	CO4A2	Collagen alpha-2(IV) chain OS=Homo sapiens GN=COL4A2 PE=1 SV=4
P12111	CO6A3	Collagen alpha-3(VI) chain OS=Homo sapiens GN=COL6A3 PE=1 SV=5
O94868	FCSD2	F-BAR and double SH3 domains protein 2 OS=Homo sapiens GN=FCHSD2 PE=1 SV=
O94779	CNTN5	Contactin-5 OS=Homo sapiens GN=CNTN5 PE=1 SV=2
Q8N4C6	NIN	Ninein OS=Homo sapiens GN=NIN PE=1 SV=4
