# Supplementary material for: Polypyrimidine tract binding proteins PTBP1 and PTBP2 interact with distinct proteins under splicing conditions
Source: PLoS One. 2022 Feb 3;17(2):e0263287. doi: 10.1371/journal.pone.0263287 (PMC8812845; doi:10.1371/journal.pone.0263287)
Supplement: S5 Table — A list of proteins that co-purified with both PTBP1 and PTBP2 under in vitro splicing conditions containing HeLa nuclear extract. (PDF) [file pone.0263287.s005.pdf]

UniProtID	Gene	Description
P80723	BASP1	Brain acid soluble protein 1 OS=Homo sapiens GN=BASP1 PE=1 SV=2
P54259	ATN1	Atrophin-1 OS=Homo sapiens GN=ATN1 PE=1 SV=3
Q15424	SAFB1	Scaffold attachment factor B1 OS=Homo sapiens GN=SAFB PE=1 SV=4
P82979	SARNP	SAP domain-containing ribonucleoprotein OS=Homo sapiens GN=SARNP PE=1 SV=3
Q13838	DX39B	Spliceosome RNA helicase DDX39B OS=Homo sapiens GN=DDX39B PE=1 SV=1
P62256	UBE2H	Ubiquitin-conjugating enzyme E2 H OS=Homo sapiens GN=UBE2H PE=1 SV=1
Q9UPA5	BSN	Protein bassoon OS=Homo sapiens GN=BSN PE=1 SV=4
Q15811	ITSN1	Intersectin-1 OS=Homo sapiens GN=ITSN1 PE=1 SV=3
Q86YP4	P66A	Transcriptional repressor p66-alpha OS=Homo sapiens GN=GATAD2A PE=1 SV=1
O00170	AIP	AH receptor-interacting protein OS=Homo sapiens GN=AIP PE=1 SV=2
P37235	HPCL1	Hippocalcin-like protein 1 OS=Homo sapiens GN=HPCAL1 PE=1 SV=3
P84074	HPCA	Neuron-specific calcium-binding protein hippocalcin OS=Homo sapiens GN=HPCA PE=1 SV=2
Q9NRG0	CHRC1	Chromatin accessibility complex protein 1 OS=Homo sapiens GN=CHRAC1 PE=1 SV=1
Q9GZU8	F192A	Protein FAM192A OS=Homo sapiens GN=FAM192A PE=1 SV=1
Q9NRF9	DPOE3	DNA polymerase epsilon subunit 3 OS=Homo sapiens GN=POLE3 PE=1 SV=1
P46013	KI67	Proliferation marker protein Ki-67 OS=Homo sapiens GN=MKI67 PE=1 SV=2
Q96EV2	RBM33	RNA-binding protein 33 OS=Homo sapiens GN=RBM33 PE=1 SV=3
