# Supplementary material for: Polypyrimidine tract binding proteins PTBP1 and PTBP2 interact with distinct proteins under splicing conditions
Source: PLoS One. 2022 Feb 3;17(2):e0263287. doi: 10.1371/journal.pone.0263287 (PMC8812845; doi:10.1371/journal.pone.0263287)
Supplement: S6 Table — A list of distinct proteins that co-purified with PTBP1 under in vitro splicing conditions containing HeLa nuclear extract. (PDF) [file pone.0263287.s006.pdf]

UniProtID	Gene	Description
P68104	EF1A1	Elongation factor 1-alpha 1 OS=Homo sapiens GN=EEF1A1 PE=1 SV=1
Q6IS14	IF5AL	Eukaryotic translation initiation factor 5A-1-like OS=Homo sapiens GN=EIF5AL1 PE=2 SV=2
tr	A0A087WWQ2	Uncharacterized protein (Fragment) OS=Homo sapiens PE=4 SV=1
O14979	HNRDL	Heterogeneous nuclear ribonucleoprotein D-like OS=Homo sapiens GN=HNRNPDL PE=1 SV=3
P49005	DPOD2	DNA polymerase delta subunit 2 OS=Homo sapiens GN=POLD2 PE=1 SV=1
Q8WXI9	P66B	Transcriptional repressor p66-beta OS=Homo sapiens GN=GATAD2B PE=1 SV=1
P25685	DNJB1	DnaJ homolog subfamily B member 1 OS=Homo sapiens GN=DNAJB1 PE=1 SV=4
Q96PZ0	PUS7	Pseudouridylate synthase 7 homolog OS=Homo sapiens GN=PUS7 PE=1 SV=2
Q8WVK2	SNR27	U4/U6.U5 small nuclear ribonucleoprotein 27 kDa protein OS=Homo sapiens GN=SNRNP27 PE=1 SV=1
P05387	RLA2	60S acidic ribosomal protein P2 OS=Homo sapiens GN=RPLP2 PE=1 SV=1
Q13547	HDAC1	Histone deacetylase 1 OS=Homo sapiens GN=HDAC1 PE=1 SV=1
Q06830	PRDX1	Peroxiredoxin-1 OS=Homo sapiens GN=PRDX1 PE=1 SV=1
Q14152	EIF3A	Eukaryotic translation initiation factor 3 subunit A OS=Homo sapiens GN=EIF3A PE=1 SV=1
Q9P0W2	HM20B	SWI/SNF-related matrix-associated actin-dependent regulator of chromatin subfamily E member 1-relate SV=1
Q9BVC5	ASHWN	Ashwin OS=Homo sapiens GN=C2orf49 PE=1 SV=1
Q99575	POP1	Ribonucleases P/MRP protein subunit POP1 OS=Homo sapiens GN=POP1 PE=1 SV=2
Q8WXF1	PSPC1	Paraspeckle component 1 OS=Homo sapiens GN=PSPC1 PE=1 SV=1
P42166	LAP2A	Lamina-associated polypeptide 2 isoform alpha OS=Homo sapiens GN=TMPO PE=1 SV=2
P17096	HMGA1	High mobility group protein HMG-I/HMG-Y OS=Homo sapiens GN=HMGA1 PE=1 SV=3
O60869	EDF1	Endothelial differentiation-related factor 1 OS=Homo sapiens GN=EDF1 PE=1 SV=1
Q9ULR0	ISY1	Pre-mRNA-splicing factor ISY1 homolog OS=Homo sapiens GN=ISY1 PE=1 SV=3
Q69YN2	C19L1	CWF19-like protein 1 OS=Homo sapiens GN=CWF19L1 PE=1 SV=2
Q8IUD2	RB6I2	ELKS/Rab6-interacting/CAST family member 1 OS=Homo sapiens GN=ERC1 PE=1 SV=1
Q14686	NCOA6	Nuclear receptor coactivator 6 OS=Homo sapiens GN=NCOA6 PE=1 SV=3
Q8N5I9	CL045	Uncharacterized protein C12orf45 OS=Homo sapiens GN=C12orf45 PE=1 SV=2
Q68CQ4	DIEXF	Digestive organ expansion factor homolog OS=Homo sapiens GN=DIEXF PE=1 SV=2
O00233	PSMD9	26S proteasome non-ATPase regulatory subunit 9 OS=Homo sapiens GN=PSMD9 PE=1 SV=3
O60216	RAD21	Double-strand-break repair protein rad21 homolog OS=Homo sapiens GN=RAD21 PE=1 SV=2
P67775	PP2AA	Serine/threonine-protein phosphatase 2A catalytic subunit alpha isoform OS=Homo sapiens GN=PPP2CA
P62714	PP2AB	Serine/threonine-protein phosphatase 2A catalytic subunit beta isoform OS=Homo sapiens GN=PPP2CB P
Q96BZ8	LENG1	Leukocyte receptor cluster member 1 OS=Homo sapiens GN=LENG1 PE=1 SV=1
Q49AJ0	F135B	Protein FAM135B OS=Homo sapiens GN=FAM135B PE=2 SV=2
O75496	GEMI	Geminin OS=Homo sapiens GN=GMNN PE=1 SV=1
P98082	DAB2	Disabled homolog 2 OS=Homo sapiens GN=DAB2 PE=1 SV=3
Q15020	SART3	Squamous cell carcinoma antigen recognized by T-cells 3 OS=Homo sapiens GN=SART3 PE=1 SV=1
O14910	LIN7A	Protein lin-7 homolog A OS=Homo sapiens GN=LIN7A PE=1 SV=2
Q8N488	RYBP	RING1 and YY1-binding protein OS=Homo sapiens GN=RYBP PE=1 SV=2
P42695	CNDD3	Condensin-2 complex subunit D3 OS=Homo sapiens GN=NCAPD3 PE=1 SV=2
Q9Y605	MOFA1	MORF4 family-associated protein 1 OS=Homo sapiens GN=MRFAP1 PE=1 SV=1
Q92541	RTF1	RNA polymerase-associated protein RTF1 homolog OS=Homo sapiens GN=RTF1 PE=1 SV=4

Q9Y6E0	STK24	Serine/threonine-protein kinase 24 OS=Homo sapiens GN=STK24 PE=1 SV=1
Q7Z5K2	WAPL	Wings apart-like protein homolog OS=Homo sapiens GN=WAPL PE=1 SV=1
O95785	WIZ	Protein Wiz OS=Homo sapiens GN=WIZ PE=1 SV=2
Q8NHW5	RLA0L	60S acidic ribosomal protein P0-like OS=Homo sapiens GN=RPLP0P6 PE=5 SV=1
P05388	RLA0	60S acidic ribosomal protein P0 OS=Homo sapiens GN=RPLP0 PE=1 SV=1
Q8N7X1	RMXL3	RNA-binding motif protein X-linked-like-3 OS=Homo sapiens GN=RBMXL3 PE=2 SV=2
Q14444	CAPR1	Caprin-1 OS=Homo sapiens GN=CAPRIN1 PE=1 SV=2
Q9UFC0	LRWD1	Leucine-rich repeat and WD repeat-containing protein 1 OS=Homo sapiens GN=LRWD1 PE=1 SV=2
P40424	PBX1	Pre-B-cell leukemia transcription factor 1 OS=Homo sapiens GN=PBX1 PE=1 SV=1
P09234	RU1C	U1 small nuclear ribonucleoprotein C OS=Homo sapiens GN=SNRPC PE=1 SV=1
Q15054	DPOD3	DNA polymerase delta subunit 3 OS=Homo sapiens GN=POLD3 PE=1 SV=2
Q13596	SNX1	Sorting nexin-1 OS=Homo sapiens GN=SNX1 PE=1 SV=3
Q96MW1	CCD43	Coiled-coil domain-containing protein 43 OS=Homo sapiens GN=CCDC43 PE=1 SV=2
Q13347	EIF3I	Eukaryotic translation initiation factor 3 subunit I OS=Homo sapiens GN=EIF3I PE=1 SV=1
Q12888	TP53B	Tumor suppressor p53-binding protein 1 OS=Homo sapiens GN=TP53BP1 PE=1 SV=2
Q86UE8	TLK2	Serine/threonine-protein kinase tousled-like 2 OS=Homo sapiens GN=TLK2 PE=1 SV=2
tr	H0YIS7	Protein RNASEK-C17orf49 (Fragment) OS=Homo sapiens GN=RNASEK-C17orf49 PE=4 SV=1
Q9NVI1	FANCI	Fanconi anemia group I protein OS=Homo sapiens GN=FANCI PE=1 SV=4
P40818	UBP8	Ubiquitin carboxyl-terminal hydrolase 8 OS=Homo sapiens GN=USP8 PE=1 SV=1
Q7Z4V5	HDGR2	Hepatoma-derived growth factor-related protein 2 OS=Homo sapiens GN=HDGFRP2 PE=1 SV=1
Q13516	OLIG2	Oligodendrocyte transcription factor 2 OS=Homo sapiens GN=OLIG2 PE=2 SV=2
O75153	CLU	Clustered mitochondria protein homolog OS=Homo sapiens GN=CLUH PE=1 SV=2
Q9Y2L1	RRP44	Exosome complex exonuclease RRP44 OS=Homo sapiens GN=DIS3 PE=1 SV=2
Q8N3F8	MILK1	MICAL-like protein 1 OS=Homo sapiens GN=MICALL1 PE=1 SV=2
P28070	PSB4	Proteasome subunit beta type-4 OS=Homo sapiens GN=PSMB4 PE=1 SV=4
Q96IZ7	RSRC1	Serine/Arginine-related protein 53 OS=Homo sapiens GN=RSRC1 PE=1 SV=1
P31949	S10AB	Protein S100-A11 OS=Homo sapiens GN=S100A11 PE=1 SV=2
Q96D53	ADCK4	AarF domain-containing protein kinase 4 OS=Homo sapiens GN=ADCK4 PE=1 SV=2
Q5T8P6	RBM26	RNA-binding protein 26 OS=Homo sapiens GN=RBM26 PE=1 SV=3
O15523	DDX3Y	ATP-dependent RNA helicase DDX3Y OS=Homo sapiens GN=DDX3Y PE=1 SV=2
O00571	DDX3X	ATP-dependent RNA helicase DDX3X OS=Homo sapiens GN=DDX3X PE=1 SV=3
P52292	IMA1	Importin subunit alpha-1 OS=Homo sapiens GN=KPNA2 PE=1 SV=1
Q9Y3S1	WNK2	Serine/threonine-protein kinase WNK2 OS=Homo sapiens GN=WNK2 PE=1 SV=4
Q86UU0	BCL9L	B-cell CLL/lymphoma 9-like protein OS=Homo sapiens GN=BCL9L PE=1 SV=1
Q9UK45	LSM7	U6 snRNA-associated Sm-like protein LSm7 OS=Homo sapiens GN=LSM7 PE=1 SV=1
O75818	RPP40	Ribonuclease P protein subunit p40 OS=Homo sapiens GN=RPP40 PE=1 SV=3
Q13409	DC1I2	Cytoplasmic dynein 1 intermediate chain 2 OS=Homo sapiens GN=DYNC1I2 PE=1 SV=3
Q7Z2Y8	GVIN1	Interferon-induced very large GTPase 1 OS=Homo sapiens GN=GVINP1 PE=2 SV=2
Q9H477	RBSK	Ribokinase OS=Homo sapiens GN=RBKS PE=1 SV=1
Q562E7	WDR81	WD repeat-containing protein 81 OS=Homo sapiens GN=WDR81 PE=1 SV=2
Q8TAF3	WDR48	WD repeat-containing protein 48 OS=Homo sapiens GN=WDR48 PE=1 SV=1

P35250	RFC2	Replication factor C subunit 2 OS=Homo sapiens GN=RFC2 PE=1 SV=3
Q96QS3	ARX	Homeobox protein ARX OS=Homo sapiens GN=ARX PE=1 SV=1
O95433	AHSA1	Activator of 90 kDa heat shock protein ATPase homolog 1 OS=Homo sapiens GN=AHSA1 PE=1 SV=1
P10809	CH60	60 kDa heat shock protein mitochondrial OS=Homo sapiens GN=HSPD1 PE=1 SV=2
A6NFI3	ZN316	Zinc finger protein 316 OS=Homo sapiens GN=ZNF316 PE=1 SV=1
Q8WWH5	TRUB1	Probable tRNA pseudouridine synthase 1 OS=Homo sapiens GN=TRUB1 PE=1 SV=1
Q8N1G1	REXO1	RNA exonuclease 1 homolog OS=Homo sapiens GN=REXO1 PE=1 SV=3
P33897	ABCD1	ATP-binding cassette sub-family D member 1 OS=Homo sapiens GN=ABCD1 PE=1 SV=2
Q96T58	MINT	Msx2-interacting protein OS=Homo sapiens GN=SPEN PE=1 SV=1
Q9P2K8	E2AK4	eIF-2-alpha kinase GCN2 OS=Homo sapiens GN=EIF2AK4 PE=1 SV=3
Q9P227	RHG23	Rho GTPase-activating protein 23 OS=Homo sapiens GN=ARHGAP23 PE=1 SV=2
O75175	CNOT3	CCR4-NOT transcription complex subunit 3 OS=Homo sapiens GN=CNOT3 PE=1 SV=1
Q99547	MPH6	M-phase phosphoprotein 6 OS=Homo sapiens GN=MPHOSPH6 PE=1 SV=2
O15347	HMGB3	High mobility group protein B3 OS=Homo sapiens GN=HMGB3 PE=1 SV=4
Q9NRS6	SNX15	Sorting nexin-15 OS=Homo sapiens GN=SNX15 PE=1 SV=1
Q6DKJ4	NXN	Nucleoredoxin OS=Homo sapiens GN=NXN PE=1 SV=2
P23467	PTPRB	Receptor-type tyrosine-protein phosphatase beta OS=Homo sapiens GN=PTPRB PE=1 SV=3
O43929	ORC4	Origin recognition complex subunit 4 OS=Homo sapiens GN=ORC4 PE=1 SV=2
Q14978	NOLC1	Nucleolar and coiled-body phosphoprotein 1 OS=Homo sapiens GN=NOLC1 PE=1 SV=2
O75607	NPM3	Nucleoplasmin-3 OS=Homo sapiens GN=NPM3 PE=1 SV=3
Q96RK0	CIC	Protein capicua homolog OS=Homo sapiens GN=CIC PE=1 SV=2
Q01664	TFAP4	Transcription factor AP-4 OS=Homo sapiens GN=TFAP4 PE=1 SV=2
P61024	CKS1	Cyclin-dependent kinases regulatory subunit 1 OS=Homo sapiens GN=CKS1B PE=1 SV=1
Q96SU4	OSBL9	Oxysterol-binding protein-related protein 9 OS=Homo sapiens GN=OSBPL9 PE=1 SV=2
Q9NZR2	LRP1B	Low-density lipoprotein receptor-related protein 1B OS=Homo sapiens GN=LRP1B PE=1 SV=2
Q66K41	Z385C	Zinc finger protein 385C OS=Homo sapiens GN=ZNF385C PE=1 SV=2
Q9BW61	DDA1	DET1- and DDB1-associated protein 1 OS=Homo sapiens GN=DDA1 PE=1 SV=1
Q8N3C0	ASCC3	Activating signal cointegrator 1 complex subunit 3 OS=Homo sapiens GN=ASCC3 PE=1 SV=3
P0DMR1	HNRC4	Heterogeneous nuclear ribonucleoprotein C-like 4 OS=Homo sapiens GN=HNRNPCL4 PE=3 SV=1
O60812	HNRC1	Heterogeneous nuclear ribonucleoprotein C-like 1 OS=Homo sapiens GN=HNRNPCL1 PE=2 SV=1
B7ZW38	HNRC3	Heterogeneous nuclear ribonucleoprotein C-like 3 OS=Homo sapiens GN=HNRNPCL3 PE=2 SV=1
P50238	CRIP1	Cysteine-rich protein 1 OS=Homo sapiens GN=CRIP1 PE=1 SV=3
P56282	DPOE2	DNA polymerase epsilon subunit 2 OS=Homo sapiens GN=POLE2 PE=1 SV=2
Q14676	MDC1	Mediator of DNA damage checkpoint protein 1 OS=Homo sapiens GN=MDC1 PE=1 SV=3
Q86SG6	NEK8	Serine/threonine-protein kinase Nek8 OS=Homo sapiens GN=NEK8 PE=1 SV=1
Q9H8Y8	GORS2	Golgi reassembly-stacking protein 2 OS=Homo sapiens GN=GORASP2 PE=1 SV=3
Q9UMZ3	PTPRQ	Phosphatidylinositol phosphatase PTPRQ OS=Homo sapiens GN=PTPRQ PE=1 SV=2
Q9BX63	FANCJ	Fanconi anemia group J protein OS=Homo sapiens GN=BRIP1 PE=1 SV=1
Q03164	KMT2A	Histone-lysine N-methyltransferase 2A OS=Homo sapiens GN=KMT2A PE=1 SV=5
Q9ULD9	ZN608	Zinc finger protein 608 OS=Homo sapiens GN=ZNF608 PE=1 SV=4
P51825	AFF1	AF4/FMR2 family member 1 OS=Homo sapiens GN=AFF1 PE=1 SV=1

Q6KC79	NIPBL	Nipped-B-like protein OS=Homo sapiens GN=NIPBL PE=1 SV=2
Q9Y6R4	M3K4	Mitogen-activated protein kinase kinase kinase 4 OS=Homo sapiens GN=MAP3K4 PE=1 SV=2
P35968	VGFR2	Vascular endothelial growth factor receptor 2 OS=Homo sapiens GN=KDR PE=1 SV=2
O15067	PUR4	Phosphoribosylformylglycinamidine synthase OS=Homo sapiens GN=PFAS PE=1 SV=4
Q9Y535	RPC8	DNA-directed RNA polymerase III subunit RPC8 OS=Homo sapiens GN=POLR3H PE=1 SV=1
tr	D6RIA3	Protein LOC285556 OS=Homo sapiens GN=LOC285556 PE=4 SV=1
P46087	NOP2	Probable 28S rRNA (cytosine(4447)-C(5))-methyltransferase OS=Homo sapiens GN=NOP2 PE=1 SV=2
Q16891	MIC60	MICOS complex subunit MIC60 OS=Homo sapiens GN=IMMT PE=1 SV=1
O43497	CAC1G	Voltage-dependent T-type calcium channel subunit alpha-1G OS=Homo sapiens GN=CACNA1G PE=1 SV=3
Q9NVE4	CCD87	Coiled-coil domain-containing protein 87 OS=Homo sapiens GN=CCDC87 PE=1 SV=2
O75747	P3C2G	Phosphatidylinositol 4-phosphate 3-kinase C2 domain-containing subunit gamma OS=Homo sapiens GN=[illegible]
P35658	NU214	Nuclear pore complex protein Nup214 OS=Homo sapiens GN=NUP214 PE=1 SV=2
Q15003	CND2	Condensin complex subunit 2 OS=Homo sapiens GN=NCAPH PE=1 SV=3
Q13107	UBP4	Ubiquitin carboxyl-terminal hydrolase 4 OS=Homo sapiens GN=USP4 PE=1 SV=3
P78356	PI42B	Phosphatidylinositol 5-phosphate 4-kinase type-2 beta OS=Homo sapiens GN=PIP4K2B PE=1 SV=1
Q9UD57	NKX12	NK1 transcription factor-related protein 2 OS=Homo sapiens GN=NKX1-2 PE=2 SV=3
P07864	LDHC	L-lactate dehydrogenase C chain OS=Homo sapiens GN=LDHC PE=1 SV=4
Q9NPD3	EXOS4	Exosome complex component RRP41 OS=Homo sapiens GN=EXOSC4 PE=1 SV=3
P63098	CANB1	Calcineurin subunit B type 1 OS=Homo sapiens GN=PPP3R1 PE=1 SV=2
tr	H7BYZ3	Uncharacterized protein OS=Homo sapiens PE=4 SV=1
Q8WWM7	ATX2L	Ataxin-2-like protein OS=Homo sapiens GN=ATXN2L PE=1 SV=2
O75330	HMMR	Hyaluronan mediated motility receptor OS=Homo sapiens GN=HMMR PE=1 SV=2
Q7Z4F1	LRP10	Low-density lipoprotein receptor-related protein 10 OS=Homo sapiens GN=LRP10 PE=1 SV=2
