# Supplementary material for: Polypyrimidine tract binding proteins PTBP1 and PTBP2 interact with distinct proteins under splicing conditions
Source: PLoS One. 2022 Feb 3;17(2):e0263287. doi: 10.1371/journal.pone.0263287 (PMC8812845; doi:10.1371/journal.pone.0263287)
Supplement: S7 Table — A list of distinct proteins that co-purified with PTBP2 under in vitro splicing conditions containing HeLa nuclear extract. (PDF) [file pone.0263287.s007.pdf]

UniProtID	Gene	Description
P17066	HSP76	Heat shock 70 kDa protein 6 OS=Homo sapiens GN=HSPA6 PE=1 SV=2
A5A3E0	POTEF	POTE ankyrin domain family member F OS=Homo sapiens GN=POTEF PE=1 SV=2
Q58FG0	HS905	Putative heat shock protein HSP 90-alpha A5 OS=Homo sapiens GN=HSP90AA5P PE=2 SV=1
Q58FF6	H90B4	Putative heat shock protein HSP 90-beta 4 OS=Homo sapiens GN=HSP90AB4P PE=5 SV=1
P16989	YBOX3	Y-box-binding protein 3 OS=Homo sapiens GN=YBX3 PE=1 SV=4
Q8IYJ1	CPNE9	Copine-9 OS=Homo sapiens GN=CPNE9 PE=1 SV=3
Q13620	CUL4B	Cullin-4B OS=Homo sapiens GN=CUL4B PE=1 SV=4
Q8NEV1	CSK23	Casein kinase II subunit alpha 3 OS=Homo sapiens GN=CSNK2A3 PE=1 SV=2
Q99962	SH3G2	Endophilin-A1 OS=Homo sapiens GN=SH3GL2 PE=1 SV=1
Q9Y2T7	YBOX2	Y-box-binding protein 2 OS=Homo sapiens GN=YBX2 PE=1 SV=2
P43694	GATA4	Transcription factor GATA-4 OS=Homo sapiens GN=GATA4 PE=1 SV=2
Q99453	PHX2B	Paired mesoderm homeobox protein 2B OS=Homo sapiens GN=PHOX2B PE=1 SV=2
P31947	1433S	14-3-3 protein sigma OS=Homo sapiens GN=SFN PE=1 SV=1
P20700	LMNB1	Lamin-B1 OS=Homo sapiens GN=LMNB1 PE=1 SV=2
Q9UHD9	UBQL2	Ubiquilin-2 OS=Homo sapiens GN=UBQLN2 PE=1 SV=2
P35453	HXD13	Homeobox protein Hox-D13 OS=Homo sapiens GN=HOXD13 PE=1 SV=3
Q12852	M3K12	Mitogen-activated protein kinase kinase kinase 12 OS=Homo sapiens GN=MAP3K12 PE=1 SV=2
Q96T37	RBM15	Putative RNA-binding protein 15 OS=Homo sapiens GN=RBM15 PE=1 SV=2
O60346	PHLP1	PH domain leucine-rich repeat-containing protein phosphatase 1 OS=Homo sapiens GN=PHLPP1 PE=1 SV=3
Q01658	NC2B	Protein Dr1 OS=Homo sapiens GN=DR1 PE=1 SV=1
P56270	MAZ	Myc-associated zinc finger protein OS=Homo sapiens GN=MAZ PE=1 SV=1
Q8NB90	SPAT5	Spermatogenesis-associated protein 5 OS=Homo sapiens GN=SPATA5 PE=1 SV=3
Q92777	SYN2	Synapsin-2 OS=Homo sapiens GN=SYN2 PE=2 SV=3
Q8N319	CF223	Uncharacterized protein C6orf223 OS=Homo sapiens GN=C6orf223 PE=2 SV=2
Q9HCK8	CHD8	Chromodomain-helicase-DNA-binding protein 8 OS=Homo sapiens GN=CHD8 PE=1 SV=5
Q1XH10	SKDA1	SKI/DACH domain-containing protein 1 OS=Homo sapiens GN=SKIDA1 PE=2 SV=1
P16220	CREB1	Cyclic AMP-responsive element-binding protein 1 OS=Homo sapiens GN=CREB1 PE=1 SV=2
O75448	MED24	Mediator of RNA polymerase II transcription subunit 24 OS=Homo sapiens GN=MED24 PE=1 SV=1
Q9UI36	DACH1	Dachshund homolog 1 OS=Homo sapiens GN=DACH1 PE=1 SV=3
P05386	RLA1	60S acidic ribosomal protein P1 OS=Homo sapiens GN=RPLP1 PE=1 SV=1
O14654	IRS4	Insulin receptor substrate 4 OS=Homo sapiens GN=IRS4 PE=1 SV=1
Q8TCU4	ALMS1	Alstrom syndrome protein 1 OS=Homo sapiens GN=ALMS1 PE=1 SV=3
Q9BRL6	SRSF8	Serine/arginine-rich splicing factor 8 OS=Homo sapiens GN=SRSF8 PE=1 SV=1
Q5GH72	XKR7	XK-related protein 7 OS=Homo sapiens GN=XKR7 PE=2 SV=1
Q9Y6Q9	NCOA3	Nuclear receptor coactivator 3 OS=Homo sapiens GN=NCOA3 PE=1 SV=1
P23634	AT2B4	Plasma membrane calcium-transporting ATPase 4 OS=Homo sapiens GN=ATP2B4 PE=1 SV=2

Q13207	TBX2	T-box transcription factor TBX2 OS=Homo sapiens GN=TBX2 PE=1 SV=3
Q5VWQ0	RSBN1	Round spermatid basic protein 1 OS=Homo sapiens GN=RSBN1 PE=1 SV=2
Q9BQN1	FA83C	Protein FAM83C OS=Homo sapiens GN=FAM83C PE=1 SV=3
Q9UBW8	CSN7A	COP9 signalosome complex subunit 7a OS=Homo sapiens GN=COPS7A PE=1 SV=1
Q9P2D8	UNC79	Protein unc-79 homolog OS=Homo sapiens GN=UNC79 PE=2 SV=4
Q8WZ19	BACD1	BTB/POZ domain-containing adapter for CUL3-mediated RhoA degradation protein 1 OS=Homo sapiens GN=K[illegible]
Q9P2S2	NRX2A	Neurexin-2 OS=Homo sapiens GN=NRXN2 PE=2 SV=1
P29597	TYK2	Non-receptor tyrosine-protein kinase TYK2 OS=Homo sapiens GN=TYK2 PE=1 SV=3
A6NHT5	HMX3	Homeobox protein HMX3 OS=Homo sapiens GN=HMX3 PE=1 SV=1
Q01804	OTUD4	OTU domain-containing protein 4 OS=Homo sapiens GN=OTUD4 PE=1 SV=4
P25054	APC	Adenomatous polyposis coli protein OS=Homo sapiens GN=APC PE=1 SV=2
Q8N6I1	EID2	EP300-interacting inhibitor of differentiation 2 OS=Homo sapiens GN=EID2 PE=1 SV=3
Q9Y266	NUDC	Nuclear migration protein nudC OS=Homo sapiens GN=NUDC PE=1 SV=1
C9JJ37	BTBDJ	BTB/POZ domain-containing protein 19 OS=Homo sapiens GN=BTBD19 PE=2 SV=1
Q8NHW3	MAFA	Transcription factor MafA OS=Homo sapiens GN=MAFA PE=1 SV=2
Q9H334	FOXP1	Forkhead box protein P1 OS=Homo sapiens GN=FOXP1 PE=1 SV=1
Q86VM9	ZCH18	Zinc finger CCCH domain-containing protein 18 OS=Homo sapiens GN=ZC3H18 PE=1 SV=2
Q9ULD4	BRPF3	Bromodomain and PHD finger-containing protein 3 OS=Homo sapiens GN=BRPF3 PE=1 SV=2
A6NKD9	CC85C	Coiled-coil domain-containing protein 85C OS=Homo sapiens GN=CCDC85C PE=1 SV=1
Q9UJX5	APC4	Anaphase-promoting complex subunit 4 OS=Homo sapiens GN=ANAPC4 PE=1 SV=2
Q12830	BPTF	Nucleosome-remodeling factor subunit BPTF OS=Homo sapiens GN=BPTF PE=1 SV=3
Q32P41	TRM5	tRNA (guanine(37)-N1)-methyltransferase OS=Homo sapiens GN=TRMT5 PE=1 SV=2
O75426	FBX24	F-box only protein 24 OS=Homo sapiens GN=FBXO24 PE=1 SV=2
Q5VVJ2	MYSM1	Histone H2A deubiquitinase MYSM1 OS=Homo sapiens GN=MYSM1 PE=1 SV=1
Q9UIF9	BAZ2A	Bromodomain adjacent to zinc finger domain protein 2A OS=Homo sapiens GN=BAZ2A PE=1 SV=4
Q6NS38	ALKB2	DNA oxidative demethylase ALKBH2 OS=Homo sapiens GN=ALKBH2 PE=1 SV=1
Q9BXM0	PRAX	Periaxin OS=Homo sapiens GN=PRX PE=1 SV=2
Q147X3	NAA30	N-alpha-acetyltransferase 30 OS=Homo sapiens GN=NAA30 PE=1 SV=1
Q10469	MGAT2	Alpha-1 6-mannosyl-glycoprotein 2-beta-N-acetylglucosaminyltransferase OS=Homo sapiens GN=MGAT2 PE=1
O14746	TERT	Telomerase reverse transcriptase OS=Homo sapiens GN=TERT PE=1 SV=1
Q6NSJ2	PHLB3	Pleckstrin homology-like domain family B member 3 OS=Homo sapiens GN=PHLDB3 PE=2 SV=3
Q13233	M3K1	Mitogen-activated protein kinase kinase kinase 1 OS=Homo sapiens GN=MAP3K1 PE=1 SV=4
Q66K64	DCA15	DDB1- and CUL4-associated factor 15 OS=Homo sapiens GN=DCAF15 PE=1 SV=1
P20591	MX1	Interferon-induced GTP-binding protein Mx1 OS=Homo sapiens GN=MX1 PE=1 SV=4
O95069	KCNK2	Potassium channel subfamily K member 2 OS=Homo sapiens GN=KCNK2 PE=1 SV=2
Q8NEZ4	KMT2C	Histone-lysine N-methyltransferase 2C OS=Homo sapiens GN=KMT2C PE=1 SV=3
Q8N8A6	DDX51	ATP-dependent RNA helicase DDX51 OS=Homo sapiens GN=DDX51 PE=1 SV=3
Q96K37	S35E1	Solute carrier family 35 member E1 OS=Homo sapiens GN=SLC35E1 PE=1 SV=2

Q6E0U4	DMKN	Dermokine OS=Homo sapiens GN=DMKN PE=1 SV=3
Q8IWI9	MGAP	MAX gene-associated protein OS=Homo sapiens GN=MGA PE=1 SV=3
O60749	SNX2	Sorting nexin-2 OS=Homo sapiens GN=SNX2 PE=1 SV=2
Q9Y2V3	RX	Retinal homeobox protein Rx OS=Homo sapiens GN=RAX PE=1 SV=2
Q8NFT6	DBF4B	Protein DBF4 homolog B OS=Homo sapiens GN=DBF4B PE=1 SV=1
Q15911	ZFHX3	Zinc finger homeobox protein 3 OS=Homo sapiens GN=ZFHX3 PE=1 SV=2
P18074	ERCC2	TFIIH basal transcription factor complex helicase XPD subunit OS=Homo sapiens GN=ERCC2 PE=1 SV=1
Q15464	SHB	SH2 domain-containing adapter protein B OS=Homo sapiens GN=SHB PE=1 SV=2
O14559	RHG33	Rho GTPase-activating protein 33 OS=Homo sapiens GN=ARHGAP33 PE=1 SV=2
Q15413	RYR3	Ryanodine receptor 3 OS=Homo sapiens GN=RYR3 PE=1 SV=3
A6NKL6	T200C	Transmembrane protein 200C OS=Homo sapiens GN=TMEM200C PE=2 SV=2
Q86WA8	LONP2	Lon protease homolog 2 peroxisomal OS=Homo sapiens GN=LONP2 PE=1 SV=1
P15882	CHIN	N-chimaerin OS=Homo sapiens GN=CHN1 PE=1 SV=3
Q9UBL3	ASH2L	Set1/Ash2 histone methyltransferase complex subunit ASH2 OS=Homo sapiens GN=ASH2L PE=1 SV=1
Q6PID6	TTC33	Tetratricopeptide repeat protein 33 OS=Homo sapiens GN=TTC33 PE=1 SV=2
P54132	BLM	Bloom syndrome protein OS=Homo sapiens GN=BLM PE=1 SV=1
Q9H5V9	CX056	UPF0428 protein CXorf56 OS=Homo sapiens GN=CXorf56 PE=1 SV=1
Q5H9T9	FSCB	Fibrous sheath CABYR-binding protein OS=Homo sapiens GN=FSCB PE=2 SV=3
Q96C24	SYTL4	Synaptotagmin-like protein 4 OS=Homo sapiens GN=SYTL4 PE=1 SV=2
O95793	STAU1	Double-stranded RNA-binding protein Staufen homolog 1 OS=Homo sapiens GN=STAU1 PE=1 SV=2
Q96HP4	OXND1	Oxidoreductase NAD-binding domain-containing protein 1 OS=Homo sapiens GN=OXNAD1 PE=1 SV=1
