# Supplementary material for: Polypyrimidine tract binding proteins PTBP1 and PTBP2 interact with distinct proteins under splicing conditions
Source: PLoS One. 2022 Feb 3;17(2):e0263287. doi: 10.1371/journal.pone.0263287 (PMC8812845; doi:10.1371/journal.pone.0263287)

Loading order has been annotated.
Identity of experimental samples, method used to capture the image –lanes have been labeled.
SDS-PAGE Methods have been described under "methods" in the manuscript. The gel was imaged using an OmegaLum imaging system.
Figure 2 was generated from this original image.
Molecular weight markers are included
Lanes not included in the final figure have been marked with an "X" above the lane label on the original blot/gel image.

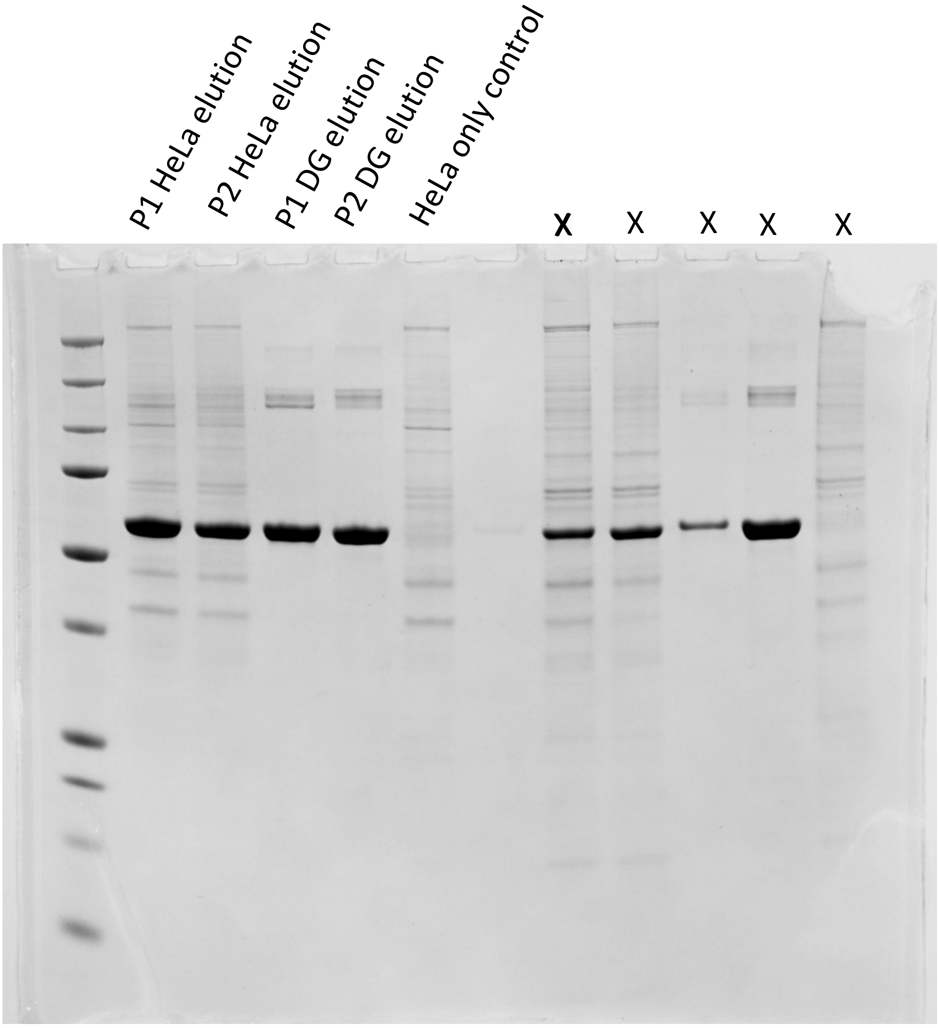

Supplement: S1 Raw images — (PDF) [file pone.0263287.s008.pdf]
